# Supplementary material for: A prospective randomized trial examining health care utilization in individuals using multiple smartphone-enabled biosensors
Source: PeerJ. 2016 Jan 14;4:e1554. doi: 10.7717/peerj.1554 (PMC4715435; doi:10.7717/peerj.1554)
Supplement: Supplemental Information 1 [file peerj-04-1554-s001.pdf]

CONSORT 2010 checklist of information to include when reporting a randomised trial*

Section/Topic	Item No	Checklist item	Reported on page No
**Title and abstract**			
	1a	Identification as a randomised trial in the title	1
	1b	Structured summary of trial design, methods, results, and conclusions (for specific guidance see CONSORT for abstracts)	2-3
**Introduction**			
Background and objectives	2a	Scientific background and explanation of rationale	4-6
	2b	Specific objectives or hypotheses	5-6
**Methods**			
Trial design	3a	Description of trial design (such as parallel, factorial) including allocation ratio	6
	3b	Important changes to methods after trial commencement (such as eligibility criteria), with reasons	NA
Participants	4a	Eligibility criteria for participants	7
	4b	Settings and locations where the data were collected	8-11
Interventions	5	The interventions for each group with sufficient details to allow replication, including how and when they were actually administered	7-9
Outcomes	6a	Completely defined pre-specified primary and secondary outcome measures, including how and when they were assessed	9-10
	6b	Any changes to trial outcomes after the trial commenced, with reasons	NA
Sample size	7a	How sample size was determined	11
	7b	When applicable, explanation of any interim analyses and stopping guidelines	NA
Randomisation:			
Sequence generation	8a	Method used to generate the random allocation sequence	7
	8b	Type of randomisation; details of any restriction (such as blocking and block size)	6-7
Allocation concealment mechanism	9	Mechanism used to implement the random allocation sequence (such as sequentially numbered containers), describing any steps taken to conceal the sequence until interventions were assigned	7
Implementation	10	Who generated the random allocation sequence, who enrolled participants, and who assigned participants to interventions	7
Blinding	11a	If done, who was blinded after assignment to interventions (for example, participants, care providers, those	7

		assessing outcomes) and how	
	11b	If relevant, description of the similarity of interventions	7-9
Statistical methods	12a	Statistical methods used to compare groups for primary and secondary outcomes	11
	12b	Methods for additional analyses, such as subgroup analyses and adjusted analyses	11
**Results**			
Participant flow (a diagram is strongly recommended)	13a	For each group, the numbers of participants who were randomly assigned, received intended treatment, and were analysed for the primary outcome	11-13
	13b	For each group, losses and exclusions after randomisation, together with reasons	11-12
Recruitment	14a	Dates defining the periods of recruitment and follow-up	9-10
	14b	Why the trial ended or was stopped	NA
Baseline data	15	A table showing baseline demographic and clinical characteristics for each group	13
Numbers analysed	16	For each group, number of participants (denominator) included in each analysis and whether the analysis was by original assigned groups	13
Outcomes and estimation	17a	For each primary and secondary outcome, results for each group, and the estimated effect size and its precision (such as 95% confidence interval)	14-15
	17b	For binary outcomes, presentation of both absolute and relative effect sizes is recommended	NA
Ancillary analyses	18	Results of any other analyses performed, including subgroup analyses and adjusted analyses, distinguishing pre-specified from exploratory	16-18
Harms	19	All important harms or unintended effects in each group (for specific guidance see CONSORT for harms)	21
**Discussion**			
Limitations	20	Trial limitations, addressing sources of potential bias, imprecision, and, if relevant, multiplicity of analyses	21
Generalisability	21	Generalisability (external validity, applicability) of the trial findings	21
Interpretation	22	Interpretation consistent with results, balancing benefits and harms, and considering other relevant evidence	19-22
**Other information**			
Registration	23	Registration number and name of trial registry	1
Protocol	24	Where the full trial protocol can be accessed, if available	1
Funding	25	Sources of funding and other support (such as supply of drugs), role of funders	1

*We strongly recommend reading this statement in conjunction with the CONSORT 2010 Explanation and Elaboration for important clarifications on all the items. If relevant, we also recommend reading CONSORT extensions for cluster randomised trials, non-inferiority and equivalence trials, non-pharmacological treatments, herbal interventions, and pragmatic trials. Additional extensions are forthcoming: for those and for up to date references relevant to this checklist, see www.consort-statement.org.

Scripps Wired for Health Study

Research Protocol

Protocol Version:	6.0
Version Date:	28April2014
Sponsor:	Scripps Translational Science Institute<br>3344 North Torrey Pines Court, Suite 300<br>La Jolla, California 92037
Co-Sponsors:	Scripps Health Wellness<br>4275 Campus Point Ct.<br>San Diego, CA 92121<br><br>Health Comp Third Party Administrators<br>P.O. Box 45018<br>Fresno, CA 93718-5018<br><br>Qualcomm Life<br>Qualcomm Incorporated<br>5775 Morehouse Drive<br>San Diego, CA 92121-1714<br><br>Healthy Circles<br>5050 Morehouse Drive<br>San Diego, CA 92121-1714
Principal Investigator:	Eric J. Topol, M.D.
Co-Investigators:	Cinnamon S. Bloss, Ph.D.<br>Ravi Komatireddy, M.D.<br>Paddy Barrett, M.D.<br>Nicholas J. Schork, Ph.D.<br>Carlos Meier, M.A., M.P.I.A.
Study Coordinators:	Judith Sheard, M.P.H., C.C.R.A.<br>Melissa Peters, B.A.

1.0 BACKGROUND

Chronic diseases such as hypertension, diabetes mellitus, and cardiac arrhythmias, continue to represent a significant healthcare burden on both patients and the healthcare system as a whole. The high prevalence of these conditions combined with well characterized complications that result in significant, negative impacts to quality of life has led to the appropriation of significant scientific and engineering resources to find ways to improve diagnosis and treatment that include advances in diagnostic technology and pharmaceuticals (ALLHAT, Ernst 2003, Uzu 2005).

Despite these efforts, the management of these conditions remains challenging (Hansen 2005). Patient engagement and adherence to outpatient medications and treatment strategies is poor (Guyatt 1986, Hansen 2005). Furthermore, patients' understanding of their disease condition, communication between them and appropriate health care providers, and their ability to identify early warning signs prior to clinical decompensation events is questionable. From the clinician perspective, appropriate tools that can provide the insight necessary to help manage chronic disease in the outpatient setting are lacking. These challenges have resulted in clinical and economic consequences such as disease progression in patients with poor control of chronic conditions, high utilization of emergency departments, inpatient resources, and readmissions to medical centers (Sander 2005).

Two related technology areas, wireless medicine and telehealth, may provide the tools necessary to improve care and reduce healthcare costs for a variety of common conditions. Using mobile computing platforms such as smartphones, internet connectivity, and novel biomedical sensors, wireless medical devices can measure disease metrics that can be analyzed for patterns and be used by both patients and providers to make proactive healthcare decisions. Previous work using wireless health telemedicine technology has shown significant improvements in disease management for hypertension (HTN), diabetes mellitus (DM), arrhythmias (Budinger 2003, Clifford et al. 2012, Darkins et al. 2008, Rothman 2007). Other research using a tool to enable remote assessment of patients with a several chronic conditions helped to identify those patients that needed direct clinician intervention, but in a proactive manner.

The latest generation of wireless health devices sport improvements in accuracy, usability, and compatibility with ubiquitous smartphones and clinical software. Additionally, the availability of smartphones, the evolution of algorithms used to analyze health data, and techniques for data visualization may improve patient and clinician interpretation of health data, disease management, and outpatient detection of physiologic states such as clinically significant arrhythmia. Given the base of evidence for telemedicine for outpatient disease management and the features of the latest wireless health technology it would be useful to evaluate the feasibility of using a suite of smartphone compatible wireless health devices on patients with hypertension, diabetes mellitus, and cardiac arrhythmias.

2.0 STUDY DESIGN

2.1 Purpose

We propose to evaluate the impact of using a smartphone enabled "Wireless Monitoring System" in conjunction with a disease management program on the healthcare costs and resource utilization of chronically ill individuals with diabetes, hypertension, and cardiac arrhythmia.

The intervention consists of:

- Wireless medical devices (glucometer, blood pressure monitor, and/or mobile ECG)
- Healthy Circles online patient portal and connected care coordination platform (branded for the study as the "Health Comp" online portal)
- Disease management program managed by Health Comp.

We aim to specifically target our wireless monitoring intervention to be used by chronic disease "hot spotters;" the small number of patients identified, through medical billing data, to account for the highest healthcare spending and resource utilization.

We predict that the benefits to patients from using wireless monitoring will be increased understanding of their disease, increased health self-management (e.g., self-efficacy), more transparency regarding their chronic conditions, and improved care coordination by staff members in the wellness program. Ultimately, we further hypothesize that this may result in improved therapeutic adherence, proactive medical choices, and better management of their chronic conditions as measured by disease specific physiologic markers. We hypothesize that these benefits directly translate to decreased acute care visits, decreased inpatient health service utilization (hospital admissions, days of hospitalization) and decreased outpatient health service utilization (emergency department visits, need-based primary care clinic visits, and need-based specialty clinic visits) and will ultimately lead to significant reductions in health care costs for the treatment group relative to a control group and relative to pre-monitoring utilization rates. Study participants will be currently enrolled in or willing to be enrolled in Health Comp's Disease Management program.

This study is a collaboration between Scripps Translational Science Institute, Scripps Wellness, Qualcomm Life, Health Comp (the third party administrator for Scripps Health responsible for processing all health care insurance claims for employees and dependents) and Healthy Circles. This study is designed as a pilot study to determine the feasibility of deploying this type of intervention with multiple chronic disease groups, and to inform the design of a larger trial to further assess the impact and effectiveness of wireless monitoring interventions.

2.2 Preliminary Data

The principles of biomonitoring including a survey of device technologies, methods, and barriers have been studied (Budinger 2003, Clifford et al. 2012). The benefits of decreased health service utilization in particular through wireless monitoring have been shown by the Veterans Administration (VA) with a 0.7% decrease in health utilization over a cohort of 1963 patients (Darkins et al. 2008). Cell phones, in particular, have been extensively studied in their role as facilitators of telehealth; a systematic review of 25 studies encompassing 38,060 patients that used cell phones for outpatient health monitoring for a variety of conditions showed improvements in hemoglobin A1c in diabetics, improved control of asthma symptoms, better medication adherence, and process improvements (Krishna et al. 2009).

Furthermore, individual studies and meta-analyses focused on telehealth monitoring of diabetes, hypertension, and arrhythmia have also shown significant promise. A large meta-analysis of 21 studies using cell phones for the management of diabetes, 8 of which were powered to measure significant DM outcomes, showed significant promise in terms of improved glycemic control, self-efficacy, and hemoglobin A1c (Holtz et al. 2012). Furthermore, ambulatory electrocardiographic monitoring has also demonstrated the ability to detect clinically significant arrhythmias in the outpatient setting (Rothman et al. 2007). Finally, sustained improvements in the control of hypertension using home monitoring and management of blood pressure have been demonstrated in a number of previous trials (McManus et al. 2010, Wakefield et al. 2011).

We have evaluated available demographic and billing pattern data from Health Comp pertaining to our target populations for this trial. From a denominator of 28,425 active members, there were 21,691 individuals who had at least one claim in 2012. From this number, there were 4,060 individuals who billed for CPT codes related to hypertension, diabetes, and/or arrhythmias. After excluding individuals under the age of 18 years, a total of 3,999 individuals remained, and we have shown a breakdown of demographic and billing metrics for individuals in each of our target condition categories in the table below. These data suggest the utility of this data source for identifying our "hotspotter" target disease populations and the sampling frame from which we will recruit the participants who will comprise our study sample.

Table 1.

Total N = 3999	Hypertension	Diabetes	Arrhythmia	Co-morbid (2 or more)
N (% of Total) §	3222 (80.6%)	1304 (32.6%)	651 (16.3%)	1087 (27.2%)
Age (Mean Years)	54.4 (10.9)	53.7 (11.4)	52.4 (14.1)	56.5 (10.2)
Office Visits (Mean #, SD/Median)	6.8 (6.1)/5	7.2 (6.6)/6	9.1 (7.9)/7	8.2 (7.1)/6
Condition Specific Costs (Mean $, SD/Median)	2,894 (10,310)/312	3,628 (12,269)/521	5,189 (23,445)/179	N/A
Total Costs (Mean $, SD/Median)	10,699 (30,095)/2,265	10,532 (31,954)/2,206	24,901 (52,058)/6,301	16,364 (41,944/3,133

2.3 Experimental Flow

This study will utilize a randomized controlled design. Health Comp will identify Scripps plan members with a history of diagnostic billing codes consistent with either diabetes (insulin dependent diabetes and non-insulin dependent diabetes), hypertension or cardiac arrhythmia. They will be recruited from a list of individuals associated with the highest utilization rates and costs over the past two 6-month periods. A sufficient number of participants – we estimate approximately 1,000 - will be contacted to yield a total of 200 study subjects. Of the individuals in this group, 100 will be randomized to receive the wireless monitoring intervention (Monitoring Group) plus Health Comp Disease Management program and 100 will be randomized to receive the standard Health Comp Disease Management program only (Control Group).

The participants randomized to the Monitoring Group will be provided with an iPhone 4 or 4s, access to the Healthy Circles portal and program, and the wireless device(s) that are appropriate for monitoring their particular medical diagnoses. Patients will use the wireless monitoring system for a period of six months. As was done for the two 6-month periods prior to the intervention, health service utilization rates will be obtained for the six months during the wireless monitoring intervention period.

All patients will also be enrolled in the Health Comp disease management program which involves outreach by Health Comp nursing staff for the purposes of relaying medical education and wellness information with regard to disease prevention and chronic disease management.

Upon completion of the study, participants in both groups will receive a $20 gift card, in addition to 32 Scripps Health Wellness points for participation in the study. If an employee earns 100 Wellness points in a calendar year (through participating in other Scripps Wellness activities), they are eligible for a reduction in health insurance costs.

These wellness points will be earned in the following categories:

- 10 credits for Health Coaching (there is a 10 credit maximum per year)
- 4 credits for Completion of the Baseline Survey and Follow Up Survey (there is a 4 credit maximum for survey completion each year)
- 18 credits for Personal Health Activities (there is a 36 credit maximum per year)

Wellness points for the study will be awarded at the end of the fiscal year – September 2014.

A diagram of the study design can be found in Appendix 1.

2.4 Methods

Recruitment and Consent:

In order to provide awareness of the study, an e-mail was sent from Human Resources to all Scripps Health employees (Appendix 20).

Health Comp and Scripps Health have established a protocol for Health Comp to contact members (Scripps employees and their dependents) to discuss their medical conditions and health status. To identify these members, Health Comp conducts regular queries of their medical billing database to identify those members who have medical claims consistent with chronic disease.

For the purposes of this study, members who are identified as having one or more of the targeted chronic diseases (diabetes, hypertension or cardiac arrhythmia) with high medical resource utilization will be sent a letter (Appendix 2) introducing Health Comp and informing the member that they will be contacted by Health Comp staff to discuss their health and medications. Within 2 to 4 weeks, a Health Comp staff member (typically a RN or LVN) will follow up with a telephone call to the identified patients (Appendix 3). Patients will also be given the option of contacting Health Comp. The staff member will discuss the patient's medical status and introduce the Disease Management program. A description of the standard Disease Management Program already in use by Health Comp is included in Appendix 4. The member then has the option of joining the program or opting out of any further calls or contact.

If a patient joins the Disease Management program and they meet the inclusion/exclusion criteria of the study, the nurse will also present the opportunity for them to participate in the trial. The study will be thoroughly discussed, including participants' responsibilities and rights, risks and benefits, and method for withdrawing.

The nurse will explain that upon completion of the call, the patient will receive a link to the Informed Consent form via e-mail or 2 paper consent forms along with a letter of explanation will be sent by mail. They will be asked to carefully read the document and will be given a phone number to call if they have any additional questions regarding the study. Patients who read the consent and have no additional questions will be asked to electronically sign the consent form or sign the paper copy. They will keep one for their records and mail the other to HealthComp in a self-addressed stamped envelope (provided). If, after 10 business days, HealthComp has not received the returned consent, a staff member will reach out to the participant. After completing the informed consent, they will be directed to an on-line baseline survey (Appendix 5). For participants completing the paper consent, a link to the baseline survey will be sent to the member via email. HealthComp will send a copy of the consent form to the Study Coordinator. Surveys will also be completed 6 months, and the survey will be similar to the Baseline survey (Appendix 21). If a patient expresses an interest in participating in the study but does not register online and complete the consent form, four attempts to re-engage the patient will be made via phone call and/or email. If after four attempts no confirmation of participation has been established with the patient, this will be considered a "screen failure." Any patient who elects not to participate in the study will still be offered participation in the Disease Management program outside the auspices of the study.

After study staff have received notification that the patient has signed the Informed Consent form and completed the baseline survey, a staff member will call the patient again and schedule the Enrollment visit. This visit will be conducted by a Scripps Research Coordinator or Research Assistant at a Scripps facility convenient to the patient. **It will be explained to the patient that Scripps Health (employer) will not have access to their medical or clinical information that is used for the study**. The data collected for the study, including survey data, usage data from the online data platform, and biometric information collected from the wireless devices, will be used for study purposes only. He/she will also understand that if they choose not to participate, this visit will be cancelled and there will be no further contact regarding the study. (The patient may still elect to participate in the Disease Management program which includes communication with Health Comp staff but without the wireless monitoring system provided by the study.)

Randomization
Patients will be randomized to one of two study groups (Monitoring group or Control group) after they sign the consent form and complete the baseline survey, They will be notified of their group assignment at the Enrollment visit.

Health Data and Assessments:
The data for this study will be collected via online web-based surveys, electronic medical records, Healthy Circles web portal and Health Comp billing data. Biometric data, including blood pressure and blood glucose will be collected at both Enrollment and Termination visits with study staff.

The Study Coordinator/Assistant (Scripps employees) will review the patient's medical records and verify hospital admissions, days of hospitalization, emergency department visits, need-based primary care clinic visits, need-based specialty clinic visits lab results and procedures. Biometric readings of blood pressure, blood glucose, HgA1c, and ECG readings will also be recorded and used for data analysis. Only data related to the study's endpoints will be recorded and utilized.

The online Survey platform "Survey Monkey" and the Healthy Circles software platform will be utilized. Surveys will be administered at Baseline, and at completion of monitoring (6-months from start). An email will be sent to the participants half way through the study to extend them an opportunity to give feedback.

The survey will include measurement of: Demographics, Health History, and Health Literacy; (b) Health Care Resource Consumption (self-reported) and Health Self-Management (Health Locus of Control, Health Self-Efficacy, and Patient Activation); (c) Health-related Quality of Life, Physician-Patient Communication, and Medication Adherence; and (d) Physical Activity/Exercise and Attitudes towards technology (Innovativeness and Personal Involvement). In the Monitoring Group only, the survey will additionally include measures of (e) Device/system Usability, Satisfaction with Device (CSI 6-items), Utility of Monitoring, and Motivation for Tracking/compliance.

Schedule of Events: The schedule of events for the study is as follows:

ALL POTENTIAL PARTICIPANTS

Initial Outreach Letter (Health Comp)
- Introduce partnership of Scripps and Health Comp
- Invite to join Disease Management program.
- Present study and review risks, benefits and responsibilities.
- List options for contacting Health Comp, if interested in study.

Screening Telephone Call (Health Comp)
- Discuss Disease Management program.
- Present study and review risks, benefits and responsibilities.
- Complete screening form, with eligibility criteria, if patient interested in study

Patient On-line Activity (Patient, Health Comp as needed)
- Review Informed Consent form and call Health Comp nurse with any additional questions about study.
- Sign Informed Consent form.
- Complete Baseline Survey.

Post-Consent Telephone Call (Scripps Study Coordinator)
- Schedule enrollment visit.
- Patient randomized to Monitoring Group or Control.

PARTICIPANTS RANDOMIZED TO MONITORING GROUP

Enrollment Visit (Scripps Study Coordinator): Patients will come to one of the Scripps Enrollment Visit sites 1-4 weeks after consent obtained.

- Review study requirements.
- Sign Terms of Use agreements for Healthy Circles portal, iPhone, iTunes and device(s).
- Complete Baseline Survey if not already completed.
- Enroll patient in Healthy Circles.
- Train patient on use of iPhone and/or app(s).
- Train patient on use of device.
- Record baseline readings for device that patient will use during study (blood pressure, ECG, or blood glucose) (for Diabetics also obtain HgA1c)

Telephone Call to Patient (7-10 days after Enrollment Visit) (Health Comp)

- Confirm connectivity and answer questions about operation of devices.
- Introduce the Health Comp Disease Management Program (distinct from Healthy Circles but with overlap in the educational materials used)
- Confirm study participation

Telephone Calls to Patient (at any time during the study) (Health Comp)

- According to current Health Comp protocol, this occurs when patients are non-compliant with picking up prescriptions from the pharmacy or getting recommended lab tests.

Healthy Circle Messages to Patient (at any time during the study) (sent electronically)

- According to a pre-determined protocol (see section below on Monitoring and Log-on Schedule) patients will receive messages on their i-Phone or computer via the Healthy Circles platform if they fail to take the prescribed number of weekly readings for their particular device(s).

3-month E-mail to Patient (Scripps Study Coordinator)

- Extend the participant an opportunity to provide feedback on their experience as a study participant.

6-month Follow-up Telephone Call to Patient (Health Comp)

- Schedule final termination visit.
- Set expectation that they will get link via email to do final survey.
- Discuss continuation in Disease Management Program (distinct from Healthy Circles).

6-month E-mail to Patient (Health Comp)

- Remind participant to complete on-line 6-month survey.

Final Study visit (Scripps Coordinator)

- Return study devices
- Record final readings for devices that patient used during study (for Diabetics also obtain HgA1c).
- Have participant to complete 6-month survey during the visit, if not already completed
- Inform patient that32 Wellness points will be awarded in September 2014 and award a gift card ($20)

PATIENTS RANDOMIZED TO CONTROL GROUP

Enrollment Visit (Scripps Study Coordinator): Patients will come to one of the Scripps Enrollment Visit sites 1-4 weeks after consent obtained to:

- Review study requirements.
- Complete Baseline Survey if not already completed
- Record baseline readings of the measurements appropriate for patient (blood pressure, ECG, or blood glucose) (for Diabetics also obtain HgA1c).

Telephone Call to Patient (7-10 days after enrollment visit) (Health Comp)

- Introduce the Health Comp Disease Management Program.
- Confirm study participation

Telephone Calls to Patient (at any time during the study) (Health Comp)

- According to current Health Comp protocol, this occurs when patients are non-compliant with picking up prescriptions from the pharmacy or getting recommended lab tests.

3-month E-mail to Patient (Scripps Study Coordinator)

- Extend the participant an opportunity to provide feedback on their experience as a study participant.

6-month Follow-up Telephone Call to Patient (Health Comp)

- Schedule final termination visit.
- Set expectation that they will get link via email to do final survey.
- Discuss continuation in Disease Management Program.

6-month E-mail to Patient (Health Comp)

- Remind participant to complete on-line 6-month survey.

Final Study visit (Scripps Coordinator)

- Record measurements appropriate for patient (blood pressure, ECG and/or blood glucose) (for Diabetics also obtain HgA1c).
- Have participant to complete 6-month survey during the visit, if not already completed. Inform patient that 32 Wellness points will be awarded in September 2014 and award a gift card ($20)

Healthy Circles On-line Platform:

Healthy Circles is an iPhone connected care coordination platform, which includes an integrated suite of online Care Management and Consumer Portals that can deliver chronic disease educational information and link patients to their families, caregivers, and healthcare professionals. Through a collaboration with Qualcomm Life's 2net platform, Healthy Circles also now includes a dashboard that displays the patient's device monitoring results and trends over time. (For the purposes of this study, the website will be branded with the Health Comp logo.) Data from the device(s) will be wirelessly uploaded to the patient's Healthy Circles account and accessible via the patient's i-phone or computer. Also included in the platform will be resources which provide information about the patient's disease condition and standard general health behavior recommendations.

iPhone and Wireless Devices

All patients in the monitoring group will be provided with an iPhone 4 for the purposes of the study. Participants will return the study iPhone and devices at the end of the study.

Portal and Device Training and Patient Support:

As shown, patients randomized to the monitoring group will be trained on how to use their study iPhone, the HealthyCircles portal, and their device(s) at their Enrollment Visit. Patients will also be provided with a contact email and phone number they can use to reach a study staff member for first tier technical and other study support.

The devices for the study include:

1. iBGStar - iPhone enabled capillary blood glucose meter. The iBGStar is an iPhone hardware extension that functions as a conventional, outpatient glucometer using specialized test strips and lancets, which will be provided to the participant, to measure blood glucose from a patient's peripheral blood using a finger stick. Accuracy, speed, and method of operation are comparable to current outpatient glucometers. The device is designed to seamlessly integrate with an iPhone to visualize and store blood glucose readings. However, the device does not require an iPhone for operation and can be used as a stand-alone glucometer. The device has FDA 510(k) clearance.

2. Withings Blood Pressure Monitor - iPhone enabled blood pressure monitor. This automated blood pressure cuff uses an internal battery source, separate from an iPhone, to initiate an oscillometric measurement of systolic and diastolic blood pressure. Again, accuracy and speed of measurement is comparable to other automated outpatient blood pressure cuffs. The method of operation includes connecting the cuff to an iPhone and initiating the measurement through the iPhone user interface. A software algorithm controls inflation and deflation of the cuff as well as determination of blood pressure which is displayed on the iPhone screen. The device has 510(k) clearance by the FDA.

3. AliveCor - iPhone enabled ECG monitor. This device uses the iPhone for data processing, visualization, and recording of a single lead ECG signal when its two sensors are held against the chest by the patient or between the fingertips. The ECG signal captured by the device can be recorded and reviewed post measurement. Specifically, AliveCor will provide an interpretation of every reading taken by the patient through AliveCor's partnership with e-cardio, within 30 minutes from when the measurement was taken. That interpretation will then be viewable by the patient in his or her HealthyCircles portal. A Health Comp Nurse will review the Healthy Circles portal each business day to see if an ecardio report has surfaced, if so, the nurse will call the patient within the next business day. AliveCor received FDA 510(k) clearance in December 2012.

See Appendices for of Use Agreements, End User License Agreements and Device agreements

Monitoring and Log-on Schedule

Patients will be requested to monitor their health condition at regular intervals as summarized in Table 2 below. If their monitoring falls below the level defined in the "Poor Compliance" range, they will receive an e-mail on the Healthy Circles platform and their personal e-mail and/or a text message on their study phone reminding them of the monitoring schedule. After a pre-defined time period of no readings, the patient will receive a call from a Health Comp nurse.

Call from Health Comp Nurse

The purpose of this call will be to discuss the patient's participation in the study. If the patient is interested in continuing and has had difficulties with their device(s) or the program, the nurse will talk to the patient about reasons for non-participation and will brainstorm strategies for overcoming schedule issues, device problems, logging on to the portal, etc. If the patient indicates that s/he wants to stop study, the Health Comp nurse will notify the Scripps study coordinator and s/he will call the patient to set up a time to return the devices.

Table 2: Device Monitoring Schedule (HTN=Hypertension group, NIDDM=Non-insulin dependent Diabetes Group, and IDDM=Insulin-dependent diabetes group)

Device	Study Frequency	Poor Compliance	Patient Initiated Monitoring
HTN: Withings	2 x per day, 3 days per	< 3 x per week for 2	Symptoms including but not limited to

BP	week, 1st one in a.m.	consecutive weeks	visual disturbance, "bounding" pulse, chest discomfort, nausea
NIDDM: iBG Star	1 x per day (pre-meal), 3 x per week	< 3 x per week in 1 week	Symptoms including but not limited to fatigue, visual changes, pre-syncopal symptoms, dyspnea, nausea, vomiting
IDDM: iBG Star	4 x per day (pre-meal, with 4th reading at bedtime, 2 hours after last meal/dinner), every day	< 4 x per day for 3 days in 1 week	Symptoms including but not limited to fatigue, visual disturbance, pre-syncopal symptoms, dyspnea, nausea, vomiting
Arrhythmia: Alive Cor ECG	if symptomatic	< 1 reading in 2 weeks will trigger message to confirm lack of symptoms, for 30 seconds	Symptoms including but not limited to chest discomfort, palpitations, rapid heart rate, feeling of "skipped beats," dyspnea, nausea, pre-syncopal symptoms

Hypertension

All patients in the hypertension intervention group will be asked to take their blood pressure at least 3 times per week during the study. If a patient reaches the non-compliance level as outlined in Table 2, they will receive the following message on their message board:

> For the purposes of the study, please take your blood pressure a minimum of 2 times per day, 3 days per week with the first measurement in the morning. In addition, you should also take your blood pressure if you experience visual changes, "bounding pulse", chest discomfort or nausea. Thank you for your continued participation.

If a patient has an additional instance of non-compliance (<3 readings per week for 2 consecutive weeks), the Health Comp nurse will call them and discuss their participation and brainstorm solutions to problems that might be preventing the patient from taking their blood pressure. .

Measurements that meet the following criteria will trigger a message from the Health Comp nurse that contains hypertension educational content related to medication compliance, diet, exercise etc.
>140/90mmHg on 2 measures in 1 week for 2 consecutive weeks

> We have noticed that lately you have had 2 or more blood pressure readings that are considered elevated. Please check out the documents in the Resources tab for ways to reduce your blood pressure and improve your heart health.

Non-insulin Dependent Diabetes Mellitus

All patients in the non-insulin dependent diabetes intervention group will be asked to measure their blood sugar according the frequency in Table 2. If a patient reaches the non-compliance level as outlined in Table 2, they will receive the following message on their message board:

> For the purposes of the study, please check your blood sugar a minimum of 1 time per day (pre-meal), 3 days per week. In addition, you should also check your blood sugar if you experience fatigue, visual changes, dizziness, light headededness, shortness of breath, nausea or vomiting. Thank you for your continued participation.

If a patient has an additional instance of non-compliance (< 3 reading per week for 1 week), the Health Comp nurse will call them and discuss their participation and brainstorm solutions to problems that might be preventing the patient from taking their blood glucose.

Over a 48 or 72 hour time period, measurements that meet either of the following criteria will trigger a message from the Health Comp nurse.

- Any 2 or more readings >250mg/dl over a consecutive 48 hour period.
- Any 2 or more readings <65mg/dl over a consecutive 72 hour period.

> We have noticed that lately you have had 2 or more blood glucose readings that are considered elevated. Please check out the documents in the Resrouces tab for ways to reduce your blood glucose and improve your health.

<u>Insulin Dependent Diabetes Mellitus Group</u>

All patients in the insulin dependent diabetes intervention group will be asked to measure their blood sugar according the frequency in Table 2. If a patient reaches the non-compliance level as outlined in Table 2, they will receive the following message on their message board:

> For the purposes of the study, please check your blood sugar at minimum before every meal and again at bedtime. In addition, you should also check your blood sugar if you experience fatigue, visual changes, dizziness, light headedness, shortness of breath, nausea or vomiting. Thank you for your continued participation.

If a patient has an additional instance of non-compliance (< 4 readings per day in 1 week), the Health Comp nurse will call them and discuss their participation and brainstorm solutions to problems that might be preventing the patient from taking their glucose reading.

Over a 48 or 72 hour time period, measurements that meet either of the following criteria will trigger a message from the Health Comp nurse.

- Any 2 or more readings > 250mg/dl over a consecutive 48 hour period.
- Any 2 or more readings <65mg/dl over a consecutive 72 hour period.

> We have noticed that lately you have had 2 or more blood glucose readings that are considered elevated. Please check out the documents in the Resources tab for ways to reduce your blood glucose and improve your health.

Arrhythmia

All patients in the arrhythmia intervention group will be asked to take an ECG only when symptomatic. If a patient does not record an ECG in 2 weeks, the following message will be sent to the patient's message board on the Health Comp portal.

> Thank you for your participation in the Wired for Health study. We have asked you to take an ECG if you have any symptoms (chest discomfort, palpitations, rapid heart rate, feeling of "skipped beats," light headedness, "bounding pulse", shortness of breath, visual changes or nausea). Since we haven't heard from you, we're assuming that you remain symptom free. Will you please confirm this by sending us a return message? Click on "Reply All" in the lower right hand corner of the message and just let us know you haven't had any symptoms that needed monitoring. Thank you very much.

If a patient does not reply to the message 3 times (6 weeks of no readings or replies), the Health Comp nurse will call them and discuss their participation and educate them about the process of responding to an e-mail.
When a patient has symptoms and s/he takes a reading with the AliceCor ECG, they will be asked to take it for a full 30 seconds. The ECG is uploaded to eCardio, a cardiac monitoring service and read by a physician. At the enrollment visit the patient will be instructed to log-in to their Health Comp portal approximately 30 minutes after taking a reading, to view the report. (See Appendix 19 for a sample report.)

The report will summarize the measurements recorded on the ECG. The patient will be instructed to note the analysis of the current event as either "Stable," "Serious," or "Critical." They will have received previous instructions and documentation with the following recommendations:

- Stable: This rhythm is stable. However, if you feel unwell in any way or have any symptoms of concern please contact a medical provider.
- Serious: This is a serious rhythm. Consultation with a medical provider is advised.
- Critical: Immediate medical consultation is advised.

Within 72 hours, the Health Comp nurse will call the patient to insure they received the report, have read it and understand it. Once a patient has demonstrated that they are logging on and reviewing the report, the Health Comp nurse will no longer call them.

<u>Patients will be reminded at the enrollment visit that their measurements are not being viewed in real time, so should they experience symptoms that they feel require medical attention, they should call 911 or go to the emergency room. The readings conducted for the study do not replace the acute medical attention that a patient may need at the onset of symptoms.</u>

<u>Data Aggregation and Access:</u>

Via connection with Qualcomm's 2net platform, Healthy Circles will aggregate all readings from the wireless devices. The 2net platform, a wireless hardware hub, supplied by Qualcomm, will act as the home gateway from the iPhone to the Healthy Circles portals. Data security for the phone will consist of password protection for general iPhone access as well as an additional layer of security consisting of a login, to access the HealthyCircles portal. These measures, in addition to the data encryption methods employed by the devices and software platforms are HIPAA secure. Similarly, a log-in will be required to access the HealthyCircles portal through conventional computer Internet access. For the selected wireless devices, data will be transmitted through the iPhone's cellular network, aggregated at the 2net platform, and sent as a consolidated secure stream to the Health Comp branded HealthyCircles platform. Transport methods supported by each sensor provider will be leveraged in order to collect data securely from the iPhone, and aggregate at the 2net platform.

As is currently the case as part of the Disease Management program, Health Comp will review data for patients in both groups for noncompliance with prescription refills, preventive screenings, etc. They will either send a "deficit letter" or call the patient (according to their current protocol) to alert him/her of the finding.

<u>Follow-up Surveys</u>

Patients will receive e-mails to complete the 6 month survey 7 days before the anticipated due date. If they haven't completed the survey 7 days after the due date, a reminder e-mail will be sent. An additional reminder will be sent on day 14. On day 21, the Health Comp nurse will make a call to remind the patient, and if necessary, assist the patient with logging into the survey and completing it. If the patient is unable to complete the survey at the end of study visit they will be given the option to complete it at the visit. If the patient indicates that they do not wish to take the survey, they will be "withdrawn."

2.5 Endpoints

2.5.1 Primary Endpoints

1. Health Care Resource Consumption as measured by:
    a. Total costs billed for specific condition (HComp claims), as well as total costs billed
    b. Total health care visits and ER, Inpatient stays (hospitalizations), and office visits individually (HComp claims)
    c. Self-reported utilization (Stanford PERC Measure 4-item scale)
2. Health Self-Management as measured by:

a. Health Locus of Control (MHLC Form C 18-items)
b. Health Self-Efficacy (Stanford PERC Measure 6-item scale)
c. Patient Activation (Patient Activation Measure 13-items)

2.5.2 Secondary Endpoints

1. Health-related Quality of Life (SF-12 Short Form)
2. Biometric Measures (change in BP and/or blood glucose/HgA1c between Enrollment and Termination)
3. Physician Communication (Modified Medical Communication Competence Scale)
4. Medication Adherence (Morisky 8-item measure)

2.5.3 Exploratory Outcomes

1. Physical Activity/Exercise (GLTEQ 3-items)
2. Attitudes towards technology (Diffusion of Innovations, Personal Involvement Inventory)

(Only in intervention group)

3. Device/system usability (SUS 10-items)
4. Satisfaction with device (CSI 6-items)
5. Utility of Monitoring (TBD)
6. Motivation for tracking/compliance (TBD)
7. Correlations between wireless device readings/trends and primary and secondary endpoints

(Healthy Circles Google analytics and iforms)

8. Family engagement (HCircles invites to join care team)
9. Physician engagement (HCircles invites to join care team)
10. Education uptake (time spent on educational materials)
11. Data engagement (time spent viewing own data)

3.0 SUBJECT SELECTION

3.1 Number of Subjects

The study will enroll up to 250 individuals; 100 will be randomized to the monitoring group and 100 to the control groups.

Importantly, this pilot study is designed to leverage the full cohort (as a whole) to make a broader generalization as to the feasibility and potential utility of wireless monitoring across conditions and devices. The study is not powered, nor is its purpose, to definitively assess the utility of monitoring for each specific disease group. Data from each sub-group of participants, however (diabetes, hypertension, and arrhythmias), will be analyzed to identify trends for each disease group that will be used to inform future studies.

3.2 Nature of Study Population

Participants will be selected from among Scripps Health insured employees and family members whose healthcare benefits are administered via Health Comp.

3.3 Inclusion Criteria

1. Scripps Health insured employee or adult family member covered by Scripps Health plan
2. Ability to attend two visits (one at the beginning of the study and one at the end) at a Scripps facility
3. Computer and internet access, as well as ability to use e-mail and text messaging
4. Can grant permission for study staff to access medical records
5. Participating in Health Comp Disease Management program or willingness to join
6. English speaking
7. Within the past 12 months, a history of billing insurance for diagnostic codes consistent with diabetes, hypertension, and/or cardiac arrhythmia
8. 18 years and older
9. Willingness to use wireless devices, study iPhone, and learn to use the Healthy Circles platform.

3.4 Exclusion Criteria

1. Related to or household sharing with another study participant (if there are two or more people in one household that are eligible, the member that has the highest utilization will be invited to join first)
2. Change in living and/or employment situation that dictates the participant will no longer be covered by Scripps Health plan
3. Major surgery or extended trips in the next 6 months that may interfere with consistent use of monitoring device
4. Implanted ICD, pacemaker or implanted loop recorder or expected to undergo insertion during study
5. Arm circumference larger than that accommodated by Withings cuff
6. Unwilling or unable to grant informed consent
7. Pregnancy

3.5 Coding of Data Collected

All participant contact and data collection will be performed by Health Comp staff and Scripps Study Coordinators/Assistants. All data collected as part of the study, as well as medical and clinical information obtained from the patient's medical record will be used for study purposes only.

4.0 DISCONTINUED SUBJECTS

4.1 Study Withdrawal

Participants may withdraw at any time at their own request, or they may be withdrawn at any time at the discretion of the investigators for safety, or administrative reasons. If the subject requests to terminate their participation early, there will be no penalty or loss of benefits to which they are otherwise entitled. Any data that has been entered in the database will be included in the study analysis.

5.0 RISKS

Adverse Events

The likelihood of an adverse event with this intervention and study design is extremely low. Any concern regarding patient safety will be addressed by the study staff.

Risks Associated with Devices

- iBGStar – The risks associated with the glucose monitor include soreness to the finger used for fingersticks. The finger may become red and, very rarely, infected.
- Withings Blood Pressure Monitor –no risks.
- AliveCor ECG Monitor – no risks.

Psychological or Social Risks Associated with Loss of Privacy
While we believe that the risks to the participant associated with loss of privacy are low, we are unable to tell the participant exactly what all of the risks are. The participant's privacy is very important to us and we will use many safety measures to protect their privacy. However, in spite of all of the safety measures that we will use, we cannot guarantee that their identity will never become known. While the controlled-access databases specifically developed for this project will not contain information that is traditionally used to identify the participant, such as their name, address, and telephone number, people may develop ways in the future that would allow someone to link the participant's medical information back to them. There also may be other privacy risks that we have not foreseen.

Risks to Community or Group
Information on the participant's ethnic and geographical background will be included with other information about them in published research or a database developed for this study. In future studies, researchers may find that certain health problems appear more often in people from some ethnic groups than in people from other ethnic groups. This may make some people look down on the participant's ethnic group unfairly. Biology does not provide a reason for prejudice, but discrimination does exist. We will work to make sure that the ethnic or geographic identity of your community is described as carefully as possible in articles that project researchers write based on this research, but we cannot completely control how this information is described in publications that others write.

6.0 RISK ANALYSIS

Participants may or may not benefit from participating in this study, and may quit at any time. Study investigators, Scripps Translational Science Institute, Health Comp, Qualcomm, Scripps Health, and/or The Scripps Research Institute might benefit financially from this study. If any of these parties make a profitable product from the data collected or results obtained from this study, there is no plan to share any of the profits with the participants. The possible financial benefit to the institutions involved in the research will not affect the patient's safety or negatively impact the scientific quality of the study. If patients would like more information they may ask the study staff at Scripps or Health Comp.

The wireless monitoring data as well as the survey information collected from each participant will be labeled with a unique identifier. Study investigators at Health Comp and Scripps Translational Science Institute will keep the information that matches the code to traditionally used identifying information in a safeguarded database.

There is a low likelihood of any adverse events with this study, and wireless monitoring involves very little risk, therefore, the risk-benefit ratio is favorable.

6.1 Data Accessibility

The Principal Investigator, Sub-investigators, Research staff, and approved scientific research collaborators will have access to the participant's research information obtained in the context of this study. Only Health Comp and Scripps Translational Science Institute study staff will have access to participants' traditionally-used identifying information, such as name, address, and telephone number and medical information collected from the wireless monitoring system.

De-identified research information from analyses of the coded wireless monitoring data may be put into databases accessible by the Internet or other state-of-the-art standard method used by scientists to transmit data. The information in

this database will be available only to researchers who have received approval from a Data Access Committee composed of experts in the field.

The data may be made available to the Federal Food and Drug Administration, the National Institute of Health, the Department of Health and Human Services Agencies, Scripps Health, and The Scripps Research Institute, and other Institutional Investigators who have obtained permission from committees that review research to protect individuals.

6.2 Broad Data Sharing

This research is a collaborative effort and the data generated from this research may become available to other researchers in the future. This information could be put into scientific databases that are accessible to several research collaborators. Future researchers may use the information to study other questions about disease. The future researchers may include researchers at universities, hospitals, non-profit groups and other companies. Such researchers, just as with this project, will have to follow all the laws and guidelines that apply to biomedical research.

7.0 STATISTICAL METHODOLOGY

Data Analysis and Interpretation: We will analyze data from online web-based surveys, electronic medical records, and screening and enrollment visits, as well as data collected via the Healthy Circles software platform, Health Comp claims data, and possibly online portals specific to the devices used by patients for monitoring. We will leverage the benefits of a "gold standard" prospective randomized controlled design. Since randomization is being utilized, the treatment and control groups should not differ on important individual characteristics. In the event, however, that the groups are imbalanced on any key covariates, we will use propensity scores to control for this.

Importantly, we will compare outcomes:
1) Between the treatment and control groups, as well as
2) Within the treatment group between the pre-monitoring and on-monitoring periods for health resource utilization based on claims data.

At the end of the study, any change (e.g., reduction) in health service utilization achieved in the treatment group will be summarized statistically to yield a quantitative estimate of the potential effectiveness of wireless monitoring for lower health care costs and utilization rates. Our other primary and secondary endpoints will be treated in a similarly quantitative/statistical fashion.

Sample Size Justification for Pilot Study: Here we show the results of a power analysis to inform our sample size determination. As shown, to compare the difference in, e.g., clinic visits between the treatment and control groups using a repeated measures between factors ANOVA, at a sample size of 80 individuals in each group (conservatively accounting for attrition), we will have 95% power at alpha of 5% to detect a 1 visit difference between the groups assuming a standard deviation of 2 visits. The figure below depicts our statistical power as a function of sample size assuming 2 groups, 2 repeated measures (pre-monitoring and on-monitoring), an effect size of 0.25 and an alpha level of 0.05.

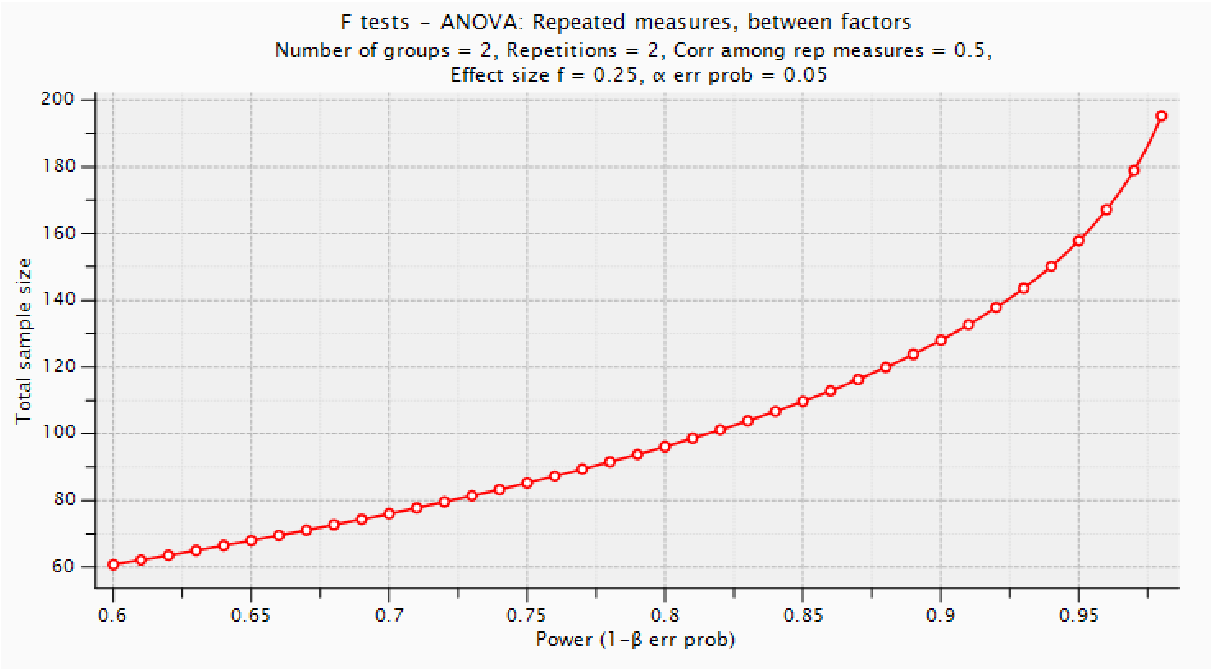

This study's target accrual is 100 individuals randomized to the wireless monitoring intervention and 100 individuals randomized to the control group. We will have the resources to potentially recruit our full target sample size within a 6-month period.

No interim analysis to adjust sample size is planned.

8.0 Informed Consent

Study staff at Health Comp will identify eligible participants. A Health Comp staff member will contact eligible participants and ask about their interest in participating in the study. If the participant is interested, he or she will be e-mailed a link to the Informed Consent form and asked to review and electronically sign the ICF. This will take place prior to any study procedure being performed as stated in the SOPRS policy 4.02. The participant will have as much time as needed to read, understand, and ask questions regarding the study and consent form. If the participant has any additional questions or concerns, they will be able to contact additional study staff for clarification. Once the participant signs the informed consent form, he/she will have an opportunity to print the consent form for his or her records.

The Principal Investigator, the Sub-Investigators, and the Clinical Research Coordinators have completed the informed consent module as required by SOPRS.

9.0 Termination of the Clinical Trial

This will occur when the predetermined number of subjects has had all final assessments completed as outlined in the protocol, and all data have been analyzed. Based on the study design, the investigators do not anticipate a scenario that would trigger early termination.

10.0 Record Retention Requirements

De-identified clinical information will be stored in a safeguarded database that is password protected, which will be located at STSI. The study information may be kept indefinitely, but the study investigators will be required to keep trial records for a period of up to two years.

11.0 MONITORING

11.1 Oversight of Study

This study will obtain Institutional Review Board Approval with Scripps Health.

11.2 Quality Assurance

During the study, collected data will be monitored for omitted data, gross data inconsistencies, illegible data, and deviations. Any deficiencies or deviations will be reviewed and any necessary action determined (e.g. data query). The data cleaning Cycle will be repeated until all data are considered clean. Incremental computer data back-up will be performed on a regular basis. Passwords will be issued to appropriate personnel to ensure confidentiality and protection of data.

11.3 Personnel Responsibilities

The Principal Investigator (PI) is an individual, who actually conducts a clinical investigation under whose immediate direction a product, i.e., drug or device, is administered or dispensed to a subject. The PI is the responsible leader for the team of individuals conducting the study. An exhaustive list of responsibilities is detailed in the Investigator's Agreement document.

11.3.1 Overview of Principal Investigator's Roles and Responsibilities

- Conduct the study according to ICH Guideline on Good Clinical Practice (E6), FDA requirements including 21CFR, Parts 11, 50, 54, 56, 812, and local requirements;
- Oversee all study-related medical decisions;
- Permit monitor(s) to inspect facilities and subject and study records;
- Permit FDA and sponsor inspections of facilities and records, if applicable;
- Maintain protocol adherence, comprehensive source documents, 100% product accountability, data validity;
- Submit protocol, informed consent, annual progress reports, final reports, and AE reports to the IRB and to the sponsor;
- Submit amendments to the protocol and informed consent to the IRB for approval, unless the changes reduce the risk to the subjects;
- Provide adequate medical care for any study AE and inform the subject when medical care is needed for any concurrent illness;
- Carefully explain the correct use of study product to the subject;
- Obtain written consent/assent;
- Document and inform ongoing subjects of revised information on the consent;
- Obtain and maintain IRB approval for the protocol and consent/assent;
- Maintain an adequate number of appropriately qualified staff and adequate facilities;
- Ensure all relevant clinical licensure is current for all study staff;
- Maintain the list of signatures documenting the delegation of study-related duties;
- Document and explain any deviation from the approved protocol;
- Review and sign eCRFs to ensure accuracy and completeness;
- Sign source notes to demonstrate review and agreement;
- Refrain from promoting study or study articles unless approved by STSI;

- Retain records for 2 years following completion of the study.

12.0 PUBLICATION POLICY

This trial will be registered with ClinicalTrials.gov, and as such will obtain a clinicaltrials.gov identifier.

12.1 Reporting

Study investigators will also seek to publish any findings in peer reviewed journals.

13.0 REFERENCES

(2000). "Major cardiovascular events in hypertensive patients randomized to doxazosin vs. chlorthalidone: the antihypertensive and lipid-lowering treatment to prevent heart attack trial (ALLHAT). ALLHAT Collaborative Research Group." JAMA 283(15): 1967-1975.

(2002). "Major outcomes in high-risk hypertensive patients randomized to angiotensin-converting enzyme inhibitor or calcium channel blocker vs. diuretic: The Antihypertensive and Lipid-Lowering Treatment to Prevent Heart Attack Trial (ALLHAT)." JAMA 288(23): 2981-2997.

Andersen, M. J., W. Khawandi, et al. (2005). "Home blood pressure monitoring in CKD." Am J Kidney Dis 45(6): 994-1001.

Ben-Dov, I. Z., J. D. Kark, et al. (2007). "Predictors of all-cause mortality in clinical ambulatory monitoring: unique aspects of blood pressure during sleep." Hypertension 49(6): 1235-1241.

Boggia, J., Y. Li, et al. (2007). "Prognostic accuracy of day versus night ambulatory blood pressure: a cohort study." Lancet 370(9594): 1219-1229.

Budinger, T. F. (2003). "Biomonitoring with Wireless Communications." Annu. Rev. Biomed. Eng. 5: 383-412.

Clifford G. D., Clifton D. (2012). "Wireless Technology in Disease Management and Medicine." Annu. Rev. Med. 63:479-92.

Cook D. J. (1996). "Randomized trials in single subjects: the N of 1 study." Psychopharmacol Bull 32(3): 363-367. Dolan, E., A. Stanton, et al. (2005). "Superiority of ambulatory over clinic blood pressure measurement in predicting mortality: the Dublin outcome study." Hypertension 46(1): 156-161.

Crossley G.H et al. (2011). "The Connect (Clinical Evaluation of Remote Notification to Reduce Time to Clinical Decision) Trial. JACC 57(10): 1181-9.

Darkins A, Ryan P, Kobb R, et al. (2008). "Care coordination/home telehealth: the systematic implementation of health informatics, home telehealth, and disease management to support the care of veteran patients with chronic conditions." Telemed. e-Health 14:1118-26.

Ernst, M. E., B. L. Carter, et al. (2006). "Comparative antihypertensive effects of hydrochlorothiazide and chlorthalidone on ambulatory and office blood pressure." Hypertension 47(3): 352-358.

Finkielman, J. D., G. L. Schwartz, et al. (2005). "Lack of agreement between office and ambulatory blood pressure responses to hydrochlorothiazide." Am J Hypertens 18(3): 398-402.

Fujii, T., T. Uzu, et al. (1999). "Circadian rhythm of natriuresis is disturbed in nondipper type of essential hypertension." Am J Kidney Dis 33(1): 29-35.

Gatzka, C. D., H. P. Schobel, et al. (1995). "Normalization of circadian blood pressure profiles after renal transplantation." Transplantation 59(9): 1270-1274.

Guyatt, G., D. Sackett, et al. (1986). "Determining optimal therapy--randomized trials in individual patients." N Engl J Med 314(14): 889-892.

Hansen, T. W., J. Jeppesen, et al. (2005). "Ambulatory blood pressure and mortality: a population-based study." Hypertension 45(4): 499-504.

Holtz B, C. Lauckner (2012). "Diabetes Management via Mobile Phones: A Systematic Review." Telemedicine and e-Health. 18(3): 175-184.

Ingelsson, E., K. Bjorklund-Bodegard, et al. (2006). "Diurnal blood pressure pattern and risk of congestive heart failure." JAMA 295(24): 2859-2866.

Krishna, S., S. Boren, E.A. Balas (2009). "Healthcare via Cell Phones: A Systematic Review." Telemedicine and e-Health 15(3): 231-240.

Laurent, S. (2007). "Day or night blood pressures to predict cardiovascular events?" Lancet 370(9594): 1192-1193.

Lurbe, E., J. Redon, et al. (2002). "Increase in nocturnal blood pressure and progression to microalbuminuria in type 1 diabetes." N Engl J Med 347(11): 797-805.

Mahon, J., A. Laupacis, et al. (1996). "Randomised study of n of 1 trials versus standard practice." BMJ 312(7038): 1069-1074.

McManus, R. et al. (2010). "Telemonitoring and self-management in the control of hypertension (TASMINH2): a randomized controlled trial." Lancet 376: 163-72.

Morgan, T. O. (2002). "Is non-dipping status a predictor of cardiac morbidity?" J Hypertens 20(8): 1469-1471.

Movsowitz, C., S. Mittal (2011). "Remote patient management using implantable devices." J Interv Card Electrophysiolo 31: 81-90.

Muxfeldt, E. S., C. R. Cardoso, et al. (2009). "Prognostic value of nocturnal blood pressure reduction in resistant hypertension." Arch Intern Med 169(9): 874-880.

Pickering, T. G. and W. B. White (2008). "ASH Position Paper: Home and ambulatory blood pressure monitoring. When and how to use self (home) and ambulatory blood pressure monitoring." J Clin Hypertens (Greenwich) 10(11): 850-855.

Sander. G.E., T.D. Giles (2011). "Resistant Hypertension: Concepts and Approach to Management." Current Hypertens Rep 13: 347-355.

Staessen, J. A., L. Bieniaszewski, et al. (1997). "Nocturnal blood pressure fall on ambulatory monitoring in a large international database. The "Ad Hoc' Working Group." Hypertension 29(1 Pt 1): 30-39.

Rothman S. et al. (2007). The Diagnosis of Cardiac Arrhythmias: A Prospective Multi-Center Randomized Study Comparing Mobile Cardiac Outpatient Telemetry Versus Standard Loop Event Monitoring." Journal of Cardiovascular Electrophysiology 18(3): 1-7.

Uzu, T., T. Harada, et al. (2005). "Thiazide diuretics enhance nocturnal blood pressure fall and reduce proteinuria in immunoglobulin A nephropathy treated with angiotensin II modulators." J Hypertens 23(4): 861-865.

Verdecchia, P., G. Schillaci, et al. (1993). "Blunted nocturnal fall in blood pressure in hypertensive women with future cardiovascular morbid events." Circulation 88(3): 986-992.

Wakefield, B.J. (2011). "Home Telehealth in Comorbid Diabetes and Hypertension: A Randomized, Controlled Trial." Telemedicine and e-Health 17(4): 254-261.

Zucker, D. R., C. H. Schmid, et al. (1997). "Combining single patient (N-of-1) trials to estimate population treatment effects and to evaluate individual patient responses to treatment." J Clin Epidemiol 50(4): 401-410.

Appendices

Appendix 1: Study Diagram

Appendix 2: Initial Letters Sent to Patients with High Utilization

Appendix 3: Script for Initial Outreach Phone Call & Screening Form

Appendix 4: Overview of Health Comp Disease Management Program, Sample Letters and Educational Materials

Appendix 5: Baseline Survey

Appendix 6: Terms of Use for Health Comp Portal

Appendix 7: iBG Star Application Disclaimer

Appendix 8: iBG Star End User License Agreement

Appendix 9: Withings Terms of Use

Appendix 10: AliveCor Privacy Notice

Appendix 11: AliveCor Terms of Service

Appendix 12: iBG Star Owner's Guide

Appendix 13: Withings Blood Pressure User Manual

Appendix 14: AliveCor Quick Start Guide

Appendix 15: Healthy Circles iPhone Display

Appendix 16: Text for Study Landing Page (http://wiredforhealth.healthcomp.com)

Appendix 17: iTunes Store Terms and Conditions

Appendix 18: Apple iOS Software License Agreement

Appendix 19: AliveCor Cardiac Report

Appendix 20: E-mail to Scripps Employees

Appendix 21: Revised Recruitment Letter

Appendix 22: Revised Wellness Points Document

Appendix 23: Sticker for Envelope for Recruitment Letter from Health Comp

Appendix 24: 6 Month E-Mail to Patients

Appendix 1: Study Diagram

Wired for Health Study Design

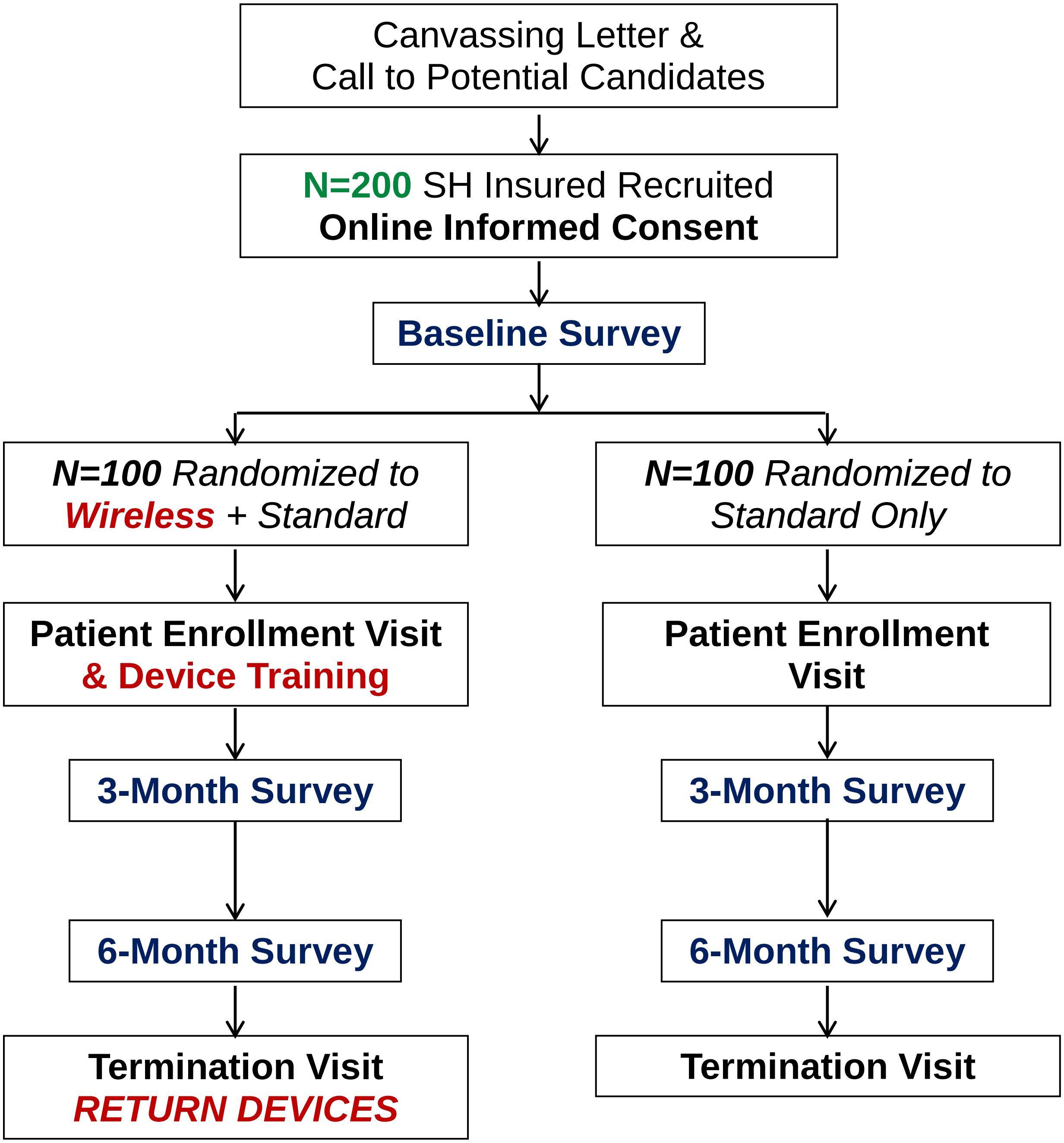

Appendix 2: Initial Letter Sent to Patients with High Utilization

Patient already participating in Disease Management Program:

Scripps Medical Plans

Dear Member,

Thank you for your continued participation in the Disease Management program. We are pleased to be a part of your health care team.

We are sending you this letter to let you know about the new Scripps **Wired for Health Study** and to see if you might have interest in participating. This unique study is focusing on individuals who have diabetes, high blood pressure or a heart arrhythmia. It will test whether individuals who use remote wireless monitoring devices are better able to control their condition and decrease the cost of their health care over a six month period.

Study participants will be randomly assigned to either the control or monitoring group. Both groups will meet with a study staff member, complete health questionnaires and provide information about their current health conditions. The monitoring group will be provided with up to three devices. These devices include a blood pressure monitor, blood sugar monitor and/or heart monitor based on their current health conditions. All three devices will connect to an iPhone that will also be provided to the monitoring group for the duration of the study

The **Scripps Wired for Health Study** is voluntary and will not cost you anything to participate. If you are interested in more information or signing up, please call a Health Comp Nurse at 1-800-442-7247 ext. 2507 or email them at wellness@healthcomp.com If we don't hear from you a Nurse will call you in 2-4 weeks to see if you are interested or have any questions.

We thank you for your time and wish you continued good health.

Sincerely,

Wellness Management
Phone: (800) 442-7247
Email: Wellness@HealthComp.com

Patient not yet participating in Disease Management Program:

Scripps Medical Plans

Dear Member,

As a Nurse with HealthComp, I want to provide you with information on our Disease Management program. This is a free, voluntary program provided to you through your Scripps health plan.

The Disease Management program is designed to partner with you to help you achieve your health goals. You will have access to a nurse that can help answer questions and will check in with you to see how you are feeling. We can also work with you to develop a plan to introduce healthy changes into your lifestyle and provide you with educational materials about your current health conditions.

As part of the Disease Management program you may also be asked to participate in the **Scripps Wired for Health study**. The study is focusing on individuals who have diabetes, high blood pressure or a heart arrhythmia. It will test whether individuals who use wireless remote monitoring devices are better able to control their condition and decrease the cost of their health care over a six month period.

Study participants will be randomly assigned to either the control or monitoring group. Both groups will meet with a study staff member, complete health questionnaires and provide information about their current health conditions. The monitoring group will be provided with up to three devices. These devices include a blood pressure monitor, blood sugar monitor and/or heart/ECG monitor based on current health conditions. All three devices will connect to an iPhone that will also be provided to the monitoring group for the duration of the study.

Both the Disease Management program and **Scripps Wired for Health Study** are voluntary and will not cost you anything to participate. If you are interested in more information or signing up please call a Health Comp Nurse at 1-800-442-7247 ext. 2507 or email them at wellness@healthcomp.com. If we don't hear from you a Health Comp Nurse will call you in 2-4 weeks to see if you are interested or have any questions.

We thank you for your time and wish you continued good health.

Sincerely,
Wellness Management
Phone: (800) 442-7247
Email: Wellness@HealthComp.com

Appendix 3: Script for Initial Outreach Phone Call

Patient Outreach Call: Scripps Wired for Health Study

Not Currently Enrolled in DM Program
My name is ______________________ and I'm calling from Health Comp on behalf of Scripps Health. I want to talk to you about a free and confidential program Scripps is offering designed to help you manage your health goals. The Wellness and Disease Management Program will provide you with access to a nurse that can offer information, help answer questions and give you some reminders on a few things you may not be aware of.

Currently Enrolled in the DM Program
My name is ___________________ and I am calling to say thank you for your participation in the HealthComp Wellness and Disease Management program offered to you by Scripps Health. I hope that you have found it to be helpful and beneficial to your health goals.

The advice given is not meant to replace the care of your medical provider, but rather a complement to that care. We encourage you to establish a relationship with a primary care provider and see them as needed for all preventive and routine care based on your age, gender and condition.

Continue Here to Discuss Study
As part of the Disease Management Program, you may be asked to participate in the Scripps Wired for Health Study. We are enrolling patients who have high blood pressure, diabetes, and/or heart arrhythmias. We're going to test whether individuals who use wireless health devices are better able to control their condition and perhaps use the health care system less often

Are you interested in participating or learning more about either of these programs?

If they express an interest in Scripps study, complete the Screening form.

[If not eligible:
I'm sorry, but you are not eligible to participate in the Scripps study. However, you may still participate in Disease Management Program.]

If eligible:
Do you have 10-15 minutes right now to hear more about the study?

There has been some research which shows that when people take their own health readings at home, they are better able to control their disease, stay healthier and go to the doctor less often. In this study, we are testing wireless devices that you can use at home for diabetes, high blood pressure and cardiac arrhythmias. You will be given an iPhone for use during the study and your recordings will be stored and displayed on the phone.

We will be testing how easy it is to use these devices and whether or how easy it is to fit in your daily schedule.

There are 2 groups in this study and you will be randomly assigned (like flipping a coin) to either the:

- Group A: Disease Management program + devices
- Group B: Disease Management program only

Both groups will meet with a study coordinator 1 time at the beginning of the study and 1 time at the end. These visits will take 30-60 minutes. You will have your blood pressure taken and if you have diabetes, your blood sugar will be taken.

The monitoring group will be given and trained on up to 3 devices: a blood pressure monitor, blood sugar monitor, and a heart monitor, depending on your health. All three devices will connect to an iPhone that will also be provided to the monitoring group for the duration of the study. The iPhone will be programmed so that it can only be used for study purposes and will be returned at the end of the study, along with the study devices. You will be asked to use the devices for a period of six months.

If you are in the device group you will be contacted by phone 7-10 days after your visit with the study coordinator to confirm devices are working properly.

The study will last for 6 months from the time of your visit with the study coordinator. You'll get an e-mail about 3 months after that visit and be asked to complete another survey online. You'll also be asked to complete a final survey at 6 months. The questions will be about your health, how often you go to the doctor, your feelings toward new technology.

As a part of the Disease Management program, the nurses at Health Comp will call you periodically to see how you're doing. We will review with you what if any deficits may be outstanding and we will provide you with some education both verbally and written on your chronic health. If communication with your provider is necessary we are happy to contact them.

The study is totally voluntary and you can quit at any time.

Everyone who is participating in the study is either a Scripps employee or family member. The employee will receive a $20 gift card and 10 Scripps Health Wellness points at the completion of the study. As you may know, if an employee earns 15 Wellness points in a calendar year, they may be eligible for a reduction in health insurance costs.

You may or may not benefit from participating in this study. If you're in the device group, you'll be participating in the Disease Management program as well as be able to see your reading on your iPhone. If you're in the other group, you'll still be able to benefit from Health Comp's Disease Management program.

Do you think you'd like to participate?

I have some questions to ask to confirm that you are eligible.

WFH Screening Contact

Date of Contact: ____________ Time of Contact: __________ AM PM

Name: ___________________________ Member ID: ____

Gender: Male ___ Female ____ Year of Birth: ___________ Age: ________

Patient is: Employee ____ Spouse ____ Dependent ____

Outcome of Call:

- ❑ Interested in both DM program and study. Completed screening form
- ❑ Not interested in either DM program or study (requests no further contact)
  - o Reason: ____
- ❑ Interested in DM but not study
  - o Reason: ____

WFH Screening Form

Do you have any the following?

**High Blood Pressure or Hypertension** ☐ Yes ☐ No	Diagnosed by doctor? ☐ Yes ☐ No	Year diagnosed ________	On medication for this condition? ☐ Yes ☐ No
**Non-Insulin Dependent Diabetes** ☐ Yes ☐ No	Diagnosed by doctor? ☐ Yes ☐ No	Year diagnosed ________	On medication for this condition? ☐ Yes ☐ No
**Insulin Dependent Diabetes** ☐ Yes ☐ No	Diagnosed by doctor? ☐ Yes ☐ No	Year diagnosed ________	On medication for this condition? ☐ Yes ☐ No
**Cardiac Arrhythmia (heart rhythm problems)** ☐ Yes ☐ No	Diagnosed by doctor? ☐ Yes ☐ No	Year diagnosed ________	On medication for this condition? ☐ Yes ☐ No

Inclusion Criteria: In order to be eligible, patient must answer 'Yes' to all:

☐ Yes ☐ N	Are you a Scripps insured employee or family member?
☐ Yes ☐ N	Do you have high blood pressure, diabetes and/or heart arrhythmias?
☐ Yes ☐ N	Do you have computer and internet access, as well as ability to use e-mail and possibly text messaging?
☐ Yes ☐ N	Are you already participating in or willing to join the Health Comp Disease Management program?
☐ Yes ☐ N	Are you 18 years of age or older?

Exclusion Criteria: In order to be eligible, patient must answer 'No' to all:

☐ Yes ☐ No	Do you anticipate any changes over the next 6 months in that you would no longer be covered under Scripps insurance (leaving job, retiring, etc.)?
☐ Yes ☐ No	If you are assigned to the monitoring group, do you have any upcoming surgery, extended trips or any other reason that you may not be able to regularly use the device assigned to you?
☐ Yes ☐ No	Are you related to or sharing a household with anyone participating in this study?
☐ Yes ☐ No	Are you pregnant?

For patients diagnosed with cardiac arrhythmia only:

☐ Yes ☐ No	Do you have an implanted ICD, pacemaker or implanted loop recorder, or expecting to undergo insertion during the next 6 months?

What Scripps facility would be most convenient for you?

- ❑ Clinic location on N. Torrey Pines Road
- ❑ Chula Vista
- ❑ Mercy San Diego

In general, what is the best time of the day for a 1 hour appointment?

- ❑ Morning (9:00-12:00)
- ❑ Afternoon (1:00-5:00)
- ❑ Evening (5:00-7:00)

Do you currently use a smart phone? □ Yes □ No If yes, what kind?

- ☐ iPhone
- ☐ Android (Samsung, Motorola, Kyocera, etc.)
- ☐ Blackberry

If yes, how long have you been using it? ___________.

If no, do you have a cell phone? What kind? _________________

Do you anticipate any problems with carrying 2 phones and learning how to use an iPhone? □ Yes □ No

Have you had any previous experience with using a home health monitoring device? □ Yes □ No
If yes, what device and for how long? ________________ device for _____ months

Based on your diagnosis of [hypertension, NIDDM, IDDM or arrhythmia], are you willing to do home measurement on this schedule? □ Yes □ No

[Circle device and tell patient expected frequency of measurement.]

Device	Frequency of Measurement
**HTN: Withings BP**	2 x per day, 3 days per week, 1st one in a.m.
**NIDDM: iBG Star**	1 x per day (premeal), 3 days per week
**IDDM: iBG Star**	4 x per day (premeal, with 4th reading at bedtime, 2 hours after last meal/dinner), every day
**Arrhythmia: Alive Cor ECG**	if symptomatic

Phone #s: Home: ______________ Cell: _______________

E-mail address You Check Most Frequently: _________________

The first thing that has to happen is you need to sign a consent form for the study. We are doing this on line so you can read it and sign it on your computer. You don't have to print it out. After we're done talking, you will be emailed a link to the Informed Consent form. Please read it carefully. There will be a phone number included so if you have any questions regarding the study, you should call and make sure you get your questions answered before you sign the consent form. You will get instructions on how to electronically sign the form. After you have signed it and clicked "Submit," you will be automatically directed to a health survey to complete on the computer.

After you have signed the Informed Consent form and completed the health survey, you will get a call from one of the study coordinators at Scripps to schedule your initial visit . This visit can be done at a Scripps facility near Green Hospital, Scripps Mercy Hospital or Scripps Chula Vista Hospital. If you decide not to participate before your appointment, we will cancel the appointment and there will be no more contact regarding the study. Which location is most convenient for you? When would be a good day/time for this appointment?

You'll receive a notice by e-mail confirming this appointment and showing you how to change it, if you need to.

Thank you so much for your interest. You will receive an e-mail within 2 days with instructions about reading and signing the consent form.

Thanks again.

Appendix 4

Overview of Health Comp's Disease Management Program,
Sample Letter and Educational Materials

HealthComp
Third Party Administrators
Wellness Management

Overview of Disease Management Program

The goal of the Disease Management program is to assist patients in making health behavior changes. We feel like the best way to promote a change is with "one on one" nurse to patient intervention. We follow the process listed below, but the biggest contributing factor in creating a change is the trust and integrity that is built in this relationship.

An algorithm is used to rank risk indicators associated with the group's population:

1. 3 Major disease states are managed
    - Heart arrhythmia
    - Cardiac
    - Diabetes
2. Claims and pharmacy data are reviewed
3. Outreach Call is made to patient
4. Deficit List is completed
    - Unfulfilled deficits are identified
    - Intervention plan is organized
    - Educational material is sent based on care plan
5. Summary of findings is sent to the patient
6. Repeat this process starting with 3 every 3 to 6 months. The employee can stay in the program indefinably.
7. We may contact the treating provider to advice of those missing deficits and request the provider review the patients chart.

Mailing Address: P.O. Box 45018, Fresno, CA 93718-5018
Phone: (800) 755-7247 Fax: (559) 243-7012

HealthComp
Third Party Administrators
Wellness Management

Dear :

As a Nurse with the HealthComp Wellness Management Program, I want to provide you with information on this voluntary Program, which is a free benefit provided through your health plan.

The goal of the Wellness Management Program is to act as a resource for you and your family. We consist of a Wellness team that includes nurses, pharmacists, and other ancillary staff working on your behalf. We assist you by answering questions, sending health information, reviewing medications, or by acting as a patient advocate for you with your physician. However, we are in no way a replacement to your health care provider, but rather an additional resource for you.

We use national guidelines of care to ensure that you are within acceptable health standards. In light of that, we have noticed there may be an incomplete record of your care, as noted in the following pages. These recommendations are subject to your specific benefits plan and deductibles. ***Only the areas marked with an "X" are relevant to you.*** If we are in error, please contact us to clarify. Because our intent is to ensure that you are receiving the best care possible, we advise that you take this letter with you to your next doctor visit in order to discuss the following recommendations.

If you have any questions or are unsure about a medication, medical procedure, or are simply curious about how to better manage your health, please contact me at the number listed below. I thank you for your time and wish you continued good health.

Sincerely,

Your Wellness Team

Mailing Address: P.O. Box 45018, Fresno, CA 93718-5018
Phone: (800) 755-7247 Fax: (559) 243-7012

HealthComp
Third Party Administrators
Wellness Management

Clinical Recommendations based on National Care Guidelines			
Clinical Recommendations for Diabetes Care			
**Lab tests**		**Pharmacological Management**	
☐	Hemoglobin A1C done at least every six months (blood test to check how your blood sugar levels have been on average over the last 3 months)	☐	Cholesterol lowering medication for those greater than 40 years of age and/or with an LDL level (bad cholesterol) of 135 or greater (medication to decrease risk of cardiovascular disease)
☐	Lipid Profile annually (blood test to screen for high cholesterol and possible coronary artery disease, very common with diabetes)		
☐	Urine Microalbumin annually (urine test to evaluate kidney function)	☐	Angiotensin Converting Enzyme (ACE) Inhibitor or Angiotensin Receptor Blocker (ARB) (medication used to protect kidneys)
☐	Serum Creatinine annually (blood test to evaluate your kidney function)	☐	
☐	Liver Function Tests (LFT's) annually if on certain cholesterol lowering medications (blood test to evaluate liver function)	**Miscellaneous Exams**	
		☐	Semiannual Dental Exam (to evaluate for tooth decay and periodontal disease)
**Vaccines**			
☐	Seasonal Flu Vaccine (to prevent the Flu)	☐	Annual Eye Exam (to evaluate for diabetes related eye disease and risk for blindness)
☐	Pneumococcal Vaccine every 5-10 years (to prevent bacterial pneumonia)	☐	Annual Foot Exam (to evaluate for diabetes related nerve damage and skin integrity)
Clinical Recommendations for Heart Disease Management			
**Lab tests**		**Pharmacologic Management**	
☐	Congestive Heart Failure-Annual Cholesterol Test (blood test to evaluate cholesterol levels)	☐	Congestive Heart Failure-Beta Blocker *and* Vasodilator, or ACE-I/ARB (medication to decrease the workload of the heart)
☐	Congestive Heart Failure-Annual Metabolic Panel (blood test to check for changes in your blood chemistry due to medications)	☐	Coronary Artery Disease or Dyslipidemia-Cholesterol-lowering medication (medication to prevent elevated cholesterol levels)
☐	Coronary Artery Disease or Dyslipidemia-Annual Cholesterol Test (blood test to evaluate cholesterol levels)	☐	Post Heart Attack-on a Cholesterol-lowering medication (to prevent high cholesterol levels)
		☐	Post Heart Attack-on a Beta Blocker (medication to decrease the workload of the heart)
☐	Coronary Artery Disease-Annual Glucose Test (blood test to evaluate risk of diabetes)	☐	Daily Baby Aspirin Men age 45-79 and Women age 55-79 (to prevent heart attack)
☐	Liver Function Tests (LFT's) annually if on certain cholesterol lowering medications (blood test to evaluate liver function)	☐	Atrial Fibrillation-Anticoagulation (medication to prevent strokes)
☐	Atrial Fibrillation-Monthly Protime [blood test to evaluate clotting time related to warfarin (Coumadin) use]	☐	Atrial Fibrillation-on a Beta Blocker or Calcium Channel Blocker (medication to maintain heart rate)
**Vaccines**		**Miscellaneous Exams/Recommendations**	
☐	Seasonal Flu Vaccine (to prevent the Flu)	☐	Annual Cardiac Stress Test (treadmill test to evaluate heart function and disease)
☐	Pneumococcal Vaccine every 5-10 years (to prevent bacterial pneumonia)		

Mailing Address: P.O. Box 45018, Fresno, CA 93718-5018
Phone: (800) 755-7247 Fax: (559) 243-7012

DIABETES

HealthComp®
Third Party Administrators
Wellness Management

Plan for Wellness

Managing your blood sugar

If you have diabetes, you should know the basic steps for managing it so that you stay as healthy as possible. Poorly managed diabetes can lead to many health problems.

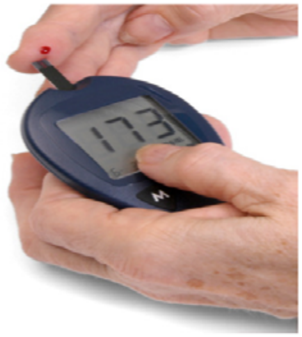

All people with diabetes should know:

- How to recognize and treat low blood sugar (hypoglycemia)
- How to recognize and treat high blood sugar (hyperglycemia)
- Diabetes meal planning
- How to monitor your blood sugar
- What to do when you are sick
- Where to buy diabetes supplies and how to store them
- What checkups you may need

If you take insulin, you should also know:

- How to give yourself insulin
- How to adjust your insulin doses and foods you eat if you exercise

Check Your Blood Sugar Often

Checking your blood sugar levels often and writing down the results will tell you how well you are managing your diabetes. Talk to your doctor and diabetes educator about how often.

Usual times to test your blood sugar are before meals and at bedtime.

- *Other times to check* your blood sugar may be:
- After you eat out, especially if you have eaten foods you do not normally eat
- If you feel sick
- Before and after you exercise
- If you have a lot of stress
- If you eat too much
- If you are taking new medicines
- Keep a record for yourself and your doctor or nurse. This will be a big help if you are having problems managing your diabetes.

Recommended Blood Sugar Targets

For people with type 1 diabetes, the American Diabetes Association recommends the following blood sugar targets. Talk to your doctor and diabetes educator about these goals.

Before meals, your blood sugar should be:

- From 90 - 130 mg/dl for adults
- From 90 - 130 mg/dl for children 13 - 19 years old
- From 90 - 180 mg/dl for children 6 - 12 years old
- From 100 - 180 mg/dl for children under 6 years old

After meals (1 - 2 hours after eating), your blood sugar should be:

- Less than 180 mg/dl for adults

At bedtime, your blood sugar should be:

- From 90 - 150 mg/dl for adults
- From 90 - 150 mg/dl for children 13 - 19 years old
- From 100 - 180 mg/dl for children 6 - 12 years old
- From 110 - 200 mg/dl for children under 6 years old

For people with type 2 diabetes, the American Diabetes Association recommends the following blood sugar targets. Talk to your doctor and diabetes educator about these goals.

Before meals, your blood sugar should be:

- From 70 - 130 mg/dl for adults

After meals (1 to 2 hours after eating), your blood sugar should be:

- Less than 180 mg/dl for adults

HealthComp Wellness Management
P.O. Box 45018
Fresno, CA 93718
800-755-7247
www.healthcomp.com
wellness@healthcomp.com

Content Source: http://www.nlm.nih.gov/medlineplus/ency/patientinstructions/000086.htm

Appendix 5

SCRIPPS WIRED FOR HEALTH STUDY - BASELINE SURVEY

I. Demographics

1. What is your gender?
   a. Male
   b. Female

2. What year were you born? (pull down)

3. What is your ethnicity? (pull down: Caucasian, African-American, Hispanic/Latino, Asian, American Indian/Alaskan Native, Native Hawaiian/Pacific Islander, Other)

4. What is your marital status? (pull down: married, single, divorced, separated)

5. What is the highest level of education you've completed? (pull down: completed 11 or fewer years, graduated from HS or GED completed, graduated from 2-year college, graduated from 4-year college, completed some post-college education, completed Master's degree, completed professional or Ph.D.)

This study is about health care costs, so we would like to better understand your financial situation and the amount of money you have available to spend on your health care.

6. What is your approximate household income? (pull down: under 25k, 25-49k, 50k-99k, 100k-149k, 150k-199k, 200k-249k, 250k-299k, 300k or more)

7. How many people live in your house? (pull down: just me, 1, 2, 3, 4, 5, 6, more than 6)

8. How many people are financially dependent on you (i.e., your number of "dependents")? (pull down: just me, 1, 2, 3, 4, 5, 6, more than 6)

9. Please estimate your approximate household monthly disposable income (i.e., the amount of money you have to spend or save after paying all your bills)? (pull down)

10. What were your approximate out-of-pocket healthcare expenses last year? (pull-down)

II. *Health History*

1. How is your health? (pull down: very good, good, average, poor, very poor)
2. Do you currently use tobacco (i.e., cigarettes, cigars, pipe, chewing tobacco)? (yes/no)
3. If no, have you previously used tobacco? (yes/no)
4. If you are a current or previous user of tobacco, how many times per week do you/did you use it?
5. If you are a current or previous user of tobacco, for how many years have you/did you use it? (pull down)
6. Do you drink alcohol? (yes/no)
7. If no, was there a previous time during which you drank alcohol? (yes/no)
8. If you are a current or previous drinker, how many drinks per week do you/did you have?
9. If you are a current or previous drinker, for how many years have you/did you drink? (pull down)
10. Have you ever been told by a doctor or other health professional that you have high blood pressure or hypertension? (yes, no, not sure)
11. Have you ever been told by a doctor or other health professional that you have diabetes? (yes, no, not sure)
12. Have you ever been told by a doctor or other health professional that you have pre-diabetes? (yes, no, not sure)
13. Have you ever been told by a doctor or other health professional that you have an abnormal heart rhythm? (yes, no, not sure)
14. Please list any and all additional health conditions that you have been told by a doctor or other health professional that you currently have.
15. Please list all of the medications for which you currently have a prescription from a doctor or other health professional.

III. Health Literacy (single item)

How often do you need to have someone help you when you read instructions, pamphlets, or other written material from your doctor or pharmacy? (pull down: Never, Rarely, Sometimes, Often, and Always)

IV. Perceived Health Services Utilization (PERC)

1. Please answer the following:

   a. In the past 6 months, how many times did you visit a physician? Do **not** include visits while in the hospital or to a hospital emergency room. Fill in with “0” or another number. ____times

   b. In the past 6 months, how many times did you go to a **hospital** emergency room? Fill in with “0” or another number. ____times

   c. How many different **times** did you stay in a hospital **overnight** or longer in the past 6 months? Fill in with “0” or another number. ____times

   d. How many total **nights** did you spend in the hospital in the past 6 months? Fill in with “0” or another number. ____nights

V. Health Locus of Control (MHL Form C)

Each item below is a belief statement about your medical condition with which you may agree or disagree. Beside each statement is a scale which ranges from strongly disagree (1) to strongly agree (6). For each item we would like you to circle the number that represents the extent to which you agree or disagree with that statement. The more you agree with a statement, the higher will be the number you circle. The more you disagree with a statement, the lower will be the number you circle. Please make sure that you answer **EVERY ITEM** and that you circle **ONLY ONE** number per item. This is a measure of your personal beliefs; obviously, there are no right or wrong answers.

		**Strongly Disagree**	**Moderately Disagree**	**Slightly Disagree**	**Slightly Agree**	**Moderately Agree**	**Strongly Agree**
1	If my condition worsens, it is my own behavior which determines how soon I will feel better again.	1	2	3	4	5	6
2	As to my condition, what will be will be.	1	2	3	4	5	6
3	If I see my doctor regularly, I am less likely to have problems with my condition.	1	2	3	4	5	6
4	Most things that affect my condition happen to me by chance.	1	2	3	4	5	6
5	Whenever my condition worsens, I should consult a medically trained professional.	1	2	3	4	5	6
6	I am directly responsible for my condition getting better or worse.	1	2	3	4	5	6
7	Other people play a big role in whether my condition improves, stays the same, or gets worse.	1	2	3	4	5	6
8	Whatever goes wrong with my condition is my own fault.	1	2	3	4	5	6
9	Luck plays a big part in determining how my condition improves.	1	2	3	4	5	6
10	In order for my condition to improve, it is up to other people to see that the right things happen.	1	2	3	4	5	6
11	Whatever improvement occurs with my condition is largely a matter of good fortune.	1	2	3	4	5	6
12	The main thing which affects my condition is what I myself do.	1	2	3	4	5	6
13	I deserve the credit when my condition improves and the blame when it gets worse.	1	2	3	4	5	6
14	Following doctor's orders to the letter is the best way to keep my condition from getting any worse.	1	2	3	4	5	6
15	If my condition worsens, it's a matter of fate.	1	2	3	4	5	6
16	If I am lucky, my condition will get better.	1	2	3	4	5	6

17	If my condition takes a turn for the worse, it is because I have not been taking proper care of myself.	1	2	3	4	5	6
18	The type of help I receive from other people determines how soon my condition improves.	1	2	3	4	5	6

VI. Health Self-Efficacy (SE for Managing Chronic Disease 6-Item Scale)

We would like to know how confident you are in doing certain activities. For each of the following questions, please choose the number that corresponds to your confidence that you can do the tasks regularly at the present time.

1. How confident are you that you can keep the fatigue caused by your disease from interfering with the things you want to do?

   not at all | | | | | | | | | | totally
   confident 1 2 3 4 5 6 7 8 9 10 confident

2. How confident are you that you can keep the physical discomfort or pain of your disease from interfering with the things you want to do?

   not at all | | | | | | | | | | totally
   confident 1 2 3 4 5 6 7 8 9 10 confident

3. How confident are you that you can keep the emotional distress caused by your disease from interfering with the things you want to do?

   not at all | | | | | | | | | | totally
   confident 1 2 3 4 5 6 7 8 9 10 confident

4. How confident are you that you can keep any other symptoms or health problems you have from interfering with the things you want to do?

   not at all | | | | | | | | | | totally
   confident 1 2 3 4 5 6 7 8 9 10 confident

5. How confident are you that you can do the different tasks and activities needed to manage your health condition so as to reduce you need to see a doctor?

   not at all | | | | | | | | | | totally
   confident 1 2 3 4 5 6 7 8 9 10 confident

6. How confident are you that you can do things other than just taking medication to reduce how much you illness affects your everyday life?

   not at all | | | | | | | | | | totally
   confident 1 2 3 4 5 6 7 8 9 10 confident

VII. Patient Activation (Me and My Health)

INSTRUCTIONS: For each statement below please mark an X in the box to the right that best describes how much you disagree—agree with the statement as it applies to you personally.

There are no right or wrong answers. Your answers should be what is true to you and not just what you think the doctor wants you to say.

	Strongly Disagree	Disagree	Agree	Strongly Agree
1. When all is said and done, I am the person who is responsible for managing my health condition.				
2. Taking an active role in my own health care is the most important factor in determining my health and ability to function.				
3. I am confident that I can take actions that will help prevent or minimize some symptoms or problems associated with my health condition.				
4. I know what each of my prescribed medications do.				
5. I am confident that I can tell when I need to go get medical care and when I can handle a health problem myself.				
6. I am confident I can tell a doctor concerns I have even when he or she does not ask.				
7. I am confident that I can follow through on medical treatments I need to do at home.				
8. I understand the nature and causes of my health condition(s).				
9. I know the different medical treatment options available for my health condition.				
10. I have been able to maintain the lifestyle changes for my health condition that I have made.				
11. I know how to prevent further problems with my health condition.				
12. I am confident I can figure out solutions when new situations or problems arise with my health conditions.				
13. I am confident that I can maintain lifestyle changes, like diet and exercise, even during times of stress.				

VIII. Health-Related Quality of Life (SF12)

For each of the following questions, please circle the number that best describes your answer.

1. In general, would you say your health is:
_____ Excellent (1)
_____ Very Good (2)
_____ Good (3)
_____ Fair (4)
_____ Poor (5)

The following two questions are about activities you might do during a typical day. Does YOUR HEALTH NOW LIMIT YOU in these activities? If so, how much?

2. MODERATE ACTIVITIES, such as moving a table, pushing a vacuum cleaner, bowling, or playing golf:
_____ Yes, Limited A Lot (1)
_____ Yes, Limited A Little (2)
_____ No, Not Limited At All (3)

3. Climbing SEVERAL flights of stairs:
_____ Yes, Limited A Lot (1)
_____ Yes, Limited A Little (2)
_____ No, Not Limited At All (3)

During the PAST 4 WEEKS have you had any of the following problems with your work or other regular activities AS A RESULT OF YOUR PHYSICAL HEALTH?

4. ACCOMPLISHED LESS than you would like:
_____ Yes (1)
_____ No (2)

5. Were limited in the KIND of work or other activities:
_____ Yes (1)
_____ No (2)

During the PAST 4 WEEKS, were you limited in the kind of work you do or other regular activities AS A RESULT OF ANY EMOTIONAL PROBLEMS (such as feeling depressed or anxious)?

6. ACCOMPLISHED LESS than you would like:
_____ Yes (1)
_____ No (2)

7. Didn't do work or other activities as CAREFULLY as usual:
_____ Yes (1)
_____ No (2)

8. During the PAST 4 WEEKS, how much did PAIN interfere with your normal work (including both work outside the home and housework)?
_____ Not At All (1)
_____ A Little Bit (2)
_____ Moderately (3)
_____ Quite A Bit (4)
_____ Extremely (5)

The next three questions are about how you feel and how things have been DURING THE PAST 4 WEEKS. For each question, please give the one answer that comes closest to the way you have been feeling. How much of the time during the PAST 4 WEEKS –

9. Have you felt calm and peaceful?
_____ All of the Time (1)
_____ Most of the Time (2)
_____ A Good Bit of the Time (3)
_____ Some of the Time (4)
_____ A Little of the Time (5)
_____ None of the Time (6)

10. Did you have a lot of energy?
_____ All of the Time (1)
_____ Most of the Time (2)
_____ A Good Bit of the Time (3)
_____ Some of the Time (4)
_____ A Little of the Time (5)
_____ None of the Time (6)

11. Have you felt downhearted and blue?
_____ All of the Time (1)
_____ Most of the Time (2)
_____ A Good Bit of the Time (3)
_____ Some of the Time (4)
_____ A Little of the Time (5)
_____ None of the Time (6)

12. During the PAST 4 WEEKS, how much of the time has your PHYSICAL HEALTH OR EMOTIONAL PROBLEMS interfered with your social activities (like visiting with friends, relatives, etc.)?
_____ All of the Time (1)
_____ Most of the Time (2)
_____ A Good Bit of the Time (3)
_____ Some of the Time (4)
_____ A Little of the Time (5)
_____ None of the Time (6)

IX. Physician Communication

The purpose of this questionnaire is to obtain your views about your communications with your **physician (the one you see most often) about your medical problems.** Please show how strongly you agree or disagree with these statements by **checking the box that best fits your views.**

		**Strongly Disagree**	**Disagree**	**Slightly Disagree**	**Not Sure**	**Slightly Agree**	**Agree**	**Strongly Agree**
**I DO A GOOD JOB OF:**								
1.	Explaining my medical problem.							
2.	Describing the symptoms of my medical problem(s).							
3.	Answering the doctor's questions thoroughly.							
4.	Answering the doctor's questions honestly.							
5.	Letting the doctor know when I didn't understand something.							
6.	Getting answers to my questions.							
7.	Getting all the information I need.							
**MY PHYSICIAN EXPLAINS THE FOLLOWING TO MY SATISFACTION:**								
1.	Explaining what my medical problems are.							
2.	Explaining the possible causes of my medical problems.							
3.	Explaining what I may be able to do to get better.							
4.	Reviewing or repeating important information.							
5.	Making sure I understand his/her explanations/directions.							
6.	Using language I can understand.							
7.	Encouraging me to ask questions.							
**Please indicate how strongly you agree or disagree with these statements:**								
	Overall, I am satisfied with my physician's communication regarding my medical problems.							

X. Medication Adherence (Morisky 8-Item)

**Question**	**Yes**	**No**
1. Do you sometimes forget to take your medicine?		
2. People sometimes miss taking their medicines for reasons other than forgetting. Thinking over the past 2 weeks, were there any days when you did not take your medicine?		
3. Have you ever cut back or stopped taking your medicine without telling your doctor because you felt worse when you took it?		
4. When you travel or leave home, do you sometimes forget to bring along your medicine?		
5. Did you take all your medicines yesterday?		
6. When you feel like your symptoms are under control, do you sometimes stop taking your medicine?		
7. Taking medicine every day is a real inconvenience for some people. Do you ever feel hassled about sticking to your treatment plan?		

8. How often do you have difficulty remembering to take all your medicine?

__ A. Never/rarely

__ B. Once in a while

__ C. Sometimes

__ D. Usually

__ E. All the time

XI. Exercise (GLTEQ)

1. During a typical 7-Day period (a week), how many times on the average do you do the following kinds of exercise for more than 15 minutes during your free time (write on each line the appropriate number).

	Times Per Week
a) STRENUOUS EXERCISE<br>(HEART BEATS RAPIDLY)<br>(e.g., running, jogging, hockey, football, soccer, squash, basketball, cross country skiing, judo, roller skating, vigorous swimming, vigorous long distance bicycling)	
b) MODERATE EXERCISE<br>(NOT EXHAUSTING)<br>(e.g., fast walking, baseball, tennis, easy bicycling, volleyball, badminton, easy swimming, alpine skiing, popular and folk dancing)	
c) MILD EXERCISE<br>(MINIMAL EFFORT)<br>(e.g., yoga, archery, fishing from river bank, bowling, horseshoes, golf, snow-mobiling, easy walking)	

2. During a typical 7-Day period (a week), in your leisure time, how often do you engage in any regular activity long enough to work up a sweat (heart beats rapidly)?

OFTEN ______ SOMETIMES ______ NEVER/RARELY ______

XII. Use of Innovations

1. Please answer the following questions regarding your thoughts, views, and feelings about **new medical tests, treatments, and devices.**

		strongly disagree	disagree	neutral	agree	strongly agree
1.	If I heard of a new medical test, treatment, or device I would try to find out more about it					
2.	Among my friends, I am usually one of the first to find out about and/or utilize a new medical test, treatment, or device					
3.	In general, I am hesitant to undergo or utilize a new medical test, treatment, or device					

2. Please answer the following questions regarding your thoughts, views, and feelings about **the use of medical devices that can be used at home** to monitor various aspects of your health, including blood pressure, blood sugar, and heart rhythms.

		strongly disagree	disagree	neutral	agree	strongly agree
1.	The use of at-home medical devices for health monitoring is consistent with my approach to my health					
2.	The use of at-home medical devices for health monitoring fits well with my values and goals					
3.	The use of at-home medical devices for health monitoring is hard to understand					
4.	The use of at-home medical devices for health monitoring has many different parts					
5.	The use of at-home medical devices for health monitoring can help me stay healthier compared to other strategies, like seeing my doctor more often					

3. Do you currently own and use a cell phone?

4. If yes, is your phone a Smart Phone?

5. If yes, is your phone an i-Phone?

6. Please answer the following:

To me, new digital technologies like the Smart Phone are:

b.	important	___:___:___:___:___:___:___	unimportant
c.	boring	___:___:___:___:___:___:___	interesting
d.	relevant	___:___:___:___:___:___:___	irrelevant
e.	exciting	___:___:___:___:___:___:___	unexciting
f.	means nothing	___:___:___:___:___:___:___	means a lot to me
g.	appealing	___:___:___:___:___:___:___	unappealing
h.	fascinating	___:___:___:___:___:___:___	mundane
i.	worthless	___:___:___:___:___:___:___	valuable
i.	involving	___:___:___:___:___:___:___	uninvolving
j.	not needed	___:___:___:___:___:___:___	needed

TERMS OF USE

Welcome to https://healthcomp.staging.healthycircles.com (the "**Site**"). The Site is provided by , (the "**Company**") and hosted by MyTeleHealth Solutions LLC, a Delaware limited liability company providing goods and services under the brand "HealthyCircles" ("**HealthyCircles**," together with **Company**, "**We**" or "**Us**" or "**Our**"). These Terms of Use (this "**Agreement**") are made between the Company, HealthyCircles and you or, in the case that you represent and are using the Site on behalf of a company or other entity, such company or other entity ("**You**"). Please carefully read these Terms of Use.

1. USE OF THE SITE.

All use of the Site is subject to the terms of this Agreement. You may access and use the Site solely for lawful purposes. You must reside in the United States to use the Site. You must be 18 years or older to use the Site. We reserve the right at any time and in Our sole discretion to modify, suspend, or discontinue the Site (or any portion thereof) with or without notice.

2. EMERGENCY CARE.

Do not delay in seeking medical assistance if You have a medical emergency. The Site is not intended to provide emergency or other medical services. If You have a medical emergency, call 911 or Your health care provider.

3. NOT MEDICAL ADVICE.

The Site is not intended to constitute the practice of medicine or intended to provide medical advice or diagnosis. The Site is to be used for informational purposes only. You should always contact a health care provider to obtain a diagnosis, to receive information about potential treatments, and to discuss any questions You may have about Your health. You should also contact Your health care provider before taking any new drug, beginning or discontinuing any treatment, changing Your diet, or for any other health care services.

4. PRIVACY STATEMENT.

The privacy statement governing Your use of the Site, the current version of which is available at [Privacy Statement] (the "**Privacy Statement**"), is incorporated in this Agreement by reference. By accepting this Agreement, You expressly consent to the use and disclosure of Your personally identifiable and other information as described in the Privacy Statement. Neither Company nor HealthyCircles is not a “covered entity” under the Health Insurance Portability and Accountability Act of 1996 and the regulations promulgated thereunder. Further, if You provide information to third parties on or through the Site (including, to third party providers of products, software, services or content on the Site), You acknowledge such third parties may not be bound by the Privacy Statement and such third parties may or may not have restrictions on their use of such information. You acknowledge We are not responsible or liable for the use of Your information, including personal or confidential information, by any third party.

5. REGISTRATION.

To access some areas of the Site, You may have to apply for and be approved as a registered user (a "**Registered User**") of the Site. Your approval as a Registered User is at Our discretion. Upon approval as a Registered User, You may be asked to create a password-protected account (an "**Account**"). You agree to keep Your Account information and password confidential. You may not share Your Account information or password with a third party. You agree to notify Us immediately of any actual or suspected unauthorized use of Your Account. You are solely responsible for all activities that occur through Your Account. We will not be responsible for any loss to You caused by Your failure to comply with these obligations. In connection with Your application to become a Registered User, You will be asked to submit certain information about yourself ("**Registration Information**"). You represent and warrant that: (a) all Registration Information You provide is true, accurate, current, and complete; and (b) You will maintain and promptly update the Registration Information to keep it true, accurate, current, and complete. As part of the registration process, You may be assigned or permitted to create a user ID for use in identifying Your Account (a "**User ID**"). You may not: (i) select or use a User ID of another person with the intent to impersonate that person; (ii) use a User ID in which another person has rights without such person's authorization; or (iii) use a User ID that We deem offensive. Failure to comply with the foregoing will constitute a breach of this Agreement, which may result in immediate termination of Your Account.

6. PURCHASES.

You may enroll in certain services provided through the Site that require a payment ("Subscription"). Subscriptions are sold in twenty-eight (28) calendar day blocks of time, as measured from the date the Subscription is initiated ("**Subscription Blocks**"). Subscription fees, if any, will be charged to Your credit card approximately every 28 days. You will prepay for all Subscription Blocks. Subscription cancellations prior to the fourteenth (14th) day of any Subscription Block are fully refunded for that Subscription Block. You will pay all charges incurred by users of Your credit card, debit card, or other payment method used in connection with a purchase from, or any other transaction with HealthyCircles. You acknowledge and agree that We will have no liability for any over-limit charge or other similar charges incurred by You as a result of Our attempts to charge Your credit card or debit card for the services. You will be obligated to pay fees for any services ordered by You even if We are unable to charge Your credit card.

7. TERM AND TERMINATION.

This Agreement will be effective on the date You first use the Site and will continue until terminated. We may terminate this Agreement immediately, for any reason or no reason, with or without notice to You. You may terminate this Agreement upon notice to Us, such termination effective upon receipt of such notice by Us. Upon any termination of this Agreement: (a) all rights granted to You under this Agreement will cease; (b) You must promptly discontinue all access to any part of the Site and the use of any Content downloaded or otherwise obtained from the Site; and (c) HealthyCircles will refund any applicable fees to You for cancelled Subscription Blocks as set forth in Section 6 of this Agreement. Sections 3 (Not Medical Care), 4 (Privacy Statement), 7 (Term and Termination), 8 (Content), 9 (Use of the Content), 11 (Linked Sites), 12 (Postings and Uploads), 14 (Representations and Warranties), 15 (Disclaimer and Limitation of Liability), 16 (Indemnification), 18 (General Terms) and any other terms that by their nature should reasonably be presumed to survive termination or expiration of this Agreement, will survive termination or expiration of this Agreement for any reason.

8. CONTENT.

The Site and the entire contents of the Site, including, but not limited to, text, files, images, graphics, health sessions, health information, illustrations, audio, video, and photographs on or offered through the Site (collectively, "**Content**") are protected by intellectual property rights, including, as applicable and without

limitation, copyrights, trademarks, patents, and other proprietary and intellectual property rights ("**Intellectual Property Rights**").

9. USE OF THE CONTENT.

Your access to or use of the Content is subject to the terms and conditions of this Agreement. You may use the Content only in the form provided on or through the Site solely for Your own informational purposes. The Content is not intended to cover all medical conditions. Please consult Your doctor or other qualified health care professional if You have any questions about a medical condition or any course of treatment. You must otherwise abide by all Intellectual Property Rights, notices, information, or restrictions contained on or in any Content. Unless otherwise noted on the Site, as between You and Company, HealthyCircles, their partners, affiliates, and licensors ("**Affiliates**"), all Content is owned by Company, HealthyCircles, and their Affiliates. Except as stated in this Agreement, You are granted no licenses or rights, whether by implication, estoppel, or otherwise, in or to the Site or Content, or any Intellectual Property Rights therein or related thereto, and You may not modify, reproduce, perform, display, create derivative works from, republish, post, transmit, participate in the transfer or sale of, distribute, or in any way exploit any portion of the Site or Content.

10. MARKS.

Unless otherwise labeled, all trademarks, service marks, logos, photos, images, avatars, banners, page headers, and any other branding elements displayed on this Site (collectively, the "**Marks**") are the property of Company, HealthyCircles and their Affiliates. Except as expressly set forth in this Agreement, You may not display, link to, or otherwise use the Marks.

11. LINKED SITES.

The Site contains links to third-party sites that are not under Our control, and We are not responsible for any content on any linked site. If You access a third-party site from the Site, then You do so at Your own risk. We provide links only as a convenience, and the inclusion of the link does not imply that We accept any responsibility for the content on those third-party sites. We welcome links to the Site. You may establish a link to this Site, provided that the link does not state or imply any sponsorship or endorsement of Your site by Company or HealthyCircles. You may not use on Your site any Content or Marks appearing on the Site in establishing the link. You may not frame, transclude or otherwise incorporate into another site the Content or other materials on the Site.

12. POSTINGS AND UPLOADS.

The Site may include forums, bulletin boards, chat rooms, or other opportunities through which You may provide or upload content of Your own to the Site. You agree not to upload or provide any content that is: (a) libelous, defamatory, obscene, abusive, pornographic, threatening, or an invasion of privacy; (b) an infringement of the Intellectual Property Rights of any third party; (c) illegal in any way or that advocates illegal activity; (d) an advertisement or solicitation of funds, goods, or services; or (e) a software virus or contains any other harmful computer code, files, or programs. We reserve the right not to post or to remove any of Your content which in our sole discretion violates this Section 12 without prior notice. You hereby represent and warrant to Company, HealthyCircles, and their Affiliates that You own all right, title, and interest in and to any content that You provide or upload to the Site, or that You have sufficient rights, whether by implication, estoppel, or otherwise, to grant Us the rights discussed in this Section 12. You will indemnify, defend, and hold harmless Company, HealthyCircles and their Affiliates from any and all third-party claims, losses, liabilities,

damages, fees, expenses, and costs (including attorneys' fees and court costs) that result from a breach or alleged breach of any representation or warranty set forth in Section 12. By providing or uploading any content to the Site, You grant Us a nonexclusive, royalty-free, perpetual, irrevocable, and fully sublicensable right to use, copy, store, reproduce, modify, display, adapt, publish, translate, create derivative works from, distribute, and display such content throughout the world in any form, media, software, or technology of any kind. In addition, You waive all moral rights in the content and warrant that all moral rights applicable to such content have been waived. You also grant Company and HealthyCircles the right to use Your name in connection with the reproduction or distribution of such material.

13. CLAIMS OF INFRINGEMENT.

Just as We require users of the Site to respect the copyrights and other intellectual property rights of Company, HealthyCircles, their Affiliates, and other third parties, We respect the copyrights and other intellectual property rights of users of the Site and other third parties. If You believe in good faith that Your work has been reproduced on the Site without authorization in a way that constitutes intellectual property infringement, You may notify Healthy Circles' designated agents by email or mail to:

MyTeleHealth Solutions, LLCAttn: Legal Department888 Prospect Street, Suite 330La Jolla, California 92037E mail: legal@healthycircles.com

You must provide the following information:

(a)the identity of the infringed work,

(b)the identity of the material that is claimed to be infringing and that is to be removed or access to which is to be disabled, and location of such material;

(c) Your name, address, daytime phone number, and E mail address, if available;

(d)a statement that You have a good-faith belief that the use of the work is not authorized by the owner, his or her agent, or the law;

(e)a statement of the accuracy of the notice and, under penalty of perjury, that You are authorized to act on behalf of the owner; and

(f) Your electronic or physical signature.

14.REPRESENTATIONS AND WARRANTIES.

You hereby represent, warrant, and covenant for the benefit of Company, HealthyCircles and their Affiliates that: (a) You have the legal right and authority to enter into this Agreement, and, if You are accepting this Agreement on behalf of a company or other entity, to bind the company or other entity to the terms of this Agreement; (b) You have the legal right and authority to perform Your obligations under this Agreement and to grant the rights and licenses described in this Agreement and in any applicable additional agreement You enter into in connection with the Site; and (c) all information You provide to Us in connection with this Agreement and Your access to the Site is correct and current.

15. DISCLAIMER AND LIMITATION OF LIABILITY.

15.1 Disclaimer. THE SITE IS PROVIDED "AS IS" and "AS AVAILABLE" WITH NO WARRANTIES WHATSOEVER. WE DO NOT REPRESENT, WARRANT OR ENDORSE THE ACCURACY OR RELIABILITY OF ANY ADVICE, OPINION, STATEMENT, OR OTHER INFORMATION DISPLAYED, DOWNLOADED FROM, OR DISTRIBUTED THROUGH THE SITE. IT IS YOUR RESPONSIBILITY TO EVALUATE THE ACCURACY, COMPLETENESS OR USEFULNESS OF ANY INFORMATION, OPINION, ADVICE OR OTHER CONTENT AVAILABLE THROUGH THE SITE. YOU SHOULD SEEK THE ADVICE OF PROFESSIONALS, AS APPROPRIATE, REGARDING THE EVALUATION OF ANY SUCH SPECIFIC INFORMATION, OPINION, ADVICE OR OTHER CONTENT. YOU AGREE THAT YOUR ACCESS TO THE SITE IS AT YOUR OWN RISK AND THAT YOU ARE SOLELY RESPONSIBLE FOR ANY LIABILITY OR DAMAGE YOU INCUR THROUGH ACCESS TO THE SITE. EXCEPT WHERE THE LAWS AND REGULATIONS OR A PARTICULAR JURISDICTION CONCERNING WARRANTIES CANNOT BE WAIVED OR EXCLUDED BY AGREEMENT, WE EXPRESSLY DISCLAIM ALL WARRANTIES, WHETHER EXPRESS OR IMPLIED, REGARDING THE SITE, INCLUDING, WITHOUT LIMITATION, ALL WARRANTIES OF TITLE, NONINFRINGEMENT, MERCHANTABILITY, AND FITNESS FOR A PARTICULAR PURPOSE. YOU RECOGNIZE THAT THE CURRENT STATE OF TECHNOLOGY DOES NOT ALLOW FOR ERROR-FREE ACCESS TO THE SITE AND INTERRUPTIONS, CRASHES, AND DOWNTIME BEYOND OUR CONTROL MAY OCCUR FROM TIME TO TIME.

15.2 Limitation.IN NO EVENT WILL COMPANY, HEALTHYCIRCLES, OR THEIR AFFILIATES BE LIABLE FOR ANY DIRECT, CONSEQUENTIAL, SPECIAL, INDIRECT, EXEMPLARY, OR PUNITIVE DAMAGES, WHETHER IN CONTRACT, TORT, OR ANY OTHER LEGAL THEORY, IN CONNECTION WITH, AS A RESULT OF OR ARISING OUT OF: (a) YOUR ACCESS TO OR USE OF THE SITE, CONTENT OR SERVICES, (b) YOUR INABILITY TO USE THE SITE, CONTENT OR SERVICES; (c) ANY LOSS OF DATA AND/OR EQUIPMENT FAILURE; (d) THE PROCUREMENT OF SUBSTITUTE GOODS OR SERVICES RESULTING FROM ANY PROBLEMS WITH THE SITE, CONTENT AND/OR SERVICES PURCHASED OR OBTAINED FROM THE SITE, OR TRANSACTIONS ENTERED INTO, THROUGH OR FROM THE SITE; (e) UNAUTHORIZED ACCESS TO OR ALTERATION OF YOUR TRANSMISSIONS OR DATA; (f) STATEMENTS OR CONDUCT OF ANY THIRD PARTY ON THE SITE; (g) ANY DELAY OR FAILURE OF THE SITE ARISING OUT OF CAUSES BEYOND OUR CONTROL; (h) THE USE OF, REFERENCE TO, OR RELIANCE ON, THE CONTENT; (i) ANY THIRD PARTY MATERIALS, INFORMATION, PRODUCTS AND SERVICES CONTAINED ON, OR ACCESSED THROUGH, THE SITE; OR (j) ANY OTHER MATTER RELATING TO THE SITE, EVEN IF WE HAVE BEEN ADVISED OF THE POSSIBILITY OF SUCH DAMAGES AND NOTWITHSTANDING ANY FAILURE OF ESSENTIAL PURPOSE OR ANY LIMITED REMEDY.

In the event You are dissatisfied with, or dispute, these Terms of Use, the Site or the Content, Your sole right and exclusive remedy is to terminate Your use of the Site and Content, even if that right or remedy is deemed to fail of its essential purpose. You confirm We have no other obligation, liability or responsibility to You or any other party.

16. INDEMNIFICATION.

You hereby indemnify, defend, and hold harmless Company, HealthyCircles, and their Affiliates, employees, trustees, members, agents, contractors, assigns, licensees, and successors in interest ("**Indemnified Parties**") from any and all claims, losses, liabilities, damages, fees, expenses, and costs (including attorneys' fees, court costs, damage awards, and settlement amounts) that result from any claim or allegation against any Indemnified Party arising from Your accessing the Site, use of the Content or Your breach of any term of this Agreement. Company or HealthyCircles will provide You with notice of any such claim or allegation, and Company or HealthyCircles will have the right to participate in the defense of any such claim at its expense.

17. NOTICES.

Except as expressly stated otherwise, any notices required or allowed under this Agreement will be provided by You in writing to HealthyCircles by E-mail to the E-mail address of HealthyCircles listed on the Site on the "Contact Us" page and to Company at . With respect to Our notices to You, We may provide notices, amendments or changes to this Agreement by posting them on the Site and You agree to frequently check for such notices, amendments or changes.

18. GENERAL TERMS.

18.1 Third-Party Beneficiaries. Affiliates are intended third-party beneficiaries under this Agreement with the right to enforce the provisions that directly concern Content to which they have rights.

18.2 Nonassignment. You may not assign or transfer any of Your rights hereunder, and any attempt to do so will be null and void.

18.3 Integration. This Agreement sets forth the entire understanding of the parties and supersedes any and all prior oral and written agreements or understandings between the parties regarding the subject matter of this Agreement. This Agreement may not be modified except upon written changes made by HealthyCircles. The waiver by either party of a breach of any provision of this Agreement will not operate or be interpreted as a waiver of any other or subsequent breach.

18.4 Severability. If for any reason any provision of this Agreement is held invalid or unenforceable in whole or in part in any jurisdiction, such provision will, as to such jurisdiction, be ineffective to the extent of such invalidity or unenforceability, without in any manner affecting the validity or enforceability thereof in any other jurisdiction or the remaining provisions hereof in any jurisdiction.

18.5 Jurisdiction and Venue. This Agreement will be governed by the laws of the State of Colorado, without giving effect to any conflict of laws principles. The parties specifically exclude from application to the Agreement the United Nations Convention on Contracts for the International Sale of Goods and the Uniform Computer Information Transactions Act. Regardless of any statute or law to the contrary, any claim or cause of action arising out of or related to Your use of the Site must be filed by You within one (1) year after such claim or cause of action arose or be forever barred.

18.6 Jurisdiction and Venue. This Agreement will be governed by the laws of the State of Colorado, without giving effect to any conflict of laws principles. The parties specifically exclude from application to the Agreement the United Nations Convention on Contracts for the International Sale of Goods and the Uniform Computer Information Transactions Act. You hereby irrevocably and unconditionally consent to the jurisdiction and venue in the state and federal courts sitting in Denver, Colorado. You hereby irrevocably waive the right to a trial by jury. In any such dispute, the prevailing party will be entitled to recover its reasonable attorneys' fees and expenses from the other party.

18.7 Headings and References. All references in this Agreement to Sections, paragraphs, and other subdivisions refer to the Sections, paragraphs, and other subdivisions of this Agreement unless expressly provided otherwise. Titles and headings appearing at the beginning of any subdivision are for convenience only and do not constitute any part of any such subdivision and will be disregarded in construing the language contained in this Agreement. The word "or" is not exclusive. Words in the singular form will be construed to include the plural and words in the plural form will be construed to include the singular, unless the context otherwise requires.

18.8 Modifications. We reserve the right, at any time and without notice, to add to, change, update, or modify the Site and these Terms of Use, simply by posting such addition, change, update, or modification on the Site. Any such addition, change, update, or modification will be effective immediately upon posting on the Site.

iBGStar® Wired for Health **Application Disclaimer**

Without limiting the generality of Sections d., e. and f. of the Download License:

The iBGStar® Wired for Health application is designed to help User exchange blood glucose readings with healthcare professionals. However, Sanofi has no control over, and makes no representations or warranties, express or implied, regarding User's or User's healthcare provider(s)' use of the iBGStar® Wired for Health application or the use or interpretation of any information stored on, generated by or received through the iBGStar® Wired for Health application. User is solely responsible for communicating any blood-glucose and/or diabetes-related information to (and receiving feedback from) User's healthcare provider(s). The iBGStar® Wired for Health application does not provide Sanofi or its licensors access to data that can identify the User.

User is solely responsible for reviewing and evaluating the accuracy and relevance of any information stored on, generated by or received through the iBGStar® Wired for Health application. Sanofi cannot and does not guarantee said accuracy. THE iBGStar® Wired for Health APPLICATION AND ANY INFORMATION STORED ON, GENERATED BY OR RECEIVED THROUGH THE iBGStar® Wired for Health APPLICATION ARE NOT INTENDED TO BE A SUBSTITUTE FOR PROFESSIONAL MEDICAL ADVICE, DIAGNOSIS, OR TREATMENT. ALWAYS SEEK THE ADVICE OF A PHYSICIAN OR OTHER QUALIFIED HEALTHCARE PROVIDER WITH ANY QUESTIONS REGARDING A MEDICAL CONDITION. NEVER DISREGARD PROFESSIONAL MEDICAL ADVICE OR DELAY IN SEEKING IT BECAUSE OF SOMETHING READ OR LEARNED THROUGH THE USE OF THE iBGStar® Wired for Health APPLICATION OR ANY INFORMATION STORED ON, GENERATED BY OR RECEIVED THROUGH THE iBGStar® Wired for Health APPLICATION.

User acknowledges that Sanofi or its licensors, in their sole discretion, may from time to time make modifications to the iBGStar® Wired for Health application. Such modifications may require corresponding changes to be made in the User's systems. Changes or failure to make timely changes by User may sever or affect User's access to the iBGStar® Wired for Health application. Neither Sanofi nor its licensors shall be responsible for such effects, and neither Sanofi nor its licensors shall have any obligation to furnish any maintenance and support services with respect to the iBGStar® Wired for Health application.

The iBGStar® Wired for Health application is intended for educational and scientific research purposes only. This application is not intended to offer or replace professional medical advice. Please speak to your healthcare provider if you have any health concerns or questions.

Health Disclaimer:
The data communication capabilities provided by the iBGStar® Wired for Health application is provided for general education and scientific research purposes only. Persons using the data within the iBGStar® Wired for Health application for medical purposes, should not rely solely on the accuracy of the data herein.
Please consult your healthcare provider prior to making any lifestyle or treatment changes. Your healthcare professional is the single best source of information regarding your health. Please consult your healthcare professional if you have any questions about your health or treatment.
You are encouraged to report negative side effects of prescription drugs to the FDA.
Visit www.fda/gov/medwatch or call 1-800-FDA-1088.

Any and all liability arising directly or indirectly from the use of the iBGStar® Wired for Health application is hereby disclaimed. The iBGStar® Wired for Health application is provided "as is" and without any warranty express or implied. All direct, indirect, special, incidental, consequential or punitive damages arising from any use of the iBGStar® Wired for Health application or data contained herein are disclaimed and excluded.

iBGStar® Wired for Health End User License Agreement

The iBGStar® and the Wired for Health application and database used for the WFH clinical study under the supervision of Scripps Health, a California based company ("WFH Study"), are protected by copyright and may not be reproduced or used without the express written permission of sanofi-aventis U.S. LLC ("Sanofi US"). You may use the iBGStar® Wired for Health application solely on compatible iPhone computing devices provided in the context of the WFH Study after the appropriate training session. The iBGStar® Wired for Health application can only be used by individuals enrolled in the WFH Study. Making copies of the software or database, or enabling others to use your registration code(s) or serial number(s), if any, is strictly prohibited. It is also prohibited to give or transfer copies to any person who is not participating to the WFH Study; to install the software on other mobile devices; or to duplicate the software by any other means including electronic transmission. The software in its entirety is protected by copyright laws. The software also contains trade secrets and you may not decompile, reverse engineer, disassemble or otherwise reduce the software to human-perceivable form or disable any functionality which limits the use of the software. You may not modify, adapt, translate, rent or sublicense (including offering the software to third parties on an application service provider or time-sharing basis), assign, loan, resell for profit, or distribute the software, disks or related materials or create derivative works based upon the software or any part thereof, without the express written permission of Sanofi US.

Supplementary End User License Agreement

This Supplementary End User License Agreement ("Supplementary License") is a binding legal agreement between you ("you" or "User") and sanofi-aventis U.S. LLC. ("Sanofi US") regarding your use of the iBGStar® Wired for Health application (the "iBGStar® Wired for Health app" or the "software"). This Supplementary License is intended to supplement the terms of the "Licensed Application End User License Agreement" to which User agreed when downloading the iBGStar® Wired for Health application from the Apple Inc. App Store (the "Download License"). Accordingly, User acknowledges and agrees that (1) the iBGStar® Wired for Health application is the "Licensed Application" as defined in the Download License, (2) User continues to be bound by all the terms of the Download License as well as the supplementary terms in this Supplementary License, and (3) in the event of a conflict between the terms of the Download License and this Supplementary License, the terms of the Download License will control.

Subject to the terms of the Download License as supplemented by this Supplementary License, User is granted a non-transferable, non-exclusive and free license for the use of the iBGStar® Wired for Health application on the iPhone, that User controls as permitted by the Download License as supplemented by this Supplementary License including, but not limited to, the Usage Rules set forth in the App Store Terms of Services referenced in the Download License. You may use the iBGStar® Wired for Health application for personal use. The iBGStar® Wired for Health application has been developed by Sanofi or its licensors and is protected by copyright and may not be reproduced or used without the expressed written permission of Sanofi US. Making additional copies of the software, or enabling others to use your registration code(s) or serial number(s), if any, is strictly prohibited. It is also prohibited to duplicate the software by any other means including electronic transmission. The software also contains trade secrets and you may not decompile, reverse engineer, disassemble or otherwise reduce the software to human-perceivable form or disable any functionality which limits the use of the software. You may not modify, adapt, translate, rent or sublicense (including offering the software to third parties on an application service provider or time-sharing basis), assign, loan, resell for profit, or distribute the software, disks or related materials or create derivative works based upon the software or any part thereof, without the express written permission of Sanofi US.

iBGStar® is a Registered Trademark owned by Sanofi. You acknowledge that AgaMatrix is an intended third-party beneficiary of the Download License as supplemented by this Supplementary License.

Privacy Policy

In the context of the WFH Study, Sanofi is not involved in the storage, management or analyze of the users' medical data transmitted by the application. Sanofi US will not have access to any personal information. Sanofi US encourages the persons involved in the WFH clinical study to refer to the data privacy provisions of the study enrollment documentation (HIPAA Authorization).

Breach of Security
User acknowledges that Sanofi or its licensors, in their sole discretion, have taken necessary measures to ensure the privacy, integrity and security of the data entered by the user when transmitting that data between the user's device(s) such as iPhone or a web browser and the servers that will host/store the data. Despite these security and privacy measures, it is possible that there can be a breach in the data security resulting from non-malicious actions of Sanofi or its licensors and/or malicious actions of external parties. Neither Sanofi nor its licensors shall be responsible for such effects, and neither Sanofi nor its licensors shall have any obligation to furnish any maintenance and support services with respect to the iBGStar® Wired for Health application.

Withings Terms of Use of Services

Last update: 01/06/2011
Welcome to the Withings site: www.withings.com is a Site offered by Withings, a simplified stock company (SAS) with 2,551,824 euros in capital, whose registered office is located at 20 rue Rouget de Lisle 92130 ISSY LES MOULINEAUX, registered in the Nanterre Companies and Trade Registry under number 504 787 565.
Please read these Terms of Use carefully; print them up and keep a copy.
Your personal data are protected. The confidentiality of your personal information is essential; we are determined to protect it. Please read the Privacy Rules page for more details.

Definition

The following words and expressions beginning with a capital letter will have the following meanings:
The **"Applications"**: are the digital contents that Withings offers to the User for consultation or download. This software has been developed by Withings. These Applications support the Service;
The **"Account"**: is a strictly personal space created by the User when registering on the Site, enabling him to access the Service;
The **"Interface"**: is a remote display making it possible to utilise all of the Product's functions;
The **"Smartphone"**: is a latest-generation intelligent mobile telephone;
The **"Product"**: is the **"Body Scale"** online object, the **"Blood Pressure Monitor"** online object or the **"Babyphone"** online object;
The **"Service Provider"** or **"Withings"** or **"we/us"**: is Withings, the company that provides the Service;
The **"Service"**: is (i) the platform allowing access to the Product interface via certain smartphones, or (ii) the platform allowing access to the Product interface via the Site, or (iii) the platform making it possible to obtain software to be integrated into the Product, or (iv) the platform allowing installation of the Product from a computer, or (v) the platform allowing access to the Site;
The **"Site"**: is the Web site http://www.withings.com;
The **"User"** or **"you"**: is the person registered on the Site who uses the Service.

Purpose

These Terms of Service are Intended to define the contractual relationship entre le Service Provider and the User.

Acceptance

In order to access the Service, the User must register on the Site by creating an Account. Every registration on the Site assumes prior and unreserved consultation and acceptance by the visitor of all of the present Terms of Use. If the User does not wish to accept these Terms of Use, he must refrain from using the Services.

Access to the Service

Unless otherwise established, the Services are accessible only to corporate bodies or adults.
To access the Service, the User must create – from the Site – an Account protected by a password. The User undertakes to provide true, accurate and complete information and to update it regularly.
The registered User must thereafter identify himself in order to access the Service.
To be able to utilise the Service, the User must have a computer connected to the internet, or a smartphone, also connected to the internet. These costs are borne exclusively by the User.
Withings declines all liability:

- in the event of interruption, bugs or other events rendering the Service unfit for use;
- if the User's computer or smartphone operating system is incompatible with the Service;
- and more generally for any direct or indirect damages, whatever their cause or nature, resulting from use of the Service or the Site.

The Service Provider reserves the right, entirely at its discretion, to temporarily and indefinitely suspend or refuse access to the Service by the User if the latter does not comply with these Terms of Use, or for any other reason. In the case of suspension of the Service, even temporary, these Terms of Use remain applicable.

Account Security

The User is solely responsible for keeping his password confidential and for the actions performed on his Account.
The User must not reveal to anyone information relating to his Account, or use a third party's Account.

Service Content

The Service enables the User:

- to access the Product Interface via certain smartphones;
- to access the Product Interface via the Site;
- to obtain Software to integrate into the Product;
- to obtain software enabling installation of the Product from a computer;
- to access the Site, and, in particular: the showcase, the store, the community spaces, User support, etc.

The User acknowledges that use of the Service or the Products may require the use of other products or hardware or software, and that the User is responsible for that hardware and software.
THE SERVICES AND PRODUCTS TO WHICH THE USER WILL HAVE ACCESS:

- MUST BE USED SOLELY FOR PERSONAL, NON-COMMERCIAL PURPOSES;
- MUST NOT BE SUBJECTED TO CASES OF CRITICAL USE. DETERMINATION OF CRITICALITY IS LEFT TO THE SERVICE PROVIDER'S JUDGMENT.

Changes to the Service or the Product

At its convenience and without prior notice, the Service Provider may modify, add or delete functions of the Service or the Product.

Community services

The User is responsible for information, messages and in general any content distributed via

the community Services, such as the Withings forum or blog.
The User accepts that Withings may eliminate, in whole or in part, any content that it distributes through the interactive services if this content is likely to infringe current laws or regulations or public decency.

Intellectual Property Rights

All texts, photos, logos, trademarks and other elements reproduced on the Site are reserved and protected by copyright, for the entire world.
The User, or a third party, is prohibited from reproducing, representing, distributing, marketing, modifying, utilising or assigning all or part of any of the elements reproduced on the site without the Service Provider's prior consent.
Any unlawful use of all or part of the Site may give rise to criminal prosecution.

Client's Obligations

The User undertakes not to engage in any of the following prohibited activities:

- attempting to use or access the Service via an Account to which he has no right;
- modifying the software supplied, where appropriate, by the Service Provider in order to access the Service, and not to use modified versions of this software and in general not to access it illegally and if not expressly authorised by the Service Provider;
- using the Service for purposes contrary to any current regulations, and not to infringe third-party rights;
- using the Service so as to cause deterioration, interruption or lower performance or to damage its functioning;
- undertaking actions that may, in the Service Provider's judgment, cause an unreasonable or disproportionate increase in the load on our infrastructure;
- interfering with the proper functioning of the Service;
- doing reverse-engineering, decompiling, disassembling or attempting to discover the source code of the Applications.

Changes to the Service's Terms of Use

The Service Provider reserves the right to modify all or part of the Service's Terms of Use without notice.
Use of the Service by the User constitutes full and complete acceptance of any changes made to the present Terms of Use.

Guarantees

The User expressly acknowledges and accepts that the Service Provider:

- does not guarantee that the Services will function without interruption or error;
- cannot guarantee the complete accuracy of the information made available by the Service.

Liability

The User expressly acknowledges and accepts that the Service Provider:

- may temporarily or occasionally interrupt the Service in order to perform maintenance or update operations, without this in any way making the Service Provider liable;

- may in no case be held liable for any damage whatsoever, whether caused directly or indirectly by its services;
- may in no case be held liable for damage to and losses of data suffered by the User. It is therefore up to the User to take all appropriate measures to protect his own data or software from the contamination of his hardware by any viruses circulating on the Internet;
- may in no case be held liable for damages and injury resulting from violation by Users of the rules applying to the community Services (see article 8).

Applicable law

These Terms of Use are governed by French law.
Any dispute resulting from these Terms will fall within the exclusive jurisdiction of the competent French courts.

4/1/13

AliveCor Privacy Notice

www.alivecor.com/privacy 1/2
Date of last revision: July 19, 2012

We at AliveCor, Inc. ("AliveCor") respect your concerns about privacy. This Privacy Notice describes the types of personal information we collect through our mobile app (the "App") and alivecor.com and alivecorvet.com (the "Sites"), how we use the information, with whom we share it and the choices available to users of our App and Sites regarding our use of the information. We also describe the measures we take to protect the security of the information and how users can contact us about our privacy practices.

This Privacy Policy is organized according to the sections below:

Information We Collect
How We Use the Information We Collect
Information We Share
Your Rights And Choices
Data Transfers
How We Protect Personal Information
Links To Other Websites and Applications
Updates To Our Privacy Notice
How To Contact Us

Information We Collect

The types of personal information that users of our Sites and App may submit include:
Contact information (such as name, postal address, email address, and mobile or other telephone number) of individuals such as medical professionals, human patients and/or their parents or guardians, owners of veterinary patients and other visitors;
username (where applicable) and password;
human patient demographics such as date of birth and gender;
human patient medical data such as medications, ailments; and
payment information (such as payment card number, expiration date, delivery address, and billing address).
Other personal information collected by the App includes human electrocardiography ("ECG") data, including the ECG measurement itself, average heart rate and the location on the body where the ECG recording was taken (e.g., hand or chest).
When you visit our Sites or open our emails, we may collect certain information by automated means, such as cookies, web beacons and web server logs. The information we collect in this manner includes IP address, browser characteristics, device characteristics, operating system version, language preferences, referring URLs, information on actions taken on our Sites, and dates and times of website visits. A "cookie" is a file that websites send to a visitor's computer or other Internetconnected device to uniquely identify the visitor's browser or to store information or settings in the browser. A "web beacon" also known as an Internet tag, pixel tag or clear GIF, links web pages to web servers and their cookies and may be used to transmit information collected through cookies back to a web server. Through these automated collection methods, we obtain "clickstream data," which is a log of the links and other content on which a visitor clicks while browsing a website. As the visitor clicks through the website, a record of the action may be collected and stored. We may link certain data elements we have collected through automated means, such as your browser information, with other information

we have obtained about you to let us know, for example, whether you have opened an email we sent to you. Your browser may tell you how to be
notified when you receive certain types of cookies or how to restrict or disable certain types of cookies. Please note, however, that without cookies you
may not be able to use all of the features of our Sites.
In addition, our App collects information such as the mobile device's phone model and operating system version, as well as the phone's location (with
your consent), local time and time zone.

How We Use The Information We Collect

We may use the information described above to:
Create and manage your account;
provide products and services to you;
deliver and manage customer support;
process your payment;
send you promotional materials or other communications;
communicate with you about, and administer your participation in, special events, programs, offers, surveys and market research;
respond to your inquiries;
perform data analyses (including anonymization and aggregation of personal information);
operate, evaluate and improve our business (including developing new products and services; enhancing and improving our services; managing our
communications; analyzing our products; and performing accounting, auditing and other internal functions);
protect against, identify and prevent fraud and other unlawful activity, claims and other liabilities; and
comply with and enforce applicable legal requirements, relevant industry standards and our policies.
We also may use the information in other ways for which we provide specific notice at the time of collection.
In addition, we use information collected through cookies, web beacons, web server logs and other automated means for purposes such as (i)

customizing our users' visits to our websites, (ii) delivering content tailored to our users' interests and the manner in which our users browse our
websites, and (iii) managing our websites and other aspects of our business. To the extent required by applicable law, we will obtain your consent before
collecting information by automated means using cookies or similar automated means.
We may use thirdparty
web analytics services on our Sites, such as those of Google Analytics. The analytics providers that administer these services use
technologies such as cookies, web server logs and web beacons to help us analyze how visitors use the Sites and improve the overall experience of the
Sites. The information collected through these means (including IP address) is disclosed to these analytics providers, who use the information to
evaluate use of the Sites. To learn more about Google Analytics and how to opt out, please
visit http://www.google.com/analytics/learn/privacy.html.

Information We Share

We do not sell or otherwise disclose personal information we collect about you, except as described in this Privacy Notice. With consent, we may share
information relating to patients (as well as veterinary patients' owners and human patients' parents or guardians) with the patients' medical
professionals, whose use, disclosure and other handling of this information is subject to their own privacy policies and practices and not this Privacy

Notice. We may share your personal information among our subsidiaries and affiliates for the purposes described in this Privacy Notice. We also may share personal information with service providers who perform services on our behalf based on our instructions. We do not authorize these service providers to use or disclose the information except as necessary to perform services on our behalf or comply with legal requirements. Examples of these service providers include entities that analyze ECG data to diagnose cardiac conditions, process credit card payments, fulfill orders and provide web hosting services.

In addition, we may disclose information about you (i) if we are required to do so by law or legal process, (ii) to law enforcement authorities or other government officials, and (iii) when we believe disclosure is necessary or appropriate to prevent physical harm or financial loss, or in connection with an investigation of suspected or actual fraudulent or illegal activity.

We also reserve the right to transfer personal information we have about you in the event we sell or transfer all or a portion of our business or assets (including in the event of a reorganization, dissolution or liquidation). Should such a sale or transfer occur, we will use reasonable efforts to direct the transferee to use personal information you have provided to us in a manner that is consistent with our Privacy Notice. Following such a sale or transfer, you may contact the entity to which we transferred your personal information with any inquiries concerning the processing of that information.

Your Rights and Choices

We offer you certain choices in connection with the personal information we collect from you, such as how we use the information and how we communicate with you. To update your preferences, ask us to remove your information from our mailing lists or submit a request, please contact us as indicated in the "How to Contact Us" section of this Privacy Notice. Users with online accounts may be able to update or delete certain personal information on our Sites. To the extent provided by the law of your jurisdiction, you may request access to the personal information we maintain about you or request that we correct, amend, delete or block the information by contacting us as indicated below. Where provided by law, you may withdraw any consent you previously provided to us or object at any time on legitimate grounds to the processing of your personal information, and we will apply your preferences going forward.

Data Transfers

We may transfer the personal information we collect about you to countries other than the country in which the information originally was collected. Those countries may not have the same data protection laws as the country in which you initially provided the information. When we transfer your information to other countries, we will protect that information as described in this Privacy Notice.

How We Protect Personal Information

We maintain administrative, technical and physical safeguards designed to protect the personal information you provide against accidental, unlawful or unauthorized destruction, loss, alteration, access, disclosure or use.

Links To Other Websites and Applications

Our Sites and App may provide links to other websites and applications for your convenience and information. These websites and applications may operate independently from us. Linked sites and applications may have their own privacy notices or policies, which we strongly suggest you review. To the extent any linked websites or applications are not owned or controlled by us, we are not responsible for the sites' or applications' content, any use of

the sites or applications, or the privacy practices of the sites or applications.

Updates To Our Privacy Notice

This Privacy Notice may be updated periodically and without prior notice to you to reflect changes in our personal information practices. We will post a
prominent notice on our Sites to notify you of any significant changes to our Privacy Notice and indicate at the top of the notice when it was most
recently updated.

How To Contact Us

If you have any questions or comments about this Privacy Notice, or if you would like us to update information we have about you or your preferences,
please contact us by email at privacy@alivecor.com. You also may write to: 30 Maiden Lane, 6th Floor, San Francisco, CA 94108.

ALIVECOR, INC. TERMS OF SERVICE

THE AliveECG MOBILE APPLICATION AND WEB SERVICE (hereafter, the "Application") IS AVAILABLE FOR PRESCRIPTION USE ONLY IN THE UNITED STATES OF AMERICA (USA) AND TO USERS IN THE MEMBER STATES OF THE EUROPEAN UNION. THE APPLICATION IS INTENDED TO ONLY RECORD, DISPLAY, STORE AND TRANSMIT ELECTROCARDIOGRAMS (ECG Data). THE USER OF THE APPLICATION IS RESPONSIBLE FOR THE ECG DATA RECORDED AND STORED BY THE APPLICATION. THE APPLICATION IS NOT INTENDED TO DIAGNOSE ANY HEART CONDITION OR ALERT HEALTHCARE PROFESSIONALS OR PATIENTS TO POTENTIALLY SERIOUS HEART CONDITIONS OR ABNORMAL ARRHYTHMIAS. ALIVECOR, INC. (AliveCor) DOES NOT REVIEW, MONITOR, EVALUATE OR ANALYZE ANY INFORMATION GENERATED FROM THE APPLICATION. By clicking the "I AGREE" button that follows these Terms and Conditions or by otherwise accessing or using the Application, you ("You") understand and agree to the above description of the Application and agree as follows:

1. REGISTRATION; TERM OF REGISTRATION

1.1. Registration through the Application

You must be a registered user with AliveCor, Inc. ("AliveCor") in order to use the Application. You may register through the Application or through the AliveCor website located at http://www.alivecor.com ("Website"). By submitting the information requested in the Application's online registration form, You may access and use the Application and Website to view certain data pertaining to You as made available by AliveCor. You may not access or use the Application or Website for any other purpose.

1.2. Term of Your Registration

The term of Your registration will commence as of the date You complete Your online registration form ("Registration Date") and, unless earlier terminated in accordance with these Terms and Conditions, will continue in perpetuity ("Term"). Notwithstanding the foregoing, Your registration may automatically expire following any period of inactivity associated with Your account in excess of twelve (12) consecutive months.

1.3. Notices

All notices from AliveCor intended for receipt by You shall be deemed delivered and effective when sent to the email address provided by You during the registration process or when posted to and made available to You on Application or Website (in either case, "Notice"). If You change the email address provided in connection with Your registration to access and use the Application, You must update Your address in accordance with the procedures set forth on the Website.

2. MODIFICATIONS TO THE APPLICATION / TERMS AND CONDITIONS

2.1. Modifications to the Application / Terms and Conditions

You acknowledge and agree that AliveCor may, in its sole discretion, modify the Application and these Terms and Conditions (including any instructions, policies or guidelines referenced herein) at any time and in any manner. Upon modification of these Terms and Conditions, AliveCor will notify You for Your consent. All such modifications shall be binding upon You when made. You may not amend or modify the Application, Website or these Terms and Conditions under any circumstances.

3. 3. REPRESENTATIONS, COVENANTS AND WARRANTIES

3.1. Representations, Covenants and Warranties of Registrant

You represent, covenant and warrant to AliveCor that (1) You are a licensed healthcare professional in the USA, or You have been prescribed the Application by a licensed healthcare professional in the USA, or you are a resident of a Member State in the European Union; (2) will use the Application as intended; (3) these Terms and Conditions have been executed and delivered by You and constitute a valid and binding agreement with You, enforceable against You in accordance with their terms; (4) You will not access or use the Application or Website except as expressly permitted by these Terms and Conditions and any additional instructions, guidelines or policies issued by AliveCor, including those posted in the Application or on the Website; (5) You will access and use the Application and Website in full compliance with applicable Law (as defined in Section 4.1); and (6) all of the information, data and other materials provided by You in support of Your online registration are accurate and truthful in all respects.

3.2. Disclaimer

EXCEPT AS OTHERWISE EXPRESSLY PROVIDED IN THESE TERMS AND CONDITIONS, ALIVECOR MAKES NO REPRESENTATIONS, COVENANTS OR WARRANTIES AND OFFERS NO OTHER CONDITIONS, EXPRESS OR IMPLIED, REGARDING ANY MATTER, INCLUDING (1) THE MERCHANTABILITY, SUITABILITY, FITNESS FOR A PARTICULAR USE OR PURPOSE, NON-INFRINGEMENT OR RESULTS TO BE DERIVED FROM THE USE OF THE APPLICATION, WEBSITE OR ANY DATA SERVICE, SOFTWARE, HARDWARE, DELIVERABLE, WORK PRODUCT OR OTHER MATERIALS RELATED TO THE APPLICATION OR WEBSITE, OR THE AVAILABILITY OF ANY OF THE FOREGOING; OR (2) WHETHER THE INFORMATION AVAILABLE ON OR TRANSMITTED BY THE APPLICATION OR WEBSITE IS TRUE, COMPLETE OR ACCURATE. YOU SPECIFICALLY ACKNOWLEDGE AND AGREE THAT ALIVECOR IS NOT RESPONSIBLE FOR ANY HEALTHCARE OR RELATED DECISIONS MADE BY YOU OR YOUR HEALTHCARE PROFESSIONAL BASED UPON DATA COLLECTED, TRANSMITTED OR DISPLAYED BY OR ON THE APPLICATION OR WEBSITE, WHETHER SUCH DATA IS ACCURATE OR INACCURATE. FURTHER, ALIVECOR DOES NOT REPRESENT, COVENANT OR WARRANT THAT ACCESS TO OR SERVICES PROVIDED BY THE APPLICATION OR WEBSITE WILL BE UNINTERRUPTED OR ERROR-FREE. YOU ACKNOWLEDGE AND AGREE THAT THERE ARE RISKS INHERENT TO TRANSMITTING INFORMATION OVER AND STORING INFORMATION ON THE INTERNET AND THAT ALIVECOR IS NOT RESPONSIBLE FOR ANY LOSSES OF YOUR DATA, CONFIDENTIALITY OR PRIVACY IN CONNECTION THEREWITH.

4. COMPLIANCE

4.1. Generally

You shall use the Application and Website in strict compliance with (1) these Terms and Conditions; (2) any additional instructions, guidelines or policies issued by AliveCor, including those posted within the Application or on the Website; and (3) all applicable laws, rules and regulations (collectively, "Laws").

5. CONTRACTORS

5.1. Use of Contractors

AliveCor may engage any third party (including AliveCor's affiliates) to perform, or support the performance of, all or any portion of the Application or the Website. Any engagement

with any third party will be conducted in accordance with the AliveCor Privacy Notice described in Section 7.1.

6. INTELLECTUAL PROPERTY RIGHTS; RESTRICTIONS ON USE

6.1. Ownership of the Application and Related Data

As between AliveCor and You (collectively, "Parties"), AliveCor owns and will retain ownership of all rights, title and interest in and to the Application and the Website, including (1) all content displayed on the Application or the Website, to the extent such content is developed by AliveCor or its third party licensors, including any derivative works of the Application or Website and any such content; (2) any data presented on or by the Application or Website or otherwise stored by AliveCor and its third party providers, except for personal information that You submit to AliveCor during the registration process or through Your use of the online forms on the Website or Application (which shall be owned by You); and (3) any intellectual property or other proprietary rights comprising any of the foregoing (collectively, "AliveCor IP"). Neither these Terms and Conditions nor any disclosure made hereunder grant any license to You under any AliveCor IP.

6.2. Use of the Application

You may use the Application and the Website, including any data presented on or by the Application or Website for You, or otherwise hosted or stored by AliveCor for You, only on Your own behalf and for lawful and appropriate purposes. You shall not use the Application, the Website or any of the data presented on or by the Application or Website, or otherwise hosted or stored by AliveCor, for any commercial purpose other than as expressly permitted herein. You will notify AliveCor immediately upon any suspected unauthorized use of the Application or Website, whether by You or a third party, or any suspected loss of or suspected unauthorized access to passwords or other log-in information used by You to access the Application or Website ("Login Credentials"). You shall be solely responsible for maintaining the confidence and security of any Login Credentials, and AliveCor shall bear no liability or other responsibility associated therewith.

7. PRIVACY NOTICE

7.1. Privacy Notice

You explicitly consent to the collection, hosting, use, disclosure and other processing or handling of information as described in the AliveCor Privacy Notice located at http://alivecor.com/privacy, as such notice may be amended by AliveCor in its sole discretion from time to time. Upon modification of the Privacy Notice, AliveCor will notify You for Your consent.

8. SUSPENSION AND TERMINATION

8.1. Suspension

AliveCor may suspend or limit Your access to the Application or the Website if AliveCor, in its sole discretion, suspects that You are in violation of, or reasonably likely to be in violation of, these Terms and Conditions or any additional instructions, guidelines or policies issued by AliveCor, including those posted in the Application or on the Website.

8.2. Non-Renewal

You may cancel Your registration at any time in accordance with the procedures described on the Website.

8.3. Termination by AliveCor

AliveCor may terminate Your registration, including Your right to access and use the Website or the Application, in whole or in part, as follows:

For Cause. Immediately (1) in order to comply with applicable Law or instructions from any governmental agency or authority; (2) if AliveCor, in its sole discretion, suspects that You are using the Application or Website in a manner not permitted by these Terms and Conditions; or (3) upon any breach of these Terms and Conditions by You.

For Convenience. For convenience, upon notice by AliveCor to You.

8.4. Effect of Termination

Upon the expiration or earlier termination of Your registration or rights to use the Application or Website, for any reason (i) You will no longer be authorized to access or use the Application or Website or otherwise use any of the features or services offered by or through the Application or Website; (ii) AliveCor may delete any data associated with You or Your account; and (iii) all rights and obligations of the Parties under these Terms and Conditions shall expire, except those rights and obligations under those sections specifically designated in Section 8.5.

8.5. Survival of Selected Provisions

Notwithstanding the expiration or earlier termination of Your registration or rights to use the Application or Website, the following sections of the Terms and Conditions shall survive any such expiration or termination: Sections 3.2, 6.1, 8.4, 9, 10, 11 and 12.

9. LIABILITY

9.1. Limitation of Liability

TO THE MAXIMUM EXTENT PERMITTED BY APPLICABLE LAW, THE ALIVECOR GROUP (AS DEFINED IN SECTION 10.1) SHALL NOT BE LIABLE TO YOU FOR ANY DIRECT, INDIRECT, SPECIAL, INCIDENTAL, PUNITIVE OR CONSEQUENTIAL DAMAGES ARISING OUT OF OR RESULTING FROM YOUR USE OF THE APPLICATION OR WEBSITE, OR BASED UPON ANY BREACH OF ANY EXPRESS OR IMPLIED WARRANTY, BREACH OF CONTRACT (INCLUDING THESE TERMS AND CONDITIONS AND ANY ADDITIONAL INSTRUCTIONS, GUIDELINES OR POLICIES ISSUED BY ALIVECOR, INCLUDING THOSE POSTED IN THE APPLICATION OR ON THE WEBSITE), NEGLIGENCE, TORT OR ANY OTHER LEGAL THEORY (COLLECTIVELY, THE "EXCLUDED DAMAGES"). FOR THE AVOIDANCE OF DOUBT, THE EXCLUDED DAMAGES ALSO INCLUDE WITHOUT LIMITATION, LOSS OF SAVINGS OR REVENUE; LOSS OF PROFIT; LOSS OF USE; LOSS OF LIFE OR HEALTH, THE CLAIMS OF THIRD PARTIES; AND ANY COST OF ANY SUBSTITUTE EQUIPMENT OR SERVICES. IF ALIVECOR CANNOT LAWFULLY DISCLAIM LIABILITY FOR ANY OF THE FOREGOING DAMAGES, THEN ALIVECOR GROUP'S MAXIMUM LIABILITY TO YOU IS LIMITED TO THE PURCHASE PRICE PAID BY YOU TO ALIVECOR FOR THE APPLICATION. THESE LIMITATIONS APPLY EVEN IF ANY OTHER REMEDIES AVAILABLE TO YOU FAIL OF THEIR ESSENTIAL PURPOSE. SOME JURISDICTIONS DO NOT ALLOW THE EXCLUSION OR LIMITATION OF INCIDENTAL OR CONSEQUENTIAL DAMAGES, SO THE ABOVE LIMITATION MAY NOT APPLY TO YOU.

9.2. Remedies

At its option, any member of the AliveCor Group may seek all remedies available to it under law and in equity, including injunctive relief in the form of specific performance to enforce

these Terms and Conditions, including any additional instructions, guidelines or policies issued by AliveCor, including those posted in the Application or on the Website, and/or actions for damages.

9.3. Claims

No action arising out of, in connection with, or relating to these Terms and Conditions or the subject of these Terms and Conditions shall be brought by You more than one (1) year after the accrual of the cause of action. This period shall not be extended for any reason, except by the written consent of both parties. All statutes or provisions of law which would toll or otherwise affect the running of the period of limitation are hereby waived, and no such statute or provision of law shall operate to extend the period limited in this paragraph.

10. INDEMNITIES

10.1. Indemnity by You

You will indemnify and hold harmless AliveCor, its affiliates and their respective current, future or former officers, directors, partners, equity holders, employees, agents, contractors, and their successor or assigns (collectively, the "AliveCor Group") on demand, from and against any and all fines, penalties, liabilities, losses and other damages of any kind whatsoever (including attorneys' and experts' fees), incurred by any of the members of the AliveCor Group, and, if directed by AliveCor, shall defend the AliveCor Group against (1) all claims that any information, data or other materials provided by You in connection with your use of the Website or the Application (including Your application for registration to access and use the Application or Website) or these Terms and Conditions, or use thereof by any member of the AliveCor Group, infringes, misappropriates or otherwise violates any patent, trademark, copyright, trade secret or other intellectual property or proprietary right of a third party; (2) all claims arising from fraud committed by, or the intentional misconduct, criminal acts or gross negligence of, You; and (3) all claims otherwise arising due to a failure by You to comply with any term or condition of these Terms and Conditions, including any additional instructions, guidelines or policies issued by AliveCor, including those posted in the Application or on the Website.

11. DISPUTE RESOLUTION

11.1. Disputes

If a dispute arises under these Terms and Conditions between You and any member of the AliveCor Group, such dispute shall be resolved, at the filing party's election, in either a small claims court or by final and binding arbitration administered by the National Arbitration Forum or the American Arbitration Association, under their rules for consumer arbitrations. All disputes in arbitration will be handled solely between the named parties, and not on any representative or class basis. ACCORDINGLY, YOU ACKNOWLEDGE THAT IT MAY NOT HAVE ACCESS TO A COURT (OTHER THAN A SMALL CLAIMS COURT) OR JURY. Notwithstanding any other provision of these Terms and Conditions, any member of the AliveCor Group may resort to court action for injunctive relief at any time if, in its good faith belief, the dispute resolution procedures described in this Section 11.1 would permit or cause irreparable injury to such member of the AliveCor Group or any third party claiming against a member of the AliveCor Group, due to delay arising out of such dispute resolution procedures.

11.2. Governing Law

All rights and obligations of You or the AliveCor Group relating to these Terms and Conditions, including any additional instructions, guidelines or policies issued by AliveCor, including those posted in the Application or on the Website, shall be governed by and construed in accordance with the Law of the United States of America, State of New York without giving effect to any choice-of-law provision or rule (whether of the State of New York or any other jurisdiction) that would cause the application of the Laws of any other jurisdiction.

12. GENERAL

12.1. Entire Agreement, Amendments and Modifications

These Terms and Conditions any additional instructions, guidelines or policies issued by AliveCor, including those posted in the Application or on the Website constitute the entire agreement of the Parties with regard to Your use of the Application and the Website, and all matters addressed herein, and all prior agreements, letters, proposals, discussions and other documents regarding the Application or the Website and the matters herein are superseded and merged into these Terms and Conditions.

12.2. Force Majeure

AliveCor will be excused from performance under these Terms and Conditions for any period that it is prevented from or delayed in performing any obligations pursuant to these Terms and Conditions, in whole or in part, as a result of a Force Majeure Event. For purposes of this Section 12.2, "Force Majeure Event" means an event or series of events caused by or resulting from any of the following: (1) weather conditions or other elements of nature or acts of God; (2) acts of war, acts of terrorism, insurrection, riots, civil disorders or rebellion; (3) quarantines or embargoes, (4) labor strikes; (4) telecommunications, network, computer, server or Internet downtime; (5) unauthorized access to AliveCor's information technology systems by third parties; or (6) other causes beyond the reasonable control of AliveCor.

12.3. Severability

If any provision of these Terms and Conditions shall be held to be invalid, illegal or unenforceable, the validity, legality and enforceability of the remaining provisions shall not in any way be affected or impaired thereby, and such provision shall be deemed to be restated to reflect the Parties' original intentions as nearly as possible in accordance with applicable Law(s).

12.4. Assignment

You may not assign these Terms and Condition (or any rights, benefits or obligations hereunder) by operation of law or otherwise without the prior written consent of AliveCor, which may be withheld at AliveCor's sole discretion. Any attempted assignment by You that does not comply with the terms of this Section 12.4 shall be null and void. AliveCor may assign these Terms and Conditions, in whole or in part, to any third party in its sole discretion.

Appendix 12: iBG Star Owner's Guide

Patients will receive a hard copy and web link for the Owner's Guide.

[iBG Star Blood Glucose Monitoring System Owner's Guide]

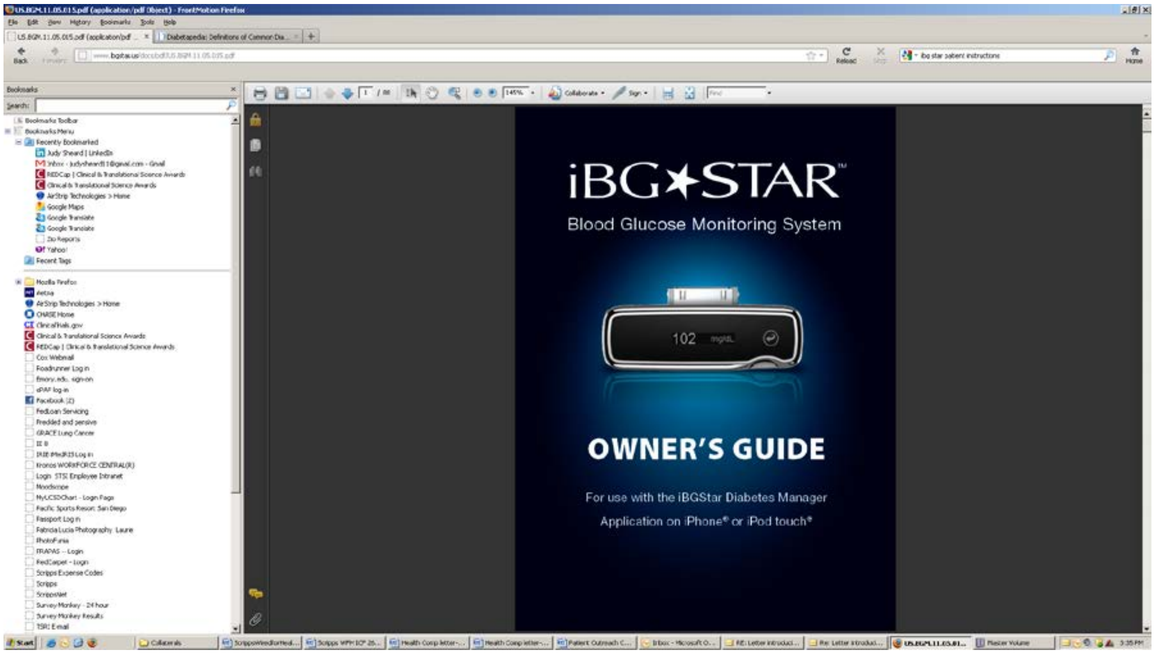

Appendix 13: Withings Blood Pressure Monitor User Manual

Patients will receive a hard copy and web link for the User Manual.

Withings Blood Pressure Monitor User Manual

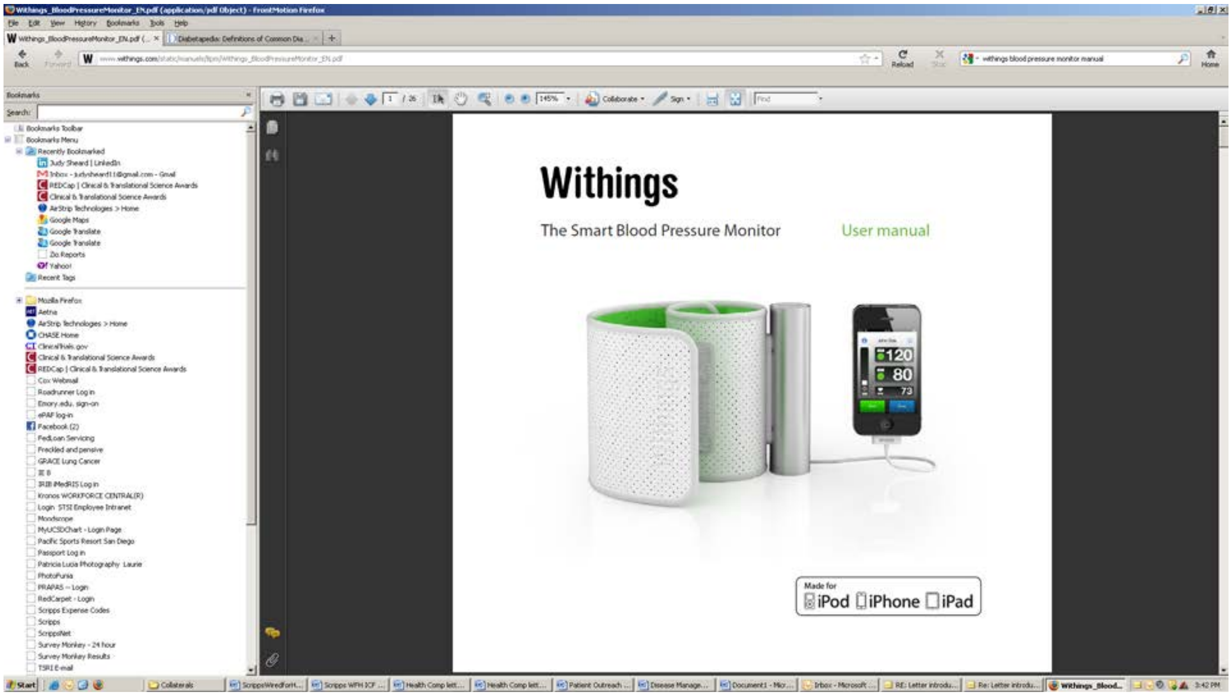

Appendix 14: Instructions for AliveCor ECG

Patients will receive a hard copy of the AliveCor Quick Start Guide.

AliveCor™

HEART MONITOR

Compatible with: iPhone® 4 / 4S

QUICK START GUIDE FOR DEMONSTRATION PURPOSES

PRODUCT DESCRIPTION

The AliveCor™ Heart Monitor is a mobile, clinical-quality electrocardiogram (ECG) recorder for use by both physicians and patients. The duration of the ECG recording is customizable from 30 seconds to continuous. The software application can store thousands of recordings on your iPhone 4 or 4S, and these recordings are also accessible to you on the AliveCor, Inc. server. The device consists of two components:

- The Heart Monitor, which snaps onto the iPhone and contains sensors and electronics used to collect ECG rhythms and transmit them to the iPhone.
- The AliveECG mobile application (AliveECG App) used to collect, view, save, and wirelessly transmit ECG recordings to the AliveCor server.

The Heart Monitor enables the physician or patient to:

- Collect and view single-channel ECG rhythms using the iPhone.
- Store ECG recordings on the iPhone.
- Wirelessly transmit ECG recordings to the AliveCor server.
- Access ECG recordings stored on the AliveCor server from anywhere in the world, and print the recording or save it to a hard drive.

DOWNLOAD THE ALIVEECG APP

Please contact Jessica Dinsdale (jessica@alivecor.com / 860-604-8754), Senior Clinical Engineer at AliveCor, to receive instructions on how to download the app to your iPhone 4 or 4S. As the device is not cleared by the FDA for sale in the United States, we are unable to provide an app in the App Store for download, therefore the app will need to be downloaded directly.

SET UP AN ALIVECOR ACCOUNT

You will use your AliveCor account to access, print, and save your ECG recordings stored on the AliveCor server. Follow the instructions presented when you open the AliveECG App for the first time. You can go back later and change your information if necessary.

RECORD ECG RHYTHMS USING THE HEART MONITOR

Before taking your first recording, read these instructions carefully and make sure you observe the following instructions each time you take a recording.

- Make sure the Heart Monitor is properly attached to your iPhone.
- Disconnect headphones, charger cables, or any other connected devices
- Clean the two electrodes with an alcohol-based sanitizer.
- Using your iPhone, launch the AliveECG App.
- Hold the system using two hands; the left hand should contact the electrode closer to the top of the iPhone, and the right hand should contact the electrode closer to the bottom of the iPhone. Recording will begin automatically when both electrodes make contact with skin. Alternatively, the Heart Monitor can be placed on the patient's chest to obtain a recording.

The ECG rhythms will appear best when the Heart Monitor is held steady. It may be helpful to rest your arms on a flat surface to increase stability.

If you remove contact before 10 seconds of recording has occurred, the ECG will not be saved, and you will not be able to review it.

ACCESS AND VIEW PREVIOUSLY RECORDED ECG RHYTHMS

You can view previously recorded ECGs within the AliveECG App or by logging into your account at www.alivecor.com.

Protected health information (ECGs) and personally identifiable information are encrypted in transit and at rest. ECGs are stored on fully secure, HIPAA-compliant servers and data is only accessible via your user name and password.

INDICATIONS FOR USE

The Heart Monitor is intended for use by licensed medical professionals or patients to record, display, store and transfer single-channel electrocardiogram (ECG) rhythms. There are no known contraindications for the Heart Monitor, although care should be taken when using the device on infants weighing less than 10kg. AliveCor does not recommend using on patients with a cardiac pacemaker or other implanted electronic devices.

CAUTION: Federal (USA) law restricts the sale of this device to or on the order of a physician.

INTENDED USER

The device is intended to be used by licensed medical professionals or by a patient under the care and supervision of a physician.

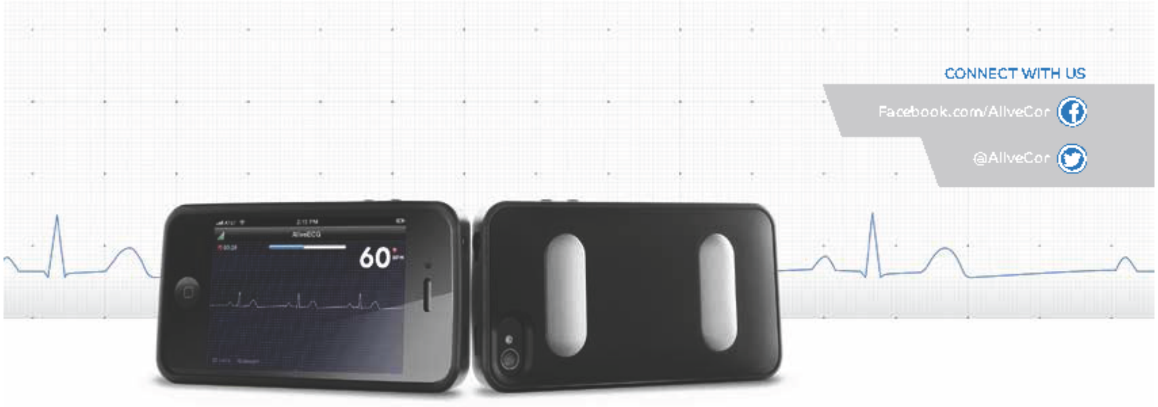

GENERAL SAFETY PRECAUTIONS

- The device should not be used in conjunction with water, or in a wet environment.
- Do not sterilize this unit with an autoclave or glass sterilizer.
- Audio and video products and similar equipment may cause interference. Please stay away from such equipment when you are recording.
- Disperse any static electricity from your body before using the unit.
- Do not take recordings in a moving vehicle.
- Do not expose the unit to strong shocks or vibrations.
- Do not disassemble, repair, or modify the unit.
- Do not insert battery with polarity reversed.
- Do not use batteries of a type other than that specified for use with the device.
- Do not take a recording if the electrodes are dirty. Clean them first.
- Do not use for any purpose other than obtaining an electrocardiogram.
- Do not take recordings in a location where the unit will be exposed to strong electromagnetic forces, such as near an arc welder, high-power radio transmitter, etc.
- Do not use this unit in locations subject to high or low temperatures or humidity. It should be used within the temperature and humidity range according to the product label.
- If the portion of the body where the electrode is applied has too much body fat, body hair or very dry skin, a successful recording may not be possible.
- Do not place this unit onto metal surfaces. This will deplete the battery.

STORAGE, HANDLING AND MAINTENANCE

Do not store the unit in:

- Locations exposed to direct sunlight
- Locations subject to high temperatures and high humidity
- Wet or damp locations where water may get on the unit
- Dusty locations
- Near fires or open flames
- Locations exposed to strong vibration
- Locations exposed to strong electromagnetic fields.

No maintenance of this system is required, except:

- The battery should be replaced when necessary.
- The electrodes should be cleaned using an alcohol-based sanitizer before each use.

WARNINGS

- This device is not designed or intended for complete diagnosis of cardiac conditions.
- This device measures heart rate and heart rhythm only.
- This device does not detect or measure all heart rate, heart rhythm and heart waveform changes, especially those related to ischemic heart conditions.
- Do not attempt self-diagnosis or self-treatment based on the recording results and analysis. Self-diagnosis or self-treatment may lead to deterioration of your health.
- Patients should always consult their physician if they notice changes in their health, regardless of recording results.
- Do not use this device with a defibrillator.
- Do not use in the presence of flammable anesthetics, drugs or pressurized oxygen (such as in a hyperbaric chamber, ultraviolet sterilizer or oxygen tent).
- Do not use this device during an MRI scan.
- Keep out of reach of infants, small children, or anyone incapable of using the device properly.
- ECG reports viewed or printed at any magnification other than 100% may appear distorted and could lead to misdiagnosis.

REMOVE THE HEART MONITOR FROM YOUR IPHONE

To remove the device from your iPhone, press gently on the iPhone through the camera hole while holding the device from the edge. The iPhone should partially pop out of the device and be easily removed.

© Copyright 2011-2012 AliveCor, Inc. All Rights Reserved. Patents Pending. AliveCor is a trademark and trade name of AliveCor, Inc. in the United States and other countries.

01LB02 Rev. A | October 2012

Appendix 15:

Healthy Circles Sample iPhone Display

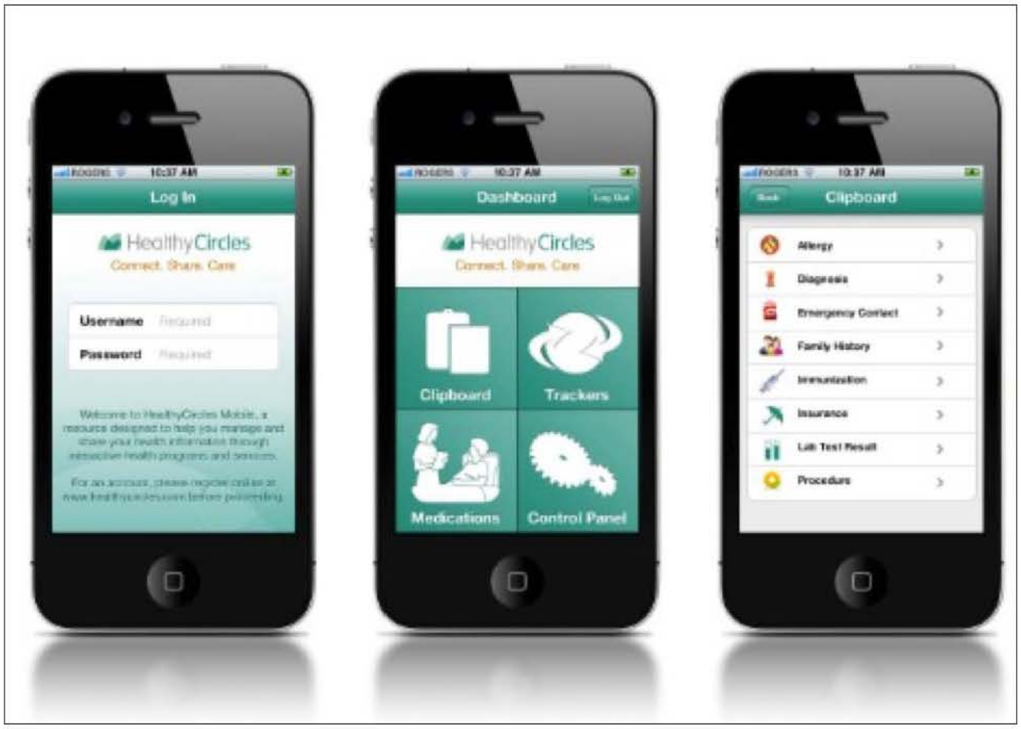

Appendix 16:

Text for Study Landing Page (http://wiredforhealth.healthcomp.com)

Wired for Health

The Wired for Health Study is a randomized clinical study that will evaluate the impact of remote wireless monitoring for patients with diabetes, hypertension, and arrhythmias.

This study will test whether wireless monitoring can reduce health care costs and increase health self-management for patients with chronic health conditions.

About the Study

A total of 100 individuals in the Monitoring group will be provided with i-Phones and the wireless device(s) most appropriate for monitoring their particular medical diagnosis. Patients will use the devices for a period of six months. The study will look at the patients' health care insurance claims and compare them to the insurance claims of the 100-person Control group.

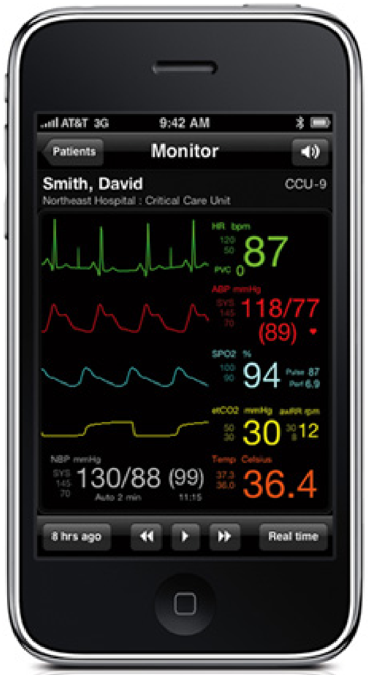

For the study, Health Comp is working with these companies:

Scripps Health [http://www.scripps.org/]

Scripps Translational Science Institute [http://www.stsiweb.org/]

Qualcomm Life [http://www.qualcommlife.com/]

HealthyCircles [http://www.healthycircles.com/]

Accenture Technology Labs [http://www.accenture.com]

Frequently Asked Questions

Who is selected to participate?
A person is potentially eligible to participate in this study if he or she has diabetes, hypertension, or a cardiac arrhythmia.

Who is Health Comp?
Health Comp is responsible for processing all health care insurance claims for employees and dependents of Scripps Health. Health Comp also administers health and wellness

programs to Scripps Employees and dependents and will the primary point of contact for study participants.

Why are so many different companies involved in this study?
This study has several different components, including i-Phones, three wireless health monitoring devices, iPhone apps, and a health tracking and education portal. Each company involved in the study is responsible for a different component.

Will participants interact with all of these companies?
No, only Health Comp clinical staff and Scripps Translational Science Institute research coordinators will work with the study participants.

Are patients' personal physicians involved in the study?
Participants' physicians are not part of this study and will not receive testing results unless a participant provides it to him or her. Health Comp clinical staff and researchers at the Scripps Translational Science Institute will receive and house participants' results, but only for research purposes.

Will participants benefit from participating in this research?
Upon completion of the study, participants will receive a $20 gift card, in addition to 10 Scripps Health Wellness points. If a Scripps employee earns 15 Wellness points in a calendar year (through participating in other Scripps Wellness activities), they are eligible for a reduction in health insurance costs.

What will happen to participants if they agree to be in the study?
If a participant agrees to be in the study, he or she will be assigned to one of two groups. Participants in the Monitoring Group will be given a wireless device, trained on how to use it, and will be asked to self-monitor vitals using the device(s) for 6 months. Participants in the Control Group will not receive a monitoring device or be asked to self-monitor vitals.

Will participants' employer or health insurance company be able to find out their results?
Participants' research results will be used for research purposes only. They will not become part of Scripps employment, medical, or insurance records.

IMPORTANT: BY USING YOUR iPHONE, YOU ARE AGREEING TO BE BOUND BY THE FOLLOWING APPLE AND THIRD PARTY TERMS:

A. APPLE iPHONE SOFTWARE LICENSE AGREEMENT
B. NOTICES FROM APPLE
C. GOOGLE MAPS TERMS AND CONDITIONS
D. YOUTUBE TERMS AND CONDITIONS

APPLE INC.
iPHONE SOFTWARE LICENSE AGREEMENT
Single Use License
PLEASE READ THIS SOFTWARE LICENSE AGREEMENT ("LICENSE") CAREFULLY BEFORE USING YOUR iPHONE OR DOWNLOADING THE SOFTWARE UPDATE ACCOMPANYING THIS LICENSE. BY USING YOUR iPHONE OR DOWNLOADING THIS SOFTWARE UPDATE, AS APPLICABLE, YOU ARE AGREEING TO BE BOUND BY THE TERMS OF THIS LICENSE, UNLESS YOU RETURN THE iPHONE IN ACCORDANCE WITH APPLE'S RETURN POLICY. IF YOU DO NOT AGREE TO THE TERMS OF THIS LICENSE, DO NOT USE THE iPHONE OR DOWNLOAD THIS SOFTWARE UPDATE. IF YOU DO NOT AGREE TO THE TERMS OF THE LICENSE, YOU MAY RETURN THE iPHONE WITHIN THE RETURN PERIOD TO THE APPLE STORE OR AUTHORIZED DISTRIBUTOR WHERE YOU OBTAINED IT FOR A REFUND, SUBJECT TO APPLE'S RETURN POLICY FOUND AT http://www.apple.com/legal/sales_policies/.

1. General. The software (including Boot ROM code and other embedded software), documentation, interfaces, content, fonts and any data that came with your iPhone ("Original iPhone Software"), as may be updated or replaced by feature enhancements, software updates or system restore software provided by Apple ("iPhone Software Updates"), whether in read only memory, on any other media or in any other form (the Original iPhone Software and iPhone Software Updates are collectively referred to as the "iPhone Software") are licensed, not sold, to you by Apple Inc. ("Apple") for use only under the terms of this License. Apple and its licensors retain ownership of the iPhone Software itself and reserve all rights not expressly granted to you.

Apple, at its discretion, may make available future updates to the operating system software for your iPhone. Updates, if any, may not necessarily include all existing software features, or new features that Apple releases for newer iPhone models.

2. Permitted License Uses and Restrictions.

(a) Subject to the terms and conditions of this License, you are granted a limited non-exclusive license to use the iPhone Software on a single Apple-branded iPhone. Except as permitted in Section 2(b) below, this License does not allow the iPhone Software to exist on more than one Apple-branded iPhone at a time or on any other phone, and you may not distribute or make the iPhone Software available over a network where it could be used by multiple devices at the same time. This License does not grant you any rights to use Apple proprietary interfaces and other intellectual property in the design, development, manufacture, licensing or distribution of third party devices and accessories, or third party software applications, for use with the iPhone. Some of those rights are available under separate licenses from Apple. For more information on developing third party devices and accessories for the iPhone, please email madeforipod@apple.com. For more information on developing software applications for the iPhone, please email devprograms@apple.com.

(b) Subject to the terms and conditions of this License, you are granted a limited non-exclusive license to download iPhone Software Updates that may be made available by Apple for your model of the iPhone to update or restore the software on any such iPhone that you own or control. This License does not allow you to update or restore iPhones that you do not control or own, and you may not distribute or make the iPhone Software Updates available over a network where they could be used by multiple devices or multiple computers at the same time. You may make one copy of the iPhone Software Updates stored on your computer in machine-readable form for backup purposes only, provided that the backup copy must include all copyright or other proprietary notices contained on the original.

(c) You may not and you agree not to, or to enable others to, copy (except as expressly permitted by this License), decompile, reverse engineer, disassemble, attempt to derive the source code of, decrypt, modify, or create derivative works of the iPhone Software or any services provided by the iPhone Software, or any part thereof (except as and only to the extent any foregoing restriction is prohibited by applicable law or to the extent as may be permitted by licensing terms governing use of open-sourced components included with the iPhone Software). Any attempt to do so is a violation of the rights of Apple and its licensors of the iPhone Software.
(d) By storing content on your iPhone you are making a digital copy. In some jurisdictions, it is unlawful to make digital copies without prior permission from the rightsholder. The iPhone Software may be used to reproduce materials so long as such use is limited to reproduction of non-copyrighted materials, materials in which you own the copyright, or materials you are authorized or legally permitted to reproduce.
(e) You agree to use the iPhone Software and the Services (as defined in Section 5 below) in compliance with all applicable laws, including local laws of the country or region in which you reside or in which you download or use the iPhone Software and Services.
3. Transfer. You may not rent, lease, lend, sell, redistribute, or sublicense the iPhone Software. You may, however, make a one-time permanent transfer of all of your license rights to the iPhone Software to another party in connection with the transfer of ownership of your iPhone, provided that: (a) the transfer must include your iPhone and all of the iPhone Software, including all its component parts, original media, printed materials and this License; (b) you do not retain any copies of the iPhone Software, full or partial, including copies stored on a computer or other storage device; and (c) the party receiving the iPhone Software reads and agrees to accept the terms and conditions of this License.
4. Consent to Use of Data.
(a) Anonymous Diagnostic and Usage Data. You agree that Apple and its subsidiaries and agents may collect, maintain, process and use diagnostic, technical, usage and related information, including but not limited to information about your iPhone, computer, system and application software, and peripherals, that is gathered periodically to facilitate the provision of software updates, product support and other services to you (if any) related to the iPhone Software, and to verify compliance with the terms of this License. Apple may use this information, as long as it is collected anonymously in a form that does not personally identify you, to improve our products or to provide services or technologies to you.
(b) Location Data. Apple and its partners and licensees may provide certain services through your iPhone that rely upon location information. To provide and improve these services, where available, Apple and its partners and licensees may transmit, collect, maintain, process and use your location data, including the real-time geographic location of your iPhone, and location search queries. The location data and queries collected by Apple are collected in a form that does not personally identify you and may be used by Apple and its partners and licensees to provide and improve location-based products and services. **By using any location-based services on your iPhone, you agree and consent to Apple's and its partners' and licensees' transmission, collection, maintenance, processing and use of your location data and queries to provide and improve such products and services.** You may withdraw this consent at any time by going to the Location Services setting on your iPhone and either turning off the global Location Services setting or turning off the individual location settings of each location-aware application on your iPhone. Not using these location features will not impact the non location-based functionality of your iPhone. When using third party applications or services on the iPhone that use or provide location data, you are subject to and should review such third party's terms and privacy policy on use of location data by such third party applications or services.
(c) Video Calls. The FaceTime video call feature of the iPhone Software ("FaceTime") requires Internet access and may not be available in all countries or regions. Your use of FaceTime is subject to your compliance with Section 2(e) above and you understand that your iPhone's telephone number will be displayed to the other party on the FaceTime video call, even if you have a blocked number. **By using the iPhone Software, you agree that Apple may use and maintain your iPhone's telephone number as a unique account identifier for the purpose of providing**

and improving the FaceTime feature. You may turn off the FaceTime feature by going to the FaceTime Setting on your iPhone and turning it off or by going to the Restrictions setting on your
iPhone and enabling the FaceTime restriction.
(d) Interest-Based Advertising. Apple may provide mobile, interest-based advertising to you. If you do not want to receive relevant ads on your iPhone, you can opt out by going to this link on
your iPhone: http://oo.apple.com. If you opt out, you will continue to receive the same number of mobile ads, but they may be less relevant because they will not be based on your interests. You
may still see ads related to the content on a web page or in an application or based on other non-personal information. This opt-out applies only to Apple advertising services and does not affect
interest-based advertising from other advertising networks.
At all times your information will be treated in accordance with Apple's Privacy Policy, which is incorporated by reference into this License and can be viewed at: www.apple.com/legal/privacy/.
5. Services and Third Party Materials.
(a) The iPhone Software enables access to Apple's iTunes Store, App Store, Game Center, and other Apple and third party services and web sites (collectively and individually, "Services"). Use
of the Services requires Internet access and use of certain Services requires you to accept additional terms. By using this software in connection with an iTunes Store account or a Game Center
account, you agree to the latest iTunes Store Terms and Conditions and/or Game Center Terms and Conditions, which you may access and review at http://www.apple.com/legal/itunes/ww/.
(b) You understand that by using any of the Services, you may encounter content that may be deemed offensive, indecent, or objectionable, which content may or may not be identified as
having explicit language, and that the results of any search or entering of a particular URL may automatically and unintentionally generate links or references to objectionable material.
Nevertheless, you agree to use the Services at your sole risk and that Apple shall have no liability to you for content that may be found to be offensive, indecent, or objectionable.
(c) Certain Services may display, include or make available content, data, information, applications or materials from third parties ("Third Party Materials") or provide links to certain third party web
sites. By using the Services, you acknowledge and agree that Apple is not responsible for examining or evaluating the content, accuracy, completeness, timeliness, validity, copyright compliance,
legality, decency, quality or any other aspect of such Third Party Materials or web sites. Apple, its officers, affiliates and subsidiaries do not warrant or endorse and do not assume and will not
have any liability or responsibility to you or any other person for any third-party Services, Third Party Materials or web sites, or for any other materials, products, or services of third parties. Third
Party Materials and links to other web sites are provided solely as a convenience to you.
(d) Financial information displayed by any Services is for general informational purposes only and should not be relied upon as investment advice. Before executing any securities transaction
based upon information obtained through the Services, you should consult with a financial or securities professional who is legally qualified to give financial or securities advice in your country or
region. Location data provided by any Services is for basic navigational purposes only and is not intended to be relied upon in situations where precise location information is needed or where
erroneous, inaccurate, time-delayed or incomplete location data may lead to death, personal injury, property or environmental damage. Neither Apple nor any of its content providers guarantees
the availability, accuracy, completeness, reliability, or timeliness of stock information, location data or any other data displayed by any Services.
(e) You agree that the Services contain proprietary content, information and material that is owned by Apple and/or its licensors, and is protected by applicable intellectual property and other
laws, including but not limited to copyright, and that you will not use such proprietary content, information or materials in any way whatsoever except for permitted use of the Services or in any
manner that is inconsistent with the terms of this License or that infringes any intellectual property rights of a third party or Apple. No portion of the Services may be reproduced in any form or by
any means. You agree not to modify, rent, lease, loan, sell, distribute, or create derivative works based on the Services, in any manner, and you shall not exploit the Services in any unauthorized
way whatsoever, including but not limited to, using the Services to transmit any computer viruses, worms, trojan horses or other malware, or by trespass or burdening network capacity. You

further agree not to use the Services in any manner to harass, abuse, stalk, threaten, defame or otherwise infringe or violate the rights of any other party, and that Apple is not in any way
responsible for any such use by you, nor for any harassing, threatening, defamatory, offensive, infringing or illegal messages or transmissions that you may receive as a result of using any of the
Services.
(f) In addition, Services and Third Party Materials that may be accessed from, displayed on or linked to from the iPhone are not available in all languages or in all countries or regions. Apple
makes no representation that such Services and Materials are appropriate or available for use in any particular location. To the extent you choose to use or access such Services and Materials,
you do so at your own initiative and are responsible for compliance with any applicable laws, including but not limited to applicable local laws. Apple and its licensors reserve the right to change,
suspend, remove, or disable access to any Services at any time without notice. In no event will Apple be liable for the removal of or disabling of access to any such Services. Apple may also
impose limits on the use of or access to certain Services, in any case and without notice or liability.
6. Termination. This License is effective until terminated. Your rights under this License will terminate automatically or otherwise cease to be effective without notice from Apple if you fail to
comply with any term(s) of this License. Upon the termination of this License, you shall cease all use of the iPhone Software. Sections 5, 6, 7, 8, 9, 12 and 13 of this License shall survive any
such termination.
7. Disclaimer of Warranties.
7.1 If you are a customer who is a consumer (someone who uses the iPhone Software outside of your trade, business or profession), you may have legal rights in your country of residence
which would prohibit the following limitations from applying to you, and where prohibited they will not apply to you. To find out more about rights, you should contact a local consumer advice
organization.
7.2 YOU EXPRESSLY ACKNOWLEDGE AND AGREE THAT, TO THE EXTENT PERMITTED BY APPLICABLE LAW, USE OF THE iPHONE SOFTWARE AND SERVICES IS AT YOUR SOLE
RISK AND THAT THE ENTIRE RISK AS TO SATISFACTORY QUALITY, PERFORMANCE, ACCURACY AND EFFORT IS WITH YOU.
7.3 TO THE MAXIMUM EXTENT PERMITTED BY APPLICABLE LAW, THE iPHONE SOFTWARE AND SERVICES PERFORMED BY THE iPHONE SOFTWARE ARE PROVIDED "AS IS" AND
"AS AVAILABLE", WITH ALL FAULTS AND WITHOUT WARRANTY OF ANY KIND, AND APPLE AND APPLE'S LICENSORS (COLLECTIVELY REFERRED TO AS "APPLE" FOR THE
PURPOSES OF SECTIONS 7 AND 8) HEREBY DISCLAIM ALL WARRANTIES AND CONDITIONS WITH RESPECT TO THE iPHONE SOFTWARE AND SERVICES, EITHER EXPRESS, IMPLIED
OR STATUTORY, INCLUDING, BUT NOT LIMITED TO, THE IMPLIED WARRANTIES AND/OR CONDITIONS OF MERCHANTABILITY, SATISFACTORY QUALITY, FITNESS FOR A PARTICULAR
PURPOSE, ACCURACY, QUIET ENJOYMENT, AND NON-INFRINGEMENT OF THIRD PARTY RIGHTS.
7.4 APPLE DOES NOT WARRANT AGAINST INTERFERENCE WITH YOUR ENJOYMENT OF THE iPHONE SOFTWARE AND SERVICES, THAT THE FUNCTIONS CONTAINED IN OR
SERVICES PERFORMED BY THE iPHONE SOFTWARE WILL MEET YOUR REQUIREMENTS, THAT THE OPERATION OF THE iPHONE SOFTWARE AND SERVICES WILL BE
UNINTERRUPTED OR ERROR-FREE, THAT ANY SERVICE WILL CONTINUE TO BE MADE AVAILABLE, THAT DEFECTS IN THE iPHONE SOFTWARE OR SERVICES WILL BE CORRECTED,
OR THAT THE iPHONE SOFTWARE WILL BE COMPATIBLE OR WORK WITH ANY THIRD PARTY SOFTWARE, APPLICATIONS OR THIRD PARTY SERVICES. INSTALLATION OF THIS
SOFTWARE MAY AFFECT THE USABILITY OF THIRD PARTY SOFTWARE, APPLICATIONS OR THIRD PARTY SERVICES.
7.5 YOU FURTHER ACKNOWLEDGE THAT THE iPHONE SOFTWARE AND SERVICES ARE NOT INTENDED OR SUITABLE FOR USE IN SITUATIONS OR ENVIRONMENTS WHERE THE
FAILURE OR TIME DELAYS OF, OR ERRORS OR INACCURACIES IN, THE CONTENT, DATA OR INFORMATION PROVIDED BY THE IPHONE SOFTWARE OR SERVICES COULD LEAD TO
DEATH, PERSONAL INJURY, OR SEVERE PHYSICAL OR ENVIRONMENTAL DAMAGE, INCLUDING WITHOUT LIMITATION THE OPERATION OF NUCLEAR FACILITIES, AIRCRAFT

NAVIGATION OR COMMUNICATION SYSTEMS, AIR TRAFFIC CONTROL, LIFE SUPPORT OR WEAPONS SYSTEMS.
7.6 NO ORAL OR WRITTEN INFORMATION OR ADVICE GIVEN BY APPLE OR AN APPLE AUTHORIZED REPRESENTATIVE SHALL CREATE A WARRANTY. SHOULD THE iPHONE
SOFTWARE OR SERVICES PROVE DEFECTIVE, YOU ASSUME THE ENTIRE COST OF ALL NECESSARY SERVICING, REPAIR OR CORRECTION. SOME JURISDICTIONS DO NOT ALLOW
THE EXCLUSION OF IMPLIED WARRANTIES OR LIMITATIONS ON APPLICABLE STATUTORY RIGHTS OF A CONSUMER, SO THE ABOVE EXCLUSION AND LIMITATIONS MAY NOT
APPLY TO YOU.
8. Limitation of Liability. TO THE EXTENT NOT PROHIBITED BY APPLICABLE LAW, IN NO EVENT SHALL APPLE BE LIABLE FOR PERSONAL INJURY, OR ANY INCIDENTAL,
SPECIAL, INDIRECT OR CONSEQUENTIAL DAMAGES WHATSOEVER, INCLUDING, WITHOUT LIMITATION, DAMAGES FOR LOSS OF PROFITS, CORRUPTION OR LOSS OF DATA,
FAILURE TO TRANSMIT OR RECEIVE ANY DATA, BUSINESS INTERRUPTION OR ANY OTHER COMMERCIAL DAMAGES OR LOSSES, ARISING OUT OF OR RELATED TO YOUR USE OF
OR INABILITY TO USE THE iPHONE SOFTWARE AND SERVICES OR ANY THIRD PARTY SOFTWARE OR APPLICATIONS IN CONJUNCTION WITH THE iPHONE SOFTWARE, HOWEVER
CAUSED, REGARDLESS OF THE THEORY OF LIABILITY (CONTRACT, TORT OR OTHERWISE) AND EVEN IF APPLE HAS BEEN ADVISED OF THE POSSIBILITY OF SUCH DAMAGES.
SOME JURISDICTIONS DO NOT ALLOW THE LIMITATION OF LIABILITY FOR PERSONAL INJURY, OR OF INCIDENTAL OR CONSEQUENTIAL DAMAGES, SO THIS LIMITATION MAY NOT
APPLY TO YOU. In no event shall Apple's total liability to you for all damages (other than as may be required by applicable law in cases involving personal injury) exceed the amount of two
hundred and fifty dollars (U.S.$250.00). The foregoing limitations will apply even if the above stated remedy fails of its essential purpose.
9. Digital Certificates. The iPhone Software contains functionality that allows it to accept digital certificates either issued from Apple or from third parties. YOU ARE SOLELY RESPONSIBLE
FOR DECIDING WHETHER OR NOT TO RELY ON A CERTIFICATE WHETHER ISSUED BY APPLE OR A THIRD PARTY. YOUR USE OF DIGITAL CERTIFICATES IS AT YOUR SOLE RISK. TO
THE MAXIMUM EXTENT PERMITTED BY APPLICABLE LAW, APPLE MAKES NO WARRANTIES OR REPRESENTATIONS, EXPRESS OR IMPLIED, AS TO MERCHANTABILITY OR FITNESS
FOR ANY PARTICULAR PURPOSE, ACCURACY, SECURITY, OR NON-INFRINGEMENT OF THIRD PARTY RIGHTS WITH RESPECT TO DIGITAL CERTIFICATES.
10. Export Control. You may not use or otherwise export or re-export the iPhone Software except as authorized by United States law and the laws of the jurisdiction(s) in which the iPhone
Software was obtained. In particular, but without limitation, the iPhone Software may not be exported or re-exported (a) into any U.S. embargoed countries or (b) to anyone on the U.S. Treasury
Department's list of Specially Designated Nationals or the U.S. Department of Commerce Denied Person's List or Entity List. By using the iPhone Software, you represent and warrant that you
are not located in any such country or on any such list. You also agree that you will not use the iPhone Software for any purposes prohibited by United States law, including, without limitation,
the development, design, manufacture or production of missiles, nuclear, chemical or biological weapons.
11. Government End Users. The iPhone Software and related documentation are "Commercial Items", as that term is defined at 48 C.F.R. §2.101, consisting of "Commercial Computer
Software" and "Commercial Computer Software Documentation", as such terms are used in 48 C.F.R. §12.212 or 48 C.F.R. §227.7202, as applicable. Consistent with 48 C.F.R. §12.212 or 48
C.F.R. §227.7202-1 through 227.7202-4, as applicable, the Commercial Computer Software and Commercial Computer Software Documentation are being licensed to U.S. Government end
users (a) only as Commercial Items and (b) with only those rights as are granted to all other end users pursuant to the terms and conditions herein. Unpublished-rights reserved under the
copyright laws of the United States.
12. Controlling Law and Severability. This License will be governed by and construed in accordance with the laws of the State of California, excluding its conflict of law principles. This
License shall not be governed by the United Nations Convention on Contracts for the International Sale of Goods, the application of which is expressly excluded. If you are a consumer based in

the United Kingdom, this License will be governed by the laws of the jurisdiction of your residence. If for any reason a court of competent jurisdiction finds any provision, or portion thereof, to be unenforceable, the remainder of this License shall continue in full force and effect.

13. Complete Agreement; Governing Language. This License constitutes the entire agreement between you and Apple relating to the iPhone Software and supersedes all prior or contemporaneous understandings regarding such subject matter. No amendment to or modification of this License will be binding unless in writing and signed by Apple. Any translation of this License is done for local requirements and in the event of a dispute between the English and any non-English versions, the English version of this License shall govern, to the extent not prohibited by local law in your jurisdiction.

14. Third Party Acknowledgements. Portions of the iPhone Software may utilize or include third party software and other copyrighted material. Acknowledgements, licensing terms and disclaimers for such material are contained in the electronic documentation for the iPhone Software, and your use of such material is governed by their respective terms. Use of the Google Safe Browsing Service is subject to the Google Terms of Service (http://www.google.com/terms_of_service.html) and to Google's Privacy Policy (http://www.google.com/privacypolicy.html).

15. Use of MPEG-4; H.264/AVC Notice.

(a) The iPhone Software contains MPEG-4 video encoding and/or decoding functionality. The iPhone Software is licensed under the MPEG-4 Visual Patent Portfolio License for the personal and non-commercial use of a consumer for (i) encoding video in compliance with the MPEG-4 Visual Standard ("MPEG-4 Video") and/or (ii) decoding MPEG-4 video that was encoded by a consumer engaged in a personal and non-commercial activity and/or was obtained from a video provider licensed by MPEG LA to provide MPEG-4 video. No license is granted or shall be implied for any other use. Additional information including that relating to promotional, internal and commercial uses and licensing may be obtained from MPEG LA, LLC. See http://www.mpegla.com.

(b) The iPhone Software contains AVC encoding and/or decoding functionality, commercial use of H.264/AVC requires additional licensing and the following provision applies: THE AVC FUNCTIONALITY IN THE iPHONE SOFTWARE IS LICENSED HEREIN ONLY FOR THE PERSONAL AND NON-COMMERCIAL USE OF A CONSUMER TO (i) ENCODE VIDEO IN COMPLIANCE WITH THE AVC STANDARD ("AVC VIDEO") AND/OR (ii) DECODE AVC VIDEO THAT WAS ENCODED BY A CONSUMER ENGAGED IN A PERSONAL AND NON-COMMERCIAL ACTIVITY AND/OR AVC VIDEO THAT WAS OBTAINED FROM A VIDEO PROVIDER LICENSED TO PROVIDE AVC VIDEO. INFORMATION REGARDING OTHER USES AND LICENSES MAY BE OBTAINED FROM MPEG LA L.L.C. SEE HTTP://WWW.MPEGLA.COM.

16. Yahoo Search Service Restrictions. The Yahoo Search Service available through Safari is licensed for use only in the following countries and regions: Argentina, Aruba, Australia, Austria, Barbados, Belgium, Bermuda, Brazil, Bulgaria, Canada, Cayman Islands, Chile, Colombia, Cyprus, Czech Republic, Denmark, Dominican Republic, Ecuador, El Salvador, Finland, France, Germany, Greece, Grenada, Guatemala, Hong Kong, Hungary, Iceland, India, Indonesia, Ireland, Italy, Jamaica, Latvia, Lithuania, Luxembourg, Malaysia, Malta, Mexico, Netherlands, New Zealand, Nicaragua, Norway, Panama, Peru, Philippines, Poland, Portugal, Puerto Rico, Romania, Singapore, Slovakia, Slovenia, South Korea, Spain, St. Lucia, St. Vincent, Sweden, Switzerland, Taiwan, Thailand, The Bahamas, Trinidad and Tobago, Turkey, UK, Uruguay, US and Venezuela.

17. Microsoft Exchange Notice. The Microsoft Exchange mail setting in the iPhone Software is licensed only for over-the-air synchronization of information, such as email, contacts, calendar and tasks, between your iPhone and Microsoft Exchange Server or other server software licensed by Microsoft to implement the Microsoft Exchange ActiveSync protocol.

EA0654
Rev. 7/19/10

NOTICES FROM APPLE

If Apple needs to contact you about your product or account, you consent to receive the notices by email. You agree that any such notices that we send you electronically will satisfy any legal communication requirements.

GOOGLE MAPS TERMS AND CONDITIONS

Thank you for trying out the Google Maps for mobile software application! This page contains the terms and conditions (the "Terms and Conditions") for Google Maps for mobile and the enterprise version of Google Maps for mobile. In order to use this software, including any third party software made available to you in conjunction with this software and/or the related service, (collectively referred to below as "Google Maps for mobile") you agree to be bound by these Terms and Conditions, either on behalf of yourself or on behalf of your employer or other entity. If you are agreeing to be bound by these Terms and Conditions on behalf of your employer or other entity, you represent and warrant that you have full legal authority to bind your employer or such entity to these Terms and Conditions. If you don't have the legal authority to bind, please press "No" when asked whether you agree to these Terms and Conditions, and do not proceed with use of this product.

Additional Terms

Google Maps for mobile is designed to be used in conjunction with Google's Maps services and other Google services. Accordingly, you agree and acknowledge that your use of Google Maps for mobile is also subject to (a) the specific terms of service for Google Maps (which can be viewed at http://local.google.com/help/terms_local.html) including the content notices applicable thereto (which can be viewed at http://local.google.com/help/legalnotices_local.html), (b) the general Google terms of service (which can be viewed at http://www.google.com/terms_of_service.html) and (c) Google's overall privacy policy (which can be viewed at http://www.google.com/privacypolicy.html), as well as specific privacy policies, such as the Google Maps for mobile privacy policy included with this application, such provisions being hereby incorporated into these Terms and Conditions by reference. To the extent that there is any inconsistency or conflict between such additional terms and these Terms and Conditions, the provisions of these Terms and Conditions take precedence.

Network Charges

Google does not charge for downloading or using Google Maps for mobile, but depending on your plan and your carrier or provider, your carrier or other provider may charge you for downloading Google Maps for mobile or for use of your mobile phone when you access information or other Google services through Google Maps for mobile.

Non-Commercial Use Only

Google Maps for mobile is made available to you for your non-commercial use only. This means that you may use it for your personal use only: you may use it at work or at home, to search for anything you want, subject to the terms set out in these Terms and Conditions. You need to obtain Google's permission first, which you can do by contacting mobile-support@google.com, if you want to sell Google Maps for mobile or any information, services, or software associated with or derived from it, or if you want to modify, copy, license, or create derivative works from Google Maps for mobile.

Unless you have our prior written consent, you agree not to modify, adapt, translate, prepare derivative works from, decompile, reverse engineer, disassemble or otherwise attempt to derive source code from Google Maps for mobile.

Furthermore, you may not use Google Maps for mobile in any manner that could damage, disable, overburden, or impair Google's services (e.g., you may not use the Google Maps for mobile in an automated manner), nor may you use Google Maps for mobile in any manner that could interfere with any other party's use and enjoyment of Google's services.

If you have comments on Google Maps for mobile or ideas on how to improve it, please email mobile-support@google.com. Please note that by doing so, you also grant Google and third parties permission to use and incorporate your ideas or comments into Google Maps for mobile (or third party software) without further notice or compensation.

Intellectual Property

As between you and Google, you agree and acknowledge that Google owns all rights, title and interest in and to Google Maps for mobile, including without limitation all associated Intellectual

Property Rights. "Intellectual Property Rights" means any and all rights existing from time to time under patent law, copyright law, trade secret law, trademark law, unfair competition law, and any and all other proprietary rights, and any and all applications, renewals, extensions and restorations thereof, now or hereafter in force and effect worldwide. You agree to not remove, obscure, or alter Google's or any third party's copyright notice, trademarks, or other proprietary rights notices affixed to or contained within or accessed in conjunction with or through the Google Maps for mobile.

Disclaimer of Warranties

Google and any third party who makes its software available in conjunction with or through Google Maps for mobile disclaim any responsibility for any harm resulting from your use of Google Maps for mobile and/or any third party software accessed in conjunction with or through Google Maps for mobile. GOOGLE MAPS FOR MOBILE IS PROVIDED "AS IS," WITH NO WARRANTIES WHATSOEVER. GOOGLE AND SUCH THIRD PARTIES EXPRESSLY DISCLAIM TO THE FULLEST EXTENT PERMITTED BY LAW ALL EXPRESS, IMPLIED, AND STATUTORY WARRANTIES, INCLUDING, WITHOUT LIMITATION, THE WARRANTIES OF MERCHANTABILITY, FITNESS FOR A PARTICULAR PURPOSE, AND NON-INFRINGEMENT OF PROPRIETARY RIGHTS. GOOGLE AND ANY SUCH THIRD PARTIES DISCLAIM ANY WARRANTIES REGARDING THE SECURITY, RELIABILITY, TIMELINESS, AND PERFORMANCE OF GOOGLE MAPS FOR MOBILE AND SUCH THIRD PARTY SOFTWARE.

YOU UNDERSTAND AND AGREE THAT YOU DOWNLOAD AND/OR USE GOOGLE MAPS FOR MOBILE AT YOUR OWN DISCRETION AND RISK AND THAT YOU WILL BE SOLELY RESPONSIBLE FOR ANY DAMAGES TO YOUR COMPUTER OR MOBILE DEVICE SYSTEM OR LOSS OF DATA THAT RESULTS FROM THE DOWNLOAD OR USE OF GOOGLE MAPS FOR MOBILE. SOME STATES OR OTHER JURISDICTIONS DO NOT ALLOW THE EXCLUSION OF IMPLIED WARRANTIES, SO THE ABOVE EXCLUSIONS MAY NOT APPLY TO YOU. YOU MAY ALSO HAVE OTHER RIGHTS THAT VARY FROM STATE TO STATE AND JURISDICTION TO JURISDICTION.

Limitation of Liability

UNDER NO CIRCUMSTANCES SHALL GOOGLE OR ANY THIRD PARTY WHO MAKE THEIR SOFTWARE AVAILABLE IN CONJUNCTION WITH OR THROUGH THE GOOGLE MAPS FOR MOBILE BE LIABLE TO ANY USER ON ACCOUNT OF THAT USER'S USE OR MISUSE OF GOOGLE MAPS FOR MOBILE. SUCH LIMITATION OF LIABILITY SHALL APPLY TO PREVENT RECOVERY OF DIRECT, INDIRECT, INCIDENTAL, CONSEQUENTIAL, SPECIAL, EXEMPLARY, AND PUNITIVE DAMAGES WHETHER SUCH CLAIM IS BASED ON WARRANTY, CONTRACT, TORT (INCLUDING NEGLIGENCE), OR OTHERWISE, (EVEN IF GOOGLE AND/OR A THIRD PARTY SOFTWARE PROVIDER HAVE BEEN ADVISED OF THE POSSIBILITY OF SUCH DAMAGES). SUCH LIMITATION OF LIABILITY SHALL APPLY WHETHER THE DAMAGES ARISE FROM USE OR MISUSE OF AND RELIANCE ON GOOGLE MAPS FOR MOBILE OR ON PRODUCTS OR SERVICES MADE AVAILABLE IN CONJUNCTION WITH OR THROUGH GOOGLE MAPS FOR MOBILE, FROM INABILITY TO USE GOOGLE MAPS FOR MOBILE OR PRODUCTS OR SERVICES MADE AVAILABLE IN CONJUNCTION WITH OR THROUGH THE GOOGLE MAPS FOR MOBILE, OR FROM THE INTERRUPTION, SUSPENSION, OR TERMINATION OF GOOGLE MAPS FOR MOBILE OR PRODUCTS OR SERVICES MADE AVAILABLE IN CONJUNCTION WITH OR THROUGH GOOGLE MAPS FOR MOBILE (INCLUDING SUCH DAMAGES INCURRED BY THIRD PARTIES). SUCH LIMITATION SHALL APPLY NOTWITHSTANDING A FAILURE OF ESSENTIAL PURPOSE OF ANY LIMITED REMEDY AND TO THE FULLEST EXTENT PERMITTED BY LAW. SOME STATES OR OTHER JURISDICTIONS DO NOT ALLOW THE EXCLUSION OR LIMITATION OF LIABILITY FOR INCIDENTAL OR CONSEQUENTIAL DAMAGES, SO THE ABOVE LIMITATIONS AND EXCLUSIONS MAY NOT APPLY TO YOU.

Miscellaneous Provisions

These Terms and Conditions will be governed by and construed in accordance with the laws of the State of California, without giving effect to the conflict of laws provisions of California or your actual state or country of residence. If for any reason a court of competent jurisdiction finds any provision or portion of these Terms and Conditions to be unenforceable, the remainder of these Terms and Conditions will continue in full force and effect.

These Terms and Conditions constitute the entire agreement between you and Google with respect to the subject matter hereof and supersede and replace all prior or contemporaneous understandings or agreements, written or oral, regarding such subject matter. Any waiver of any provision of these Terms and Conditions will be effective only if in writing and signed by Google.
September 2007

YOUTUBE TERMS OF SERVICE

1. Your Acceptance

A. By using and/or visiting this website (collectively, including all content and functionality available through the YouTube.com domain name, the "YouTube Website", or "Website"), you signify your agreement to (1) these terms and conditions (the "Terms of Service"), (2)YouTube's privacy notice, found at http://www.youtube.com/t/privacy and incorporated here by reference, and (3) YouTube's Community Guidelines, found at http://www.youtube.com/t/community_guidelines and also incorporated here by reference. If you do not agree to any of these terms, the YouTube privacy notice, or the Community Guidelines, please do not use the YouTube Website.

B. Although we may attempt to notify you when major changes are made to these Terms of Service, you should periodically review the most up-to-date version http://www.youtube.com/t/terms).
YouTube may, in its sole discretion, modify or revise these Terms of Service and policies at any time, and you agree to be bound by such modifications or revisions. Nothing in this Agreement shall be deemed to confer any third-party rights or benefits.

2. YouTube Website

A. These Terms of Service apply to all users of the YouTube Website, including users who are also contributors of video content, information, and other materials or services on the Website. The YouTube Website includes all aspects of YouTube, including but not limited to all products, software and services offered via the website such as the YouTube channels, the YouTube "Embeddable Player," the YouTube "Uploader" and other applications.

B. The YouTube Website may contain links to third party websites that are not owned or controlled by YouTube. YouTube has no control over, and assumes no responsibility for, the content, privacy policies, or practices of any third party websites. In addition, YouTube will not and cannot censor or edit the content of any third party site. By using the Website, you expressly relieve YouTube from any and all liability arising from your use of any third-party website.

C. Accordingly, we encourage you to be aware when you leave the YouTube Website and to read the terms and conditions and privacy policy of each other website that you visit.

3. YouTube Accounts

A. In order to access some features of the Website, you will have to create a YouTube account. You may never use another's account without permission. When creating your account, you must provide accurate and complete information. You are solely responsible for the activity that occurs on your account, and you must keep your account password secure. You must notify YouTube immediately of any breach of security or unauthorized use of your account.

B. Although YouTube will not be liable for your losses caused by any unauthorized use of your account, you may be liable for the losses of YouTube or others due to such unauthorized use.

4. General Use of the Website—Permissions and Restrictions

YouTube hereby grants you permission to access and use the Website as set forth in these Terms of Service, provided that:

A. You agree not to distribute in any medium any part of the Website, including but not limited to User Submissions (defined below), without YouTube's prior written authorization.

B. You agree not to alter or modify any part of the Website, including but not limited to YouTube's Embeddable Player or any of its related technologies.

C. You agree not to access User Submissions (defined below) or YouTube Content through any technology or means other than the video playback pages of the Website itself, the YouTube Embeddable Player, or other explicitly authorized means YouTube may designate.

D. You agree not to use the Website, including the YouTube Embeddable Player for any commercial use, without the prior written authorization of YouTube. Prohibited commercial uses include any of the following actions taken without YouTube's express approval:

- sale of access to the Website or its related services (such as the Embeddable Player) on another website;
- use of the Website or its related services (such as the Embeddable Player), for the primary purpose of gaining advertising or subscription revenue;
- the sale of advertising, on the YouTube website or any third-party website, targeted to the content of specific User Submissions or YouTube content;
- and any use of the Website or its related services (such as the Embeddable player) that YouTube finds, in its sole discretion, to use YouTube's resources or User Submissions with the effect of competing with or displacing the market for YouTube, YouTube content, or its User Submissions.

(For more information about prohibited commercial uses, see our FAQ.)

E. Prohibited commercial uses do not include:

- uploading an original video to YouTube, or maintaining an original channel on YouTube, to promote your business or artistic enterprise;
- using the Embeddable Player to show YouTube videos on an ad-enabled blog or website, provided the primary purpose of using the Embeddable Player is not to gain advertising revenue or compete with YouTube;
- any use that YouTube expressly authorizes in writing.

F. (For more information about what constitutes a prohibited commercial use, see our FAQ.)

G. If you use the YouTube Embeddable Player on your website, you must include a prominent link back to the YouTube website on the pages containing the Embeddable Player and you may not modify, build upon, or block any portion of the Embeddable Player in any way.

H. If you use the YouTube Uploader, you agree that it may automatically download and install updates from time to time from YouTube. These updates are designed to improve, enhance and further develop the Uploader and may take the form of bug fixes, enhanced functions, new software modules and completely new versions. You agree to receive such updates (and permit YouTube to deliver these to you) as part of your use of the Uploader.

I. You agree not to use or launch any automated system, including without limitation, "robots," "spiders," or "offline readers," that accesses the Website in a manner that sends more request messages to the YouTube servers in a given period of time than a human can reasonably produce in the same period by using a conventional on-line web browser. Notwithstanding the foregoing, YouTube grants the operators of public search engines permission to use spiders to copy materials from the site for the sole purpose of and solely to the extent necessary for creating publicly available searchable indices of the materials, but not caches or archives of such materials. YouTube reserves the right to revoke these exceptions either generally or in specific cases.
You agree not to collect or harvest any personally identifiable information, including account names, from the Website, nor to use the communication systems provided by the Website (e.g. comments, email) for any commercial solicitation purposes. You agree not to solicit, for commercial purposes, any users of the Website with respect to their User Submissions.

J. In your use of the website, you will otherwise comply with the terms and conditions of these Terms of Service, YouTube Community Guidelines, and all applicable local, national, and international laws and regulations.

K. YouTube reserves the right to discontinue any aspect of the YouTube Website at any time.

5. Your Use of Content on the Site

In addition to the general restrictions above, the following restrictions and conditions apply specifically to your use of content on the YouTube Website.

A. The content on the YouTube Website, except all User Submissions (as defined below), including without limitation, the text, software, scripts, graphics, photos, sounds, music, videos, interactive features and the like ("Content") and the trademarks, service marks and logos contained therein ("Marks"), are owned, by or licensed to YouTube, subject to copyright and other intellectual property rights under the law. Content on the Website is provided to you AS IS for your information and personal use only and may not be downloaded, copied, reproduced,

distributed, transmitted, broadcast, displayed, sold, licensed, or otherwise exploited for any other purposes whatsoever without the prior written consent of the respective owners. YouTube
reserves all rights not expressly granted in and to the Website and the Content.
B. You may access User Submissions for your information and personal use solely as intended through the provided functionality of the YouTube Website. You shall not copy or download any
User Submission unless you see a “download” or similar link displayed by YouTube on the YouTube Website for that User Submission.
C. User Comments are made available to you for your information and personal use solely as intended through the normal functionality of the YouTube Website. User Comments are made
available "as is", and may not be used, copied, reproduced, distributed, transmitted, broadcast, displayed, sold, licensed, downloaded, or otherwise exploited in any manner not intended by the
normal functionality of the YouTube Website or otherwise as prohibited under this Agreement.
D. You may access YouTube Content, User Submissions and other content only as permitted under this Agreement. YouTube reserves all rights not expressly granted in and to the YouTube
Content and the YouTube Website.
E. You agree to not engage in the use, copying, or distribution of any of the Content other than expressly permitted herein, including any use, copying, or distribution of User Submissions of
third parties obtained through the Website for any commercial purposes.
F. You agree not to circumvent, disable or otherwise interfere with security-related features of the YouTube Website or features that prevent or restrict use or copying of any Content or enforce
limitations on use of the YouTube Website or the Content therein.
G. You understand that when using the YouTube Website, you will be exposed to User Submissions from a variety of sources, and that YouTube is not responsible for the accuracy,
usefulness, safety, or intellectual property rights of or relating to such User Submissions. You further understand and acknowledge that you may be exposed to User Submissions that are
inaccurate, offensive, indecent, or objectionable, and you agree to waive, and hereby do waive, any legal or equitable rights or remedies you have or may have against YouTube with respect
thereto, and agree to indemnify and hold YouTube, its Owners/Operators, affiliates, and/or licensors, harmless to the fullest extent allowed by law regarding all matters related to your use of the
site.

6. Your User Submissions and Conduct

A. As a YouTube account holder you may submit video content ("User Videos") and textual content ("User Comments"). User Videos and User Comments are collectively referred to as "User
Submissions." You understand that whether or not such User Submissions are published, YouTube does not guarantee any confidentiality with respect to any User Submissions.
B. You shall be solely responsible for your own User Submissions and the consequences of posting or publishing them. In connection with User Submissions, you affirm, represent, and/or
warrant that: you own or have the necessary licenses, rights, consents, and permissions to use and authorize YouTube to use all patent, trademark, trade secret, copyright or other proprietary
rights in and to any and all User Submissions to enable inclusion and use of the User Submissions in the manner contemplated by the Website and these Terms of Service.
C. For clarity, you retain all of your ownership rights in your User Submissions. However, by submitting User Submissions to YouTube, you hereby grant YouTube a worldwide, non-exclusive,
royalty-free, sublicenseable and transferable license to use, reproduce, distribute, prepare derivative works of, display, and perform the User Submissions in connection with the YouTube
Website and YouTube's (and its successors' and affiliates') business, including without limitation for
promoting and redistributing part or all of the YouTube Website (and derivative works thereof) in any media formats and through any media channels. You also hereby grant each user of the
YouTube Website a non-exclusive license to access your User Submissions through the Website, and to use, reproduce, distribute, display and perform such User Submissions as permitted
through the functionality of the Website and under these Terms of Service. The above
licenses granted by you in User Videos terminate within a commercially reasonable time after you remove or delete your User Videos from the YouTube Website. You understand and agree,

however, that YouTube may retain, but not display, distribute, or perform, server copies of User Submissions that have been removed or deleted. The above licenses granted by you in User Comments are perpetual and irrevocable.
D. In connection with User Submissions, you further agree that you will not submit material that is copyrighted, protected by trade secret or otherwise subject to third party proprietary rights, including privacy and publicity rights, unless you are the owner of such rights or have permission from their rightful owner to post the material and to grant YouTube all of the license rights granted herein.
E. You further agree that you will not, in connection with User Submissions, submit material that is contrary to the YouTube Community Guidelines, found at http://www.youtube.com/t/community_guidelines, which may be updated from time to time, or contrary to applicable local, national, and international laws and regulations.
F. YouTube does not endorse any User Submission or any opinion, recommendation, or advice expressed therein, and YouTube expressly disclaims any and all liability in connection with User Submissions. YouTube does not permit copyright infringing activities and infringement of intellectual property rights on its Website, and YouTube will remove all Content and User Submissions if properly notified that such Content or User Submission infringes on another's intellectual property rights. YouTube reserves the right to remove Content and User Submissions without prior notice.

7. Account Termination Policy

A. YouTube will terminate a User's access to its Website if, under appropriate circumstances, they are determined to be a repeat infringer.
B. YouTube reserves the right to decide whether Content or a User Submission is appropriate and complies with these Terms of Service for violations other than copyright infringement, such as, but not limited to, pornography, obscene or defamatory material, or excessive length. YouTube may remove such User Submissions and/or terminate a User's access for uploading such material in violation of these Terms of Service at any time, without prior notice and at its sole discretion.

8. Digital Millennium Copyright Act

A. If you are a copyright owner or an agent thereof and believe that any User Submission or other content infringes upon your copyrights, you may submit a notification pursuant to the Digital Millennium Copyright Act ("DMCA") by providing our Copyright Agent with the following information in writing (see 17 U.S.C 512(c)(3) for further detail):
- A physical or electronic signature of a person authorized to act on behalf of the owner of an exclusive right that is allegedly infringed;
- Identification of the copyrighted work claimed to have been infringed, or, if multiple copyrighted works at a single online site are covered by a single notification, a representative list of such works at that site;
- Identification of the material that is claimed to be infringing or to be the subject of infringing activity and that is to be removed or access to which is to be disabled and information reasonably sufficient to permit the service provider to locate the material;
- Information reasonably sufficient to permit the service provider to contact you, such as an address, telephone number, and, if available, an electronic mail;
- A statement that you have a good faith belief that use of the material in the manner complained of is not authorized by the copyright owner, its agent, or the law; and
- A statement that the information in the notification is accurate, and under penalty of perjury, that you are authorized to act on behalf of the owner of an exclusive right that is allegedly infringed.

B. YouTube's designated Copyright Agent to receive notifications of claimed infringement is: Shadie Farazian, 901 Cherry Ave., San Bruno, CA 94066, email: copyright@youtube.com, fax: 650-872-8513. For clarity, only DMCA notices should go to the Copyright Agent; any other feedback, comments, requests for technical support, and other communications should be directed to YouTube customer service through http://www.google.com/support/youtube. You acknowledge that if you fail to comply with all of the requirements of this Section 5(D), your DMCA notice may not be valid.

C. Counter-Notice. If you believe that your User Submission that was removed (or to which access was disabled) is not infringing, or that you have the authorization from the copyright owner, the
copyright owner's agent, or pursuant to the law, to post and use the content in your User Submission, you may send a counter-notice containing the following information to the Copyright Agent:
- Your physical or electronic signature;
- Identification of the content that has been removed or to which access has been disabled and the location at which the content appeared before it was removed or disabled;
- A statement that you have a good faith belief that the content was removed or disabled as a result of mistake or a misidentification of the content; and Your name, address, telephone
number, and e-mail address, a statement that you consent to the jurisdiction of the federal court in San Francisco, California, and a statement that you will accept service of process from the
person who provided notification of the alleged infringement.
D. If a counter-notice is received by the Copyright Agent, YouTube may send a copy of the counter-notice to the original complaining party informing that person that it may replace the removed
content or cease disabling it in 10 business days. Unless the copyright owner files an action seeking a court order against the content provider, member or user, the removed content may be
replaced, or access to it restored, in 10 to 14 business days or more after receipt of the counter-notice, at YouTube's sole discretion.
9. Warranty Disclaimer
YOU AGREE THAT YOUR USE OF THE YOUTUBE WEBSITE SHALL BE AT YOUR SOLE RISK. TO THE FULLEST EXTENT PERMITTED BY LAW, YOUTUBE, ITS OFFICERS, DIRECTORS,
EMPLOYEES, AND AGENTS DISCLAIM ALL WARRANTIES, EXPRESS OR IMPLIED, IN CONNECTION WITH THE WEBSITE AND YOUR USE THEREOF. YOUTUBE MAKES NO WARRANTIES
OR REPRESENTATIONS ABOUT THE ACCURACY OR COMPLETENESS OF THIS SITE'S CONTENT OR THE CONTENT OF ANY SITES LINKED TO THIS SITE AND ASSUMES NO LIABILITY
OR RESPONSIBILITY FOR ANY (I) ERRORS, MISTAKES, OR INACCURACIES OF CONTENT, (II) PERSONAL INJURY OR PROPERTY DAMAGE, OF ANY NATURE WHATSOEVER,
RESULTING FROM YOUR ACCESS TO AND USE OF OUR WEBSITE, (III) ANY UNAUTHORIZED ACCESS TO OR USE OF OUR SECURE SERVERS AND/OR ANY AND ALL PERSONAL
INFORMATION AND/OR FINANCIAL INFORMATION STORED THEREIN, (IV) ANY INTERRUPTION OR CESSATION OF TRANSMISSION TO OR FROM OUR WEBSITE, (IV) ANY BUGS,
VIRUSES, TROJAN HORSES, OR THE LIKE WHICH MAY BE TRANSMITTED TO OR THROUGH OUR WEBSITE BY ANY THIRD PARTY, AND/OR (V) ANY ERRORS OR OMISSIONS IN ANY
CONTENT OR FOR ANY LOSS OR DAMAGE OF ANY KIND INCURRED AS A RESULT OF THE USE OF ANY CONTENT POSTED, EMAILED, TRANSMITTED, OR OTHERWISE MADE
AVAILABLE VIA THE YOUTUBE WEBSITE. YOUTUBE DOES NOT WARRANT, ENDORSE, GUARANTEE, OR ASSUME RESPONSIBILITY FOR ANY PRODUCT OR SERVICE ADVERTISED
OR OFFERED BY A THIRD PARTY THROUGH THE YOUTUBE WEBSITE OR ANY HYPERLINKED WEBSITE OR FEATURED IN ANY BANNER OR OTHER ADVERTISING, AND YOUTUBE
WILL NOT BE A PARTY TO OR IN ANY WAY BE RESPONSIBLE FOR MONITORING ANY TRANSACTION BETWEEN YOU AND THIRD-PARTY PROVIDERS OF PRODUCTS OR SERVICES.
AS WITH THE PURCHASE OF A PRODUCT OR SERVICE THROUGH ANY MEDIUM OR IN ANY ENVIRONMENT, YOU SHOULD USE YOUR BEST JUDGMENT AND EXERCISE CAUTION
WHERE APPROPRIATE.
10. Limitation of Liability
IN NO EVENT SHALL YOUTUBE, ITS OFFICERS, DIRECTORS, EMPLOYEES, OR AGENTS, BE LIABLE TO YOU FOR ANY DIRECT, INDIRECT, INCIDENTAL, SPECIAL, PUNITIVE, OR
CONSEQUENTIAL DAMAGES WHATSOEVER RESULTING FROM ANY (I) ERRORS, MISTAKES, OR INACCURACIES OF CONTENT, (II) PERSONAL INJURY OR PROPERTY DAMAGE, OF
ANY NATURE WHATSOEVER, RESULTING FROM YOUR ACCESS TO AND USE OF OUR WEBSITE, (III) ANY UNAUTHORIZED ACCESS TO OR USE OF OUR SECURE SERVERS AND/OR
ANY AND ALL PERSONAL INFORMATION AND/OR FINANCIAL INFORMATION STORED THEREIN, (IV) ANY INTERRUPTION OR CESSATION OF TRANSMISSION TO OR FROM OUR

WEBSITE, (IV) ANY BUGS, VIRUSES, TROJAN HORSES, OR THE LIKE, WHICH MAY BE TRANSMITTED TO OR THROUGH OUR WEBSITE BY ANY THIRD PARTY, AND/OR (V) ANY
ERRORS OR OMISSIONS IN ANY CONTENT OR FOR ANY LOSS OR DAMAGE OF ANY KIND INCURRED AS A RESULT OF YOUR USE OF ANY CONTENT POSTED, EMAILED,
TRANSMITTED, OR OTHERWISE MADE AVAILABLE VIA THE YOUTUBE WEBSITE, WHETHER BASED ON WARRANTY, CONTRACT, TORT, OR ANY OTHER LEGAL THEORY, AND
WHETHER OR NOT THE COMPANY IS ADVISED OF THE POSSIBILITY OF SUCH DAMAGES. THE FOREGOING LIMITATION OF LIABILITY SHALL APPLY TO THE FULLEST EXTENT
PERMITTED BY LAW IN THE APPLICABLE JURISDICTION.
YOU SPECIFICALLY ACKNOWLEDGE THAT YOUTUBE SHALL NOT BE LIABLE FOR USER SUBMISSIONS OR THE DEFAMATORY, OFFENSIVE, OR ILLEGAL CONDUCT OF ANY THIRD
PARTY AND THAT THE RISK OF HARM OR DAMAGE FROM THE FOREGOING RESTS ENTIRELY WITH YOU.
The Website is controlled and offered by YouTube from its facilities in the United States of America. YouTube makes no representations that the YouTube Website is appropriate or available for
use in other locations. Those who access or use the YouTube Website from other jurisdictions do so at their own volition and are responsible for compliance with local law.

11. Indemnity

You agree to defend, indemnify and hold harmless YouTube, its parent corporation, officers, directors, employees and agents, from and against any and all claims, damages, obligations, losses,
liabilities, costs or debt, and expenses (including but not limited to attorney's fees) arising from: (i) your use of and access to the YouTube Website; (ii) your violation of any term of these Terms of
Service; (iii) your violation of any third party right, including without limitation any copyright, property, or privacy right; or (iv) any claim that one of your User Submissions caused damage to a third
party. This defense and indemnification obligation will survive these Terms of Service and your use of the YouTube Website.

12. Ability to Accept Terms of Service

You affirm that you are either more than 18 years of age, or an emancipated minor, or possess legal parental or guardian consent, and are fully able and competent to enter into the terms,
conditions, obligations, affirmations, representations, and warranties set forth in these Terms of Service, and to abide by and comply with these Terms of Service. In any case, you affirm that you
are over the age of 13, as the YouTube Website is not intended for children under 13. If you are under 13 years of age, then please do not use the YouTube Website. There are lots of other
great web sites for you. Talk to your parents about what sites are appropriate for you.

13. Assignment

These Terms of Service, and any rights and licenses granted hereunder, may not be transferred or assigned by you, but may be assigned by YouTube without restriction.

14. General

You agree that: (i) the YouTube Website shall be deemed solely based in California; and (ii) the YouTube Website shall be deemed a passive website that does not give rise to personal
jurisdiction over YouTube, either specific or general, in jurisdictions other than California. These Terms of Service shall be governed by the internal substantive laws of the State of California,
without respect to its conflict of laws principles. Any claim or dispute between you and YouTube that arises in whole or in part from your use of the YouTube Website shall be decided exclusively
by a court of competent jurisdiction located in Santa Clara County, California. These Terms of Service, together with the Privacy Notice at http://www.youtube.com/t/privacy and any other legal
notices published by YouTube on the Website, shall constitute the entire agreement between you and YouTube concerning the YouTube Website. If any provision of these Terms of Service is
deemed invalid by a court of competent jurisdiction, the invalidity of such provision shall not affect the validity of the remaining provisions of these Terms of Service, which shall remain in full force
and effect. No waiver of any term of this these Terms of Service shall be deemed a further or continuing waiver of such term or any other term, and YouTube's failure to assert any right or
provision under these Terms of Service shall not constitute a waiver of such right or provision. YouTube reserves the right to amend these Terms of Service at any time and without notice, and it

is your responsibility to review these Terms of Service for any changes. Your use of the YouTube Website following any amendment of these Terms of Service will signify your assent to and
acceptance of its revised terms. YOU AND YOUTUBE AGREE THAT ANY CAUSE OF ACTION ARISING OUT OF OR RELATED TO THE YOUTUBE WEBSITE MUST COMMENCE WITHIN ONE
(1) YEAR AFTER THE CAUSE OF ACTION ACCRUES. OTHERWISE, SUCH CAUSE OF ACTION IS PERMANENTLY BARRED

TERMS AND CONDITIONS

A. ITUNES STORE, MAC APP STORE, APP STORE, AND IBOOKSTORE TERMS OF SALE
B. ITUNES STORE TERMS AND CONDITIONS
C. MAC APP STORE, APP STORE AND IBOOKSTORE TERMS AND CONDITIONS

THE LEGAL AGREEMENTS SET OUT BELOW GOVERN YOUR USE OF THE ITUNES STORE, MAC APP STORE, APP STORE, AND IBOOKSTORE SERVICES ("SERVICES"). TO AGREE TO THESE TERMS, CLICK "AGREE." IF YOU DO NOT AGREE TO THESE TERMS, DO NOT CLICK "AGREE," AND DO NOT USE THE SERVICES.

A. ITUNES STORE, MAC APP STORE, APP STORE, AND IBOOKSTORE TERMS OF SALE

PAYMENTS, TAXES, AND REFUND POLICY

You agree that you will pay for all products you purchase through the Services, and that Apple may charge your payment method for any products purchased and for any additional amounts (including any taxes and late fees, as applicable) that may be accrued by or in connection with your Account. YOU ARE RESPONSIBLE FOR THE TIMELY PAYMENT OF ALL FEES AND FOR PROVIDING APPLE WITH A VALID PAYMENT METHOD FOR PAYMENT OF ALL FEES. For details of how purchases are billed please visit support.apple.com/kb/HT5582.

Your total price will include the price of the product plus any applicable sales tax; such sales tax is based on the bill-to address and the sales tax rate in effect at the time you download the product. We will charge tax only in states where digital goods are taxable.

All sales and rentals of products are final.

Prices for products offered via the Services may change at any time, and the Services do not provide price protection or refunds in the event of a price reduction or promotional offering.

If a product becomes unavailable following a transaction but prior to download, your sole remedy is a refund. If technical problems prevent or unreasonably delay delivery of your product, your exclusive and sole remedy is either replacement or refund of the price paid, as determined by Apple.

1-Click®

1-Click is a registered service mark of Amazon.com, Inc., used under license. 1-Click is a convenient feature that allows you to make a purchase from the Services with a single click of your mouse or other input device. When accessing the Services on your computer, 1-Click purchasing may be activated via the dialog that appears when you click a Buy button. (You may reset this selection at any time by clicking Reset Warnings in your Account information). When accessing the Services on your Apple-branded products running iOS such as an iPad, iPod touch, or iPhone (“iOS Device”), 1-Click is activated for each transaction by tapping the button showing the price of the product, which reveals the Buy button. When 1-Click is activated, clicking or tapping the Buy button starts the download immediately and completes your transaction without any further steps.

GIFT CERTIFICATES, ITUNES CARDS, ALLOWANCES, AND CONTENT CODES

Gift Certificates, iTunes Cards, and Allowances are issued and managed by Apple Value Services, LLC ("Issuer").

Gift Certificates, iTunes Cards, Content Codes, and Allowances, in addition to unused balances, are not redeemable for cash and cannot be returned for a cash refund (except as required by law); exchanged; resold; used to purchase Gifts, Gift Certificates, or iTunes Cards; used to provide Allowances; used for purchases on the Apple Online Store; or used in Apple Retail Stores. Unused balances are not transferable.

Gift Certificates, iTunes Cards, Content Codes, and Allowances purchased in the United States may be redeemed through the Services only in the United States, its territories, and possessions.

The Gift Certificate/iTunes Card cash value is 1/10 of one cent.

Neither Issuer nor Apple is responsible for lost or stolen Gift Certificates, iTunes Cards, Content Codes, or Allowances. Risk of loss and title for Gift Certificates, iTunes Cards, and Allowances transmitted electronically pass to the purchaser in Virginia upon electronic transmission to the recipient. Risk of loss and title for Content Codes transmitted electronically pass in California upon electronic transmission from Apple; for avoidance of doubt, such recipient may not always be you.

Apple reserves the right to close accounts and request alternative forms of payment if a Gift Certificate, iTunes Card, Content Code, or Allowance is fraudulently obtained or used on the Service.

APPLE, ISSUER, AND THEIR LICENSEES, AFFILIATES, AND LICENSORS MAKE NO WARRANTIES, EXPRESS OR IMPLIED, WITH RESPECT TO GIFT CERTIFICATES, ITUNES CARDS, CONTENT CODES, ALLOWANCES, OR THE ITUNES STORE, APP STORE, MAC APP STORE, OR IBOOKSTORE, INCLUDING, WITHOUT LIMITATION, ANY EXPRESS OR IMPLIED WARRANTY OF MERCHANTABILITY OR FITNESS FOR A PARTICULAR PURPOSE. IN THE EVENT THAT A GIFT CERTIFICATE, ITUNES CARD, CONTENT CODE, OR ALLOWANCE IS NONFUNCTIONAL, YOUR SOLE REMEDY, AND OUR SOLE LIABILITY, SHALL BE THE REPLACEMENT OF SUCH GIFT CERTIFICATE, ITUNES CARD, CONTENT CODE, OR ALLOWANCE. THESE LIMITATIONS MAY NOT APPLY TO YOU. CERTAIN STATE LAWS DO NOT ALLOW LIMITATIONS ON IMPLIED WARRANTIES OR THE EXCLUSION OR LIMITATION OF CERTAIN DAMAGES. IF THESE LAWS APPLY TO YOU, SOME OR ALL OF THE ABOVE DISCLAIMERS, EXCLUSIONS, OR LIMITATIONS MAY NOT APPLY TO YOU, AND YOU MAY ALSO HAVE ADDITIONAL RIGHTS.

GIFTS

Gifts purchased from the Services may be purchased only for, and redeemed only by, persons in the United States, its territories, and possessions. Gift recipients must have compatible hardware and parental control settings to utilize some gifts.

PRE-ORDERS

By pre-ordering products, you are authorizing the Services to automatically charge your account and download the product when it becomes available. You may cancel your pre-order prior to the time the item becomes available.

ELECTRONIC CONTRACTING

Your use of the Services includes the ability to enter into agreements and/or to make transactions electronically. YOU ACKNOWLEDGE THAT YOUR ELECTRONIC SUBMISSIONS CONSTITUTE YOUR AGREEMENT AND INTENT TO BE BOUND BY AND TO PAY FOR SUCH AGREEMENTS AND TRANSACTIONS. YOUR AGREEMENT AND INTENT TO BE BOUND BY ELECTRONIC SUBMISSIONS APPLIES TO ALL RECORDS RELATING TO ALL TRANSACTIONS YOU ENTER INTO ON THIS SITE, INCLUDING NOTICES OF CANCELLATION, POLICIES, CONTRACTS, AND APPLICATIONS. In order to access and retain your electronic records, you may be required to have certain hardware and software, which are your sole responsibility.

Apple is not responsible for typographic errors.

B. ITUNES STORE TERMS AND CONDITIONS

THIS LEGAL AGREEMENT BETWEEN YOU AND APPLE INC. ("APPLE") GOVERNS YOUR USE OF THE ITUNES STORE SERVICE (THE “ITUNES SERVICE”).

THE ITUNES STORE SERVICE

Apple is the provider of the iTunes Service, which permits you to purchase or rent digital content ("iTunes Products") for end user use only under the terms and conditions set forth in this Agreement.

REQUIREMENTS FOR USE OF THE ITUNES SERVICE

This iTunes Service is available for individuals aged 13 years or older. If you are 13 or older but under the age of 18, you should review this Agreement with your parent or guardian to make sure that you and your parent or guardian understand it.

The iTunes Service is available to you only in the United States, its territories, and possessions. You agree not to use or attempt to use the iTunes Service from outside these locations. Apple may use technologies to verify your compliance.

Use of the iTunes Service requires compatible devices, Internet access, and certain software (fees may apply); may require periodic updates; and may be affected by the performance of these factors. High-speed Internet access is strongly recommended for regular use and is required for video. The latest version of required software is recommended to access the iTunes Service and may be required for certain transactions or features and to download iTunes Products previously purchased from the iTunes Service. You agree that meeting these requirements, which may change from time to time, is your responsibility. The iTunes Service is not part of any other product or offering, and no purchase or obtaining of any other product shall be construed to represent or guarantee you access to the iTunes Service.

YOUR ACCOUNT

As a registered user of the iTunes Service, you may establish an account ("Account"). Don’t reveal your Account information to anyone else. You are solely responsible for maintaining the confidentiality and security of your Account and for all activities that occur on or through your Account, and you agree to immediately notify Apple of any security breach of your Account. Apple shall not be responsible for any losses arising out of the unauthorized use of your Account.

In order to purchase and download iTunes Products from the iTunes Service, you must enter your Apple ID and password to authenticate your Account. Once you have authenticated your Account, you will not need to

authenticate again for fifteen minutes. During this time, you will be able to purchase and download iTunes Products without re-entering your password. You can turn off the ability to make iTunes Product purchases by adjusting the settings on your computer or iOS Device. For more information, please see http://support.apple.com/kb/HT1904 or http://support.apple.com/kb/HT4213.

You agree to provide accurate and complete information when you register with, and as you use, the iTunes Service ("iTunes Registration Data"), and you agree to update your iTunes Registration Data to keep it accurate and complete. You agree that Apple may store and use the iTunes Registration Data you provide for use in maintaining and billing fees to your Account.

AUTOMATIC DELIVERY AND DOWNLOADING PREVIOUS PURCHASES

When you first acquire music iTunes Products and music video iTunes Products (collectively, “iTunes Auto-Delivery Content”), you may elect to automatically receive (“auto-download”) copies of such iTunes Auto-Delivery Content on additional compatible iOS Devices and iTunes-authorized computers with compatible software by associating such iOS Devices and computers subject to the association rules below (each, an “Associated Device”). For each Associated Device, you may specify which type of iTunes Auto-Delivery Content, if any, may be auto-downloaded to it. On an Associated Device that is capable of receiving push notifications (“Push-Enabled”), including iOS Devices, the iTunes Auto-Delivery Content will auto-download to that Associated Device when it has an Internet connection; on an Associated Device that is not Push-Enabled, including those running on the Windows operating system, iTunes Auto-Delivery Content will automatically appear in the download queue and you may manually initiate the download within iTunes.

As an accommodation to you, subsequent to acquiring iTunes Auto-Delivery Content, purchased (i.e. not rented) movies iTunes Products and TV show iTunes Products (each, “iTunes Eligible Content”), you may download certain of such previously-purchased iTunes Eligible Content onto any Associated Device. Some iTunes Eligible Content that you previously purchased may not be available for subsequent download at any given time, and Apple shall have no liability to you in such event. As you may not be able to subsequently download certain previously-purchased iTunes Eligible Content, once you download an item of iTunes Eligible Content, it is your responsibility not to lose, destroy, or damage it, and you may want to back it up.

Association of Associated Devices is subject to the following terms:

(i) You may auto-download iTunes Auto-Delivery Content or download previously-purchased iTunes Eligible Content from an Account on up to 10 Associated Devices, provided no more than 5 are iTunes-authorized computers.

(ii) An Associated Device can be associated with only one Account at any given time.

(iii) You may switch an Associated Device to a different Account only once every 90 days.

(iv) You may download previously-purchased free content onto an unlimited number of devices while it is free on the iTunes Service, but on no more than 5 iTunes-authorized computers.

An Apple TV is not an “Associated Device.” However, TV show iTunes Products and purchased (i.e. not rented) movies iTunes Products may be played back on compatible Apple TVs, provided that you may only play back any such TV show or movie on a limited number of Apple TVs at the same time.

Some pieces of iTunes Eligible Content may be large, and significant data charges may result from delivery of such iTunes Eligible Content over a data connection.

ITUNES MATCH

iTunes Match permits you to remotely access your matched or uploaded songs, and music videos you have purchased with your Account, along with related metadata, playlists, and other information about your iTunes Library ("iTunes Match Content").

You may subscribe to iTunes Match for an annual fee. You must have a valid credit card on file with iTunes to subscribe. The subscription is non-refundable (except as required by applicable law), and will automatically renew for one-year periods until you cancel. Your account will be charged no more than 24 hours prior to the expiration of the current subscription period. You may cancel automatic renewal by adjusting the iTunes Store account settings on your computer. You will no longer be able to access your iTunes Match Content from iTunes Match after the end of your subscription period.

iTunes Match works with libraries that contain up to 25,000 songs which are either (i) not currently available on the iTunes Service, or (ii) not purchased from the iTunes Service with your Account. Songs that do not meet certain quality criteria or that are not authorized for your computer are not eligible for iTunes Match.

iTunes Match will automatically scan the song files and collect other information that may be used to identify media in your iTunes library, such as the names of songs, song artists or song durations. iTunes Match will use this information to match songs to those currently available on the iTunes Store, and will make matched songs available to you in a format then available on the iTunes Store. If the song is not successfully matched, your copy of the song will be uploaded to Apple in the same format or a format determined by Apple. Apple reserves the right to limit types of content uploaded (for example, excessively large files). Matched or uploaded songs and related metadata will be available for access from an Associated Device that has been enabled for iTunes Match. Association of Associated Devices for iTunes Match is subject to the same terms as Automatic Delivery and Downloading Previous Purchases, and uploaded or matched songs and related information are deemed to be "iTunes Eligible Content." You may also access iTunes Match Content from compatible Apple TVs, provided that you may only do so on a limited number of Apple TVs at the same time.

When you use iTunes Match, Genius will begin associating information about the media in your iTunes library with your Account; the association with your Account will continue for a period of time after your subscription ends. Apple will otherwise use this information as described in the Privacy Section of this Agreement. You will not be able to disable Genius while using iTunes Match, so if you prefer that we do not collect and use information from your iTunes library in this manner, you should not use iTunes Match.

You hereby agree to use iTunes Match only for lawfully acquired content. Any use for illegitimate content infringes the rights of others and may subject you to civil and criminal penalties, including possible monetary damages, for copyright infringement.

iTunes Match is provided on an "AS IS" basis and may contain errors or inaccuracies that could cause failures, corruption or loss of data and/or information, including music, playlist, and play history, from your computer or device and from peripherals (including, without limitation, servers and other computers) connected thereto. You should back up all data and information on your computer or device and any peripherals prior to using iTunes Match. You expressly acknowledge and agree that all use of iTunes Match is at your sole risk. To the extent permitted by law, Apple shall have no liability with respect to your use of iTunes Match, including the inability to access matched or uploaded content.

PRIVACY

The iTunes Service is subject to Apple's Privacy Policy at http://www.apple.com/privacy/.

When you opt in to the Genius feature, Apple will, from time to time, automatically collect information that can be used to identify media in your iTunes library on this computer, such as your play history and playlists. This includes media purchased through iTunes and media obtained from other sources. This information will be stored anonymously and will not be associated with your name or Account. When you use the Genius feature, Apple will use this information and the contents of your iTunes library, as well as other information, to give personalized recommendations to you.

Apple may only use this information and combine it with aggregated information from the iTunes libraries of other users who also opt in to this feature, your iTunes Store purchase history data, aggregated purchase history data from other iTunes Store users, and other information obtained from third parties, to:

• Create personalized playlists for you from your iTunes library.

• Provide you with recommendations regarding media and other products and services that you may wish to purchase.

• Provide recommendations regarding products and services to other users.

At all times your information will be treated in accordance with Apple's Privacy Policy.

Once you opt in to the Genius feature in iTunes, you will be able to create Genius playlists on Genius-capable devices. To enable the Genius feature on a device, you must sync it with your iTunes library after you have opted in.

If you prefer that we do not collect and use information from your iTunes library in this manner, you should not enable the Genius feature. You can revoke your opt-in choice at any time by turning off the Genius feature from the Store menu in iTunes on your computer. After you opt out, iTunes will no longer send information about your iTunes library to Apple. If you have elected to share your library from multiple computers, you need to turn off the Genius feature from each computer. The Genius feature cannot be enabled or disabled from your device.

By opting in to the Genius feature, you consent to the use of your information as described above and as described in Apple's Privacy Policy.

CONTENT AVAILABILITY

Apple reserves the right to change content options (including eligibility for particular features) without notice. For further information or concerns about closed captioning in specific content within the iTunes Store, please emailaccessibility@apple.com. You may also contact Thomas Montgomery, Accessibility Response Engineer, 1 Infinite Loop, Cupertino, California 95014, Phone/Fax: 408-783-5512.

USE OF PURCHASED OR RENTED CONTENT

You agree that the iTunes Service and certain iTunes Products include security technology that limits your use of iTunes Products and that, whether or not iTunes Products are limited by security technology, you shall use iTunes Products in compliance with the applicable usage rules established by Apple and its licensors ("Usage Rules"), and that any other use of the iTunes Products may constitute a copyright infringement. Any security technology is an inseparable part of the iTunes Products. Apple reserves the right to modify the Usage Rules at any time. You agree not to violate, circumvent, reverse-engineer, decompile, disassemble, or otherwise tamper with any of the security technology related to such Usage Rules for any reason—or to attempt or assist another

person to do so. Usage Rules may be controlled and monitored by Apple for compliance purposes, and Apple reserves the right to enforce the Usage Rules without notice to you. You agree not to access the iTunes Service by any means other than through software that is provided by Apple for accessing the iTunes Service. You shall not access or attempt to access an Account that you are not authorized to access. You agree not to modify the software in any manner or form, or to use modified versions of the software, for any purposes including obtaining unauthorized access to the iTunes Service. Violations of system or network security may result in civil or criminal liability.

USAGE RULES

(i) You shall be authorized to use iTunes Products only for personal, noncommercial use.

(ii) You shall be authorized to use iTunes Products on five iTunes-authorized devices at any time, except for Content Rentals (see below).

(iii) You shall be able to store iTunes Products from up to five different Accounts at a time on compatible devices, provided that each iPhone may sync tone iTunes Products with only a single iTunes-authorized device at a time, and syncing an iPhone with a different iTunes-authorized device will cause tone iTunes Products stored on that iPhone to be erased.

(iv) You shall be authorized to burn an audio playlist up to seven times.

(v) You shall not be entitled to burn video iTunes Products or tone iTunes Products.

(vi) iTunes Plus Products do not contain security technology that limits your usage of such products, and Usage Rules (ii) – (v) do not apply to iTunes Plus Products. You may copy, store, and burn iTunes Plus Products as reasonably necessary for personal, noncommercial use.

(vii) You shall be able to manually sync a movie from at least one iTunes-authorized device to devices that have manual sync mode, provided that the movie is associated with an Account on the primary iTunes-authorized device, where the primary iTunes-authorized device is the one that was first synced with the device or the one that you subsequently designate as primary using iTunes.

(viii) An HDCP connection is required to view content transmitted over HDMI.

(ix) Content Rentals

(a) Content rentals are viewable on only one device at a time. You must be connected to the iTunes Service when moving rentals, and you may do so only between your computer and other compatible devices. Content rented using your Apple TV, iPad, iPhone 4, or iPod touch (4th generation) may not be moved. If you move a rental to a compatible device and then use the iTunes Service to restore that device, or choose Settings > Reset > Erase all content and settings on that device, the rental will be permanently deleted.

(b) Once you purchase a rental, you must fully download the rental within thirty (30) days. You have thirty (30) days after downloading a rental to begin viewing. Once you begin viewing, you have twenty-four (24) hours to finish viewing a movie. Stopping, pausing, or restarting a rental does not extend the available time for viewing.

Some iTunes Products, including but not limited to Content rentals, may be downloaded only once and cannot be replaced if lost for any reason. It is your responsibility not to lose, destroy, or damage iTunes Products once downloaded, and you may wish to back them up.

The delivery of iTunes Products does not transfer to you any commercial or promotional use rights in the iTunes Products. Any burning or exporting capabilities are solely an accommodation to you and shall not constitute a grant, waiver, or other limitation of any rights of the copyright owners in any content embodied in any iTunes Product.

You acknowledge that, because some aspects of the iTunes Service, iTunes Products, and administration of the Usage Rules entails the ongoing involvement of Apple, if Apple changes any part of or discontinues the iTunes Service, which Apple may do at its election, you may not be able to use iTunes Products to the same extent as prior to such change or discontinuation, and that Apple shall have no liability to you in such case.

SEASON PASS, MULTI-PASS, ITUNES PASS

The full price of the Season Pass, Multi-Pass, or iTunes Pass is charged upon purchase. You must connect to the iTunes Service and download any remaining Pass content within 90 days after the final Pass content becomes available (or such other time period as may be specified on the purchase page), after which that content may no longer be available for download as part of the purchase. If automatic renewal is selected when you purchase a Multi-Pass, you will be charged the full price of each subsequent Multi-Pass cycle, unless and until you cancel automatic renewal prior to the beginning of the subsequent Multi-Pass cycle (in the Manage Passes section of your Account information). If a network or studio delivers fewer TV episodes than planned when you purchased a Season Pass, we will credit to your Account the retail value of the corresponding number of episodes.

HIGH-DEFINITION (HD) ITUNES PRODUCTS

HD iTunes Products are viewable only on HD capable devices; however, HD iTunes Products purchased (not rented) include a standard-definition version for use on non-HD devices.

SUBMISSIONS TO THE ITUNES SERVICE

The iTunes Service may offer interactive features that allow you to submit materials (including links to third-party content) on areas of the iTunes Service accessible and viewable by the public. You agree that any use by you of such features, including any materials submitted by you, shall be your sole responsibility, shall not infringe or violate the rights of any other party or violate any laws, contribute to or encourage infringing or otherwise unlawful conduct, or otherwise be obscene, objectionable, or in poor taste. You also agree that you have obtained all necessary rights and licenses. You agree to provide accurate and complete information in connection with your submission of any materials on the iTunes Service. You hereby grant Apple a worldwide, royalty-free, nonexclusive license to use such materials as part of the iTunes Service, and in relation to iTunes Products, without any compensation or obligation to you. Apple reserves the right to not post or publish any materials, and to remove or edit any material, at any time in its sole discretion without notice or liability.

Apple has the right, but not the obligation, to monitor any materials submitted by you or otherwise available on the iTunes Service, to investigate any reported or apparent violation of this Agreement, and to take any action that Apple in its sole discretion deems appropriate, including, without limitation, termination hereunder or under Apple's Copyright Policy (http://www.apple.com/legal/copyright.html).

THIRD-PARTY MATERIALS

Certain content, iTunes Products, and services available via the iTunes Service may include materials from third parties. Apple may provide links to third-party websites as a convenience to you. You agree that Apple is not responsible for examining or evaluating the content or accuracy and Apple does not warrant and will not have

any liability or responsibility for any third-party materials or websites, or for any other materials, products, or services of third parties. You agree that you will not use any third-party materials in a manner that would infringe or violate the rights of any other party and that Apple is not in any way responsible for any such use by you.

OBJECTIONABLE MATERIAL

You understand that by using the iTunes Service, you may encounter material that you may deem to be offensive, indecent, or objectionable, and that such content may or may not be identified as having explicit material. Nevertheless, you agree to use the iTunes Service at your sole risk and Apple shall have no liability to you for material that may be found to be offensive, indecent, or objectionable. iTunes Product types and descriptions are provided for convenience, and you agree that Apple does not guarantee their accuracy.

IMPORTANT SAFETY INFORMATION

To avoid muscle, joint, or eye strain during your use of the products offered through the iTunes Service, you should always take frequent breaks, and take a longer rest if you experience any soreness, fatigue, or discomfort. A very small percentage of people may experience seizures or blackouts when exposed to flashing lights or patterns, including but not limited to while playing video games or watching videos. Symptoms may include dizziness, nausea, involuntary movements, loss of awareness, altered vision, tingling, numbness, or other discomforts. Consult a doctor before using the products offered through the iTunes Service if you have ever suffered these or similar symptoms, and stop using such products immediately and see a doctor if they occur during your use of such products. Parents should monitor their children’s use of the products offered through the iTunes Service for signs of symptoms.

INTELLECTUAL PROPERTY

You agree that the iTunes Service, including but not limited to iTunes Products, graphics, user interface, audio clips, video clips, editorial content, and the scripts and software used to implement the iTunes Service, contains proprietary information and material that is owned by Apple and/or its licensors, and is protected by applicable intellectual property and other laws, including but not limited to copyright. You agree that you will not use such proprietary information or materials in any way whatsoever except for use of the iTunes Service in compliance with this Agreement. No portion of the iTunes Service may be reproduced in any form or by any means, except as expressly permitted in these terms. You agree not to modify, rent, lease, loan, sell, distribute, or create derivative works based on the iTunes Service in any manner, and you shall not exploit the iTunes Service in any unauthorized way whatsoever, including, but not limited to, by trespass or burdening network capacity.

Notwithstanding any other provision of this Agreement, Apple and its licensors reserve the right to change, suspend, remove, or disable access to any iTunes Products, content, or other materials comprising a part of the iTunes Service at any time without notice. In no event will Apple be liable for making these changes. Apple may also impose limits on the use of or access to certain features or portions of the iTunes Service, in any case and without notice or liability.

All copyrights in and to the iTunes Service (including the compilation of content, postings, links to other Internet resources, and descriptions of those resources) and related software are owned by Apple and/or its licensors, who reserve all their rights in law and equity. THE USE OF THE SOFTWARE OR ANY PART OF THE ITUNES SERVICE, EXCEPT FOR USE OF THE ITUNES SERVICE AS PERMITTED IN THIS AGREEMENT, IS STRICTLY PROHIBITED AND INFRINGES ON THE INTELLECTUAL PROPERTY RIGHTS OF OTHERS AND MAY SUBJECT YOU TO CIVIL AND CRIMINAL PENALTIES, INCLUDING POSSIBLE MONETARY DAMAGES, FOR COPYRIGHT INFRINGEMENT.

Apple, the Apple logo, iTunes, and other Apple trademarks, service marks, graphics, and logos used in connection with the iTunes Service are trademarks or registered trademarks of Apple Inc. in the U.S. and/or other countries. Other trademarks, service marks, graphics, and logos used in connection with the iTunes Service may be the trademarks of their respective owners. You are granted no right or license with respect to any of the aforesaid trademarks and any use of such trademarks.

As an Account holder of the iTunes Service in good standing, you may be provided with limited access to download certain album cover art for music stored in the iTunes Library of your iTunes application. Such access is provided as an accommodation only, and Apple does not warrant, and will not have any liability or responsibility for, such album cover art or your use thereof. You may access album cover art only for music for which you are the lawful owner of a legal copy. Album cover art is provided for personal, noncommercial use only. You agree that you will not use album cover art in any manner that would infringe or violate this Agreement or the rights of any other party, and that Apple is not in any way responsible for any such use by you.

TERMINATION

If you fail, or Apple suspects that you have failed, to comply with any of the provisions of this Agreement, Apple, at its sole discretion, without notice to you may: (i) terminate this Agreement and/or your Account, and you will remain liable for all amounts due under your Account up to and including the date of termination; and/or (ii) terminate the license to the software; and/or (iii) preclude access to the iTunes Service (or any part thereof).

Apple reserves the right to modify, suspend, or discontinue the iTunes Service (or any part or content thereof) at any time with or without notice to you, and Apple will not be liable to you or to any third party should it exercise such rights.

DISCLAIMER OF WARRANTIES; LIABILITY LIMITATION

APPLE DOES NOT GUARANTEE, REPRESENT, OR WARRANT THAT YOUR USE OF THE ITUNES SERVICE WILL BE UNINTERRUPTED OR ERROR-FREE, AND YOU AGREE THAT FROM TIME TO TIME APPLE MAY REMOVE THE ITUNES SERVICE FOR INDEFINITE PERIODS OF TIME, OR CANCEL THE ITUNES SERVICE AT ANY TIME, WITHOUT NOTICE TO YOU.

YOU EXPRESSLY AGREE THAT YOUR USE OF, OR INABILITY TO USE, THE ITUNES SERVICE IS AT YOUR SOLE RISK. THE ITUNES SERVICE AND ALL PRODUCTS AND SERVICES DELIVERED TO YOU THROUGH THE ITUNES SERVICE ARE (EXCEPT AS EXPRESSLY STATED BY APPLE) PROVIDED "AS IS" AND "AS AVAILABLE" FOR YOUR USE, WITHOUT WARRANTIES OF ANY KIND, EITHER EXPRESS OR IMPLIED, INCLUDING ALL IMPLIED WARRANTIES OF MERCHANTABILITY, FITNESS FOR A PARTICULAR PURPOSE, TITLE, AND NONINFRINGEMENT. BECAUSE SOME JURISDICTIONS DO NOT ALLOW THE EXCLUSION OF IMPLIED WARRANTIES, THE ABOVE EXCLUSION OF IMPLIED WARRANTIES MAY NOT APPLY TO YOU.

IN NO CASE SHALL APPLE, ITS DIRECTORS, OFFICERS, EMPLOYEES, AFFILIATES, AGENTS, CONTRACTORS, OR LICENSORS BE LIABLE FOR ANY DIRECT, INDIRECT, INCIDENTAL, PUNITIVE, SPECIAL, OR CONSEQUENTIAL DAMAGES ARISING FROM YOUR USE OF ANY OF THE ITUNES SERVICE OR FOR ANY OTHER CLAIM RELATED IN ANY WAY TO YOUR USE OF THE ITUNES SERVICE, INCLUDING, BUT NOT LIMITED TO, ANY ERRORS OR OMISSIONS IN ANY CONTENT, OR ANY LOSS OR DAMAGE OF ANY KIND INCURRED AS A RESULT OF THE USE OF ANY CONTENT (OR PRODUCT) POSTED, TRANSMITTED, OR OTHERWISE MADE AVAILABLE VIA

THE ITUNES SERVICE, EVEN IF ADVISED OF THEIR POSSIBILITY. BECAUSE SOME STATES OR JURISDICTIONS DO NOT ALLOW THE EXCLUSION OR THE LIMITATION OF LIABILITY FOR CONSEQUENTIAL OR INCIDENTAL DAMAGES, IN SUCH STATES OR JURISDICTIONS, APPLE'S LIABILITY SHALL BE LIMITED TO THE EXTENT PERMITTED BY LAW.

APPLE SHALL USE REASONABLE EFFORTS TO PROTECT INFORMATION SUBMITTED BY YOU IN CONNECTION WITH THE ITUNES SERVICE, BUT YOU AGREE THAT YOUR SUBMISSION OF SUCH INFORMATION IS AT YOUR SOLE RISK, AND APPLE HEREBY DISCLAIMS ANY AND ALL LIABILITY TO YOU FOR ANY LOSS OR LIABILITY RELATING TO SUCH INFORMATION IN ANY WAY.

APPLE DOES NOT REPRESENT OR GUARANTEE THAT THE ITUNES SERVICE WILL BE FREE FROM LOSS, CORRUPTION, ATTACK, VIRUSES, INTERFERENCE, HACKING, OR OTHER SECURITY INTRUSION, AND APPLE DISCLAIMS ANY LIABILITY RELATING THERETO. SOME PRODUCTS CAN BE DOWNLOADED ONLY ONCE; AFTER BEING DOWNLOADED, THEY CANNOT BE REPLACED IF LOST FOR ANY REASON. YOU SHALL BE RESPONSIBLE FOR BACKING UP YOUR OWN SYSTEM, INCLUDING ANY ITUNES PRODUCTS PURCHASED OR RENTED FROM THE ITUNES STORE.

WAIVER AND INDEMNITY

BY USING THE ITUNES SERVICE, YOU AGREE, TO THE EXTENT PERMITTED BY LAW, TO INDEMNIFY AND HOLD APPLE, ITS DIRECTORS, OFFICERS, EMPLOYEES, AFFILIATES, AGENTS, CONTRACTORS, AND LICENSORS HARMLESS WITH RESPECT TO ANY CLAIMS ARISING OUT OF YOUR BREACH OF THIS AGREEMENT, YOUR USE OF THE ITUNES SERVICE, OR ANY ACTION TAKEN BY APPLE AS PART OF ITS INVESTIGATION OF A SUSPECTED VIOLATION OF THIS AGREEMENT OR AS A RESULT OF ITS FINDING OR DECISION THAT A VIOLATION OF THIS AGREEMENT HAS OCCURRED. THIS MEANS THAT YOU CANNOT SUE OR RECOVER ANY DAMAGES FROM APPLE, ITS DIRECTORS, OFFICERS, EMPLOYEES, AFFILIATES, AGENTS, CONTRACTORS, AND LICENSORS AS A RESULT OF ITS DECISION TO REMOVE OR REFUSE TO PROCESS ANY INFORMATION OR CONTENT, TO WARN YOU, TO SUSPEND OR TERMINATE YOUR ACCESS TO THE ITUNES SERVICE, OR TO TAKE ANY OTHER ACTION DURING THE INVESTIGATION OF A SUSPECTED VIOLATION OR AS A RESULT OF APPLE'S CONCLUSION THAT A VIOLATION OF THIS AGREEMENT HAS OCCURRED. THIS WAIVER AND INDEMNITY PROVISION APPLIES TO ALL VIOLATIONS DESCRIBED IN OR CONTEMPLATED BY THIS AGREEMENT.

CHANGES

Apple reserves the right at any time to modify this Agreement and to impose new or additional terms or conditions on your use of the iTunes Service. Such modifications and additional terms and conditions will be effective immediately and incorporated into this Agreement. Your continued use of the iTunes Service will be deemed acceptance thereof.

MISCELLANEOUS

This Agreement constitutes the entire agreement between you and Apple and governs your use of the iTunes Service, superseding any prior agreements between you and Apple. You also may be subject to additional terms and conditions that may apply when you use affiliate services, third-party content, or third-party software. If any part of this Agreement is held invalid or unenforceable, that portion shall be construed in a manner

consistent with applicable law to reflect, as nearly as possible, the original intentions of the parties, and the remaining portions shall remain in full force and effect. Apple's failure to enforce any right or provisions in this Agreement will not constitute a waiver of such or any other provision. Apple will not be responsible for failures to fulfill any obligations due to causes beyond its control.

The iTunes Service is operated by Apple from its offices in the United States. You agree to comply with all local, state, federal, and national laws, statutes, ordinances, and regulations that apply to your use of the iTunes Service. All transactions on the iTunes Service are governed by California law, without giving effect to its conflict of law provisions. Your use of the iTunes Service may also be subject to other laws. You expressly agree that exclusive jurisdiction for any claim or dispute with Apple or relating in any way to your use of the iTunes Service resides in the courts in the State of California. Risk of loss and title for all electronically delivered transactions pass to the purchaser in California upon electronic transmission to the recipient. No Apple employee or agent has the authority to vary this Agreement.

Apple may notify you with respect to the iTunes Service by sending an email message to your Account email address or a letter via postal mail to your Account mailing address, or by a posting on the iTunes Service. Notices shall become effective immediately.

Apple reserves the right to take steps Apple believes are reasonably necessary or appropriate to enforce and/or verify compliance with any part of this Agreement. You agree that Apple has the right, without liability to you, to disclose any Registration Data and/or Account information to law enforcement authorities, government officials, and/or a third party, as Apple believes is reasonably necessary or appropriate to enforce and/or verify compliance with any part of this Agreement (including but not limited to Apple's right to cooperate with any legal process relating to your use of the iTunes Service and/or iTunes Products, and/or a third-party claim that your use of the iTunes Service and/or iTunes Products is unlawful and/or infringes such third party's rights).

C. MAC APP STORE, APP STORE AND IBOOKSTORE TERMS AND CONDITIONS

THIS LEGAL AGREEMENT BETWEEN YOU AND APPLE INC. ("APPLE") GOVERNS YOUR USE OF THE MAC APP STORE, APP STORE AND IBOOKSTORE SERVICES (THE “APP AND BOOK SERVICES”).

THE MAC APP STORE, APP STORE AND IBOOKSTORE SERVICES

Apple is the provider of the App and Book Services that permit you to license software products and digital content (the “App and Book Products”) for end user use only under the terms and conditions set forth in this Agreement. For App Store Products (defined below), end users may be individuals acting in their own capacities, commercial enterprises or educational institutions.

REQUIREMENTS FOR USE OF THE APP AND BOOK SERVICES

These App and Book Services are available for individuals aged 13 years or older. If you are 13 or older but under the age of 18, you should review this Agreement with your parent or guardian to make sure that you and your parent or guardian understand it.

The App and Book Services are available to you only in the United States, its territories, and possessions. You agree not to use or attempt to use the App and Book Services from outside these locations. Apple may use technologies to verify your compliance.

Use of the App and Book Services requires compatible devices, Internet access, and certain software (fees may apply); may require periodic updates; and may be affected by the performance of these factors. High-speed Internet access is strongly recommended. The latest version of required software (including, but not limited to iTunes and/or Mac App Store software) is recommended to access the App and Book Services and may be required for certain transactions or features and to download App and Book Products previously purchased from the App and Book Services. You agree that meeting these requirements, which may change from time to time, is your responsibility. The App and Book Services are not part of any other product or offering, and no purchase or obtaining of any other product shall be construed to represent or guarantee you access to the App and Book Services.

YOUR ACCOUNT

As a registered user of the App and Book Services, you may establish an account ("Account"). Don’t reveal your Account information to anyone else. You are solely responsible for maintaining the confidentiality and security of your Account, and for all activities that occur on or through your Account, and you agree to immediately notify Apple of any security breach of your Account. Apple shall not be responsible for any losses arising out of the unauthorized use of your Account.

In order to purchase and download App and Book Products from the App and Book Services, you must enter your Apple ID and password to authenticate your Account. Once you have authenticated your Account, you will not need to authenticate again for fifteen minutes. During this time, you will be able to purchase and download App and Book Products without re-entering your password. You can turn off the ability to make App and Book Product purchases by adjusting the settings on your computer or iOS Device. For more information, please see http://support.apple.com/kb/HT1904 or http://support.apple.com/kb/HT4213.

You agree to provide accurate and complete information when you register with, and as you use, the App and Book Services ("App and Book Registration Data"), and you agree to update your App and Book Registration Data to keep it accurate and complete. You agree that Apple may store and use the App and Book Registration Data you provide for use in maintaining and billing fees to your Account.

AUTOMATIC DELIVERY AND DOWNLOADING PREVIOUS PURCHASES

When you first acquire App Store Products, as defined below, (excluding products acquired from the Mac App Store) or iBookstore Products, as defined below, through the App and Book Services (collectively, “Eligible Content”), you may elect to automatically receive (“auto-download”) copies of such Eligible Content on additional compatible iOS Devices and iTunes-authorized computers with compatible software by associating such iOS Devices and computers subject to the association rules below (each, an “Associated Device”). For each Associated Device, you may specify which type of Eligible Content, if any, may be auto-downloaded to it. On an Associated Device that is capable of receiving push notifications (“Push-Enabled”), including iOS Devices, the Eligible Content will auto-download to that Associated Device when it has an Internet connection; on an Associated Device that is not Push-Enabled, including those running on the Windows operating system, Eligible Content will automatically appear in the download queue and you may manually initiate the download within iTunes.

As an accommodation to you, subsequent to acquiring Eligible Content, you may download certain of such previously-purchased Eligible Content onto any Associated Device. Some Eligible Content that you previously purchased may not be available for subsequent download at any given time, and Apple shall have no liability to you in such event. As you may not be able to subsequently download certain previously-purchased Eligible Content, once you download an item of Eligible Content, it is your responsibility not to lose, destroy, or damage it, and you may want to back it up.

Association of Associated Devices is subject to the following terms:

(i) You may auto-download Eligible Content or download previously-purchased Eligible Content from an Account on up to 10 Associated Devices, provided no more than 5 are iTunes-authorized computers.

(ii) An Associated Device can be associated with only one Account at any given time.

(iii) You may switch an Associated Device to a different Account only once every 90 days.

(iv) You may download previously-purchased free content onto an unlimited number of devices while it is free on the App and Book Services, but on no more than 5 iTunes-authorized computers.

The above terms (i) to (iv) do not apply to App Store Products.

Some pieces of Eligible Content may be large, and significant data charges may result from delivery of such Eligible Content over a data connection.

PRIVACY

The App and Book Services are subject to Apple's Privacy Policy at http://www.apple.com/privacy/.

USE OF APP AND BOOK PRODUCTS AND THE APP AND BOOK SERVICES

You agree that the App and Book Services and certain App and Book Products include security technology that limits your use of App and Book Products and that, whether or not App and Book Products are limited by security technology, you shall use App and Book Products in compliance with the applicable usage rules established by Apple and its principals (“Usage Rules”), and that any other use of the App and Book Products may constitute a copyright infringement. Any security technology is an inseparable part of the App and Book Products. Apple reserves the right to modify the Usage Rules at any time. You agree not to violate, circumvent, reverse-engineer, decompile, disassemble, or otherwise tamper with any of the security technology related to such Usage Rules for any reason—or to attempt or assist another person to do so. Usage Rules may be controlled and monitored by Apple for compliance purposes, and Apple reserves the right to enforce the Usage Rules without notice to you. You agree not to access the App and Book Services by any means other than through software that is provided by Apple for accessing the App and Book Services. You shall not access or attempt to access an Account that you are not authorized to access. You agree not to modify the software in any manner or form, or to use modified versions of the software, for any purposes including obtaining unauthorized access to the App and Book Services. Violations of system or network security may result in civil or criminal liability.

The delivery of App and Book Products does not transfer to you any promotional use rights in the App and Book Products.

You acknowledge that, because some aspects of the App and Book Services, App and Book Products, and administration of the Usage Rules entails the ongoing involvement of Apple, if Apple changes any part of or discontinues the App and Book Services, which Apple may do at its election, you may not be able to use App and Book Products to the same extent as prior to such change or discontinuation, and that Apple shall have no liability to you in such case.

SUBMISSIONS TO THE APP AND BOOK SERVICES

The App and Book Services may offer interactive features that allow you to submit materials (including links to third-party content) on areas of the App and Book Services accessible and viewable by other users of the App and Book Services and the public. You agree that any use by you of such features, including any materials submitted by you, shall be your sole responsibility, shall not infringe or violate the rights of any other party or violate any laws, contribute to or encourage infringing or otherwise unlawful conduct, or otherwise be obscene, objectionable, or in poor taste. You also agree that you have obtained all necessary rights and licenses. You agree to provide accurate and complete information in connection with your submission of any materials on the App and Book Services. You hereby grant Apple a worldwide, royalty-free, nonexclusive license to use such materials as part of the App and Book Services, and in relation to App and Book Products, without any compensation or obligation to you. Apple reserves the right to not post or publish any materials, and to remove or edit any material, at any time in its sole discretion without notice or liability. You may not submit reviews or ratings for App Store Products downloaded using a promotional Content Code.

Apple has the right, but not the obligation, to monitor any materials submitted by you or otherwise available on the App and Book Services, to investigate any reported or apparent violation of this Agreement, and to take any action that Apple in its sole discretion deems appropriate, including, without limitation, termination hereunder or under Apple's Copyright Policy (http://www.apple.com/legal/copyright.html).

THIRD-PARTY MATERIALS

Certain content, App and Book Products, and services available via the App and Book Services may include materials from third parties. Apple may provide links to third-party websites as a convenience to you. You agree that Apple is not responsible for examining or evaluating the content or accuracy and Apple does not warrant and will not have any liability or responsibility for any third-party materials or websites, or for any other materials, products, or services of third parties. You agree that you will not use any third-party materials in a manner that would infringe or violate the rights of any other party and that Apple is not in any way responsible for any such use by you.

OBJECTIONABLE MATERIAL

You understand that by using the App and Book Services, you may encounter material that you may deem to be offensive, indecent, or objectionable, and that such content may or may not be identified as having explicit material. Nevertheless, you agree to use the App and Book Services at your sole risk and Apple shall have no liability to you for material that may be found to be offensive, indecent, or objectionable. App and Book Product types and descriptions are provided for convenience, and you agree that Apple does not guarantee their accuracy.

INTELLECTUAL PROPERTY

You agree that the App and Book Services, including but not limited to App and Book Products, graphics, user interface, audio clips, editorial content, and the scripts and software used to implement the App and Book Services, contain proprietary information and material that is owned by Apple and/or its principals, and is protected by applicable intellectual property and other laws, including but not limited to copyright. You agree that you will not use such proprietary information or materials in any way whatsoever except for use of the App and Book Services in compliance with this Agreement. No portion of the App and Book Services may be reproduced in any form or by any means, except as expressly permitted in these terms. You agree not to modify, rent, lease, loan, sell, distribute, or create derivative works based on the App and Book Services in any manner, and you shall not exploit the App and Book Services in any unauthorized way whatsoever, including, but not limited to, by trespass or burdening network capacity.

Notwithstanding any other provision of this Agreement, Apple and its principals reserve the right to change, suspend, remove, or disable access to any App and Book Products, content, or other materials comprising a part of the App and Book Services at any time without notice. In no event will Apple be liable for making these changes. Apple may also impose limits on the use of or access to certain features or portions of the App and Book Services, in any case and without notice or liability.

All copyrights in and to the App and Book Services (including the compilation of content, postings, links to other Internet resources, and descriptions of those resources) and related software are owned by Apple and/or its principals, who reserve all their rights in law and equity. THE USE OF THE SOFTWARE OR ANY PART OF THE APP AND BOOK SERVICES, EXCEPT FOR USE OF THE APP AND BOOK SERVICES AS PERMITTED IN THIS AGREEMENT, IS STRICTLY PROHIBITED AND INFRINGES ON THE INTELLECTUAL PROPERTY RIGHTS OF OTHERS AND MAY SUBJECT YOU TO CIVIL AND CRIMINAL PENALTIES, INCLUDING POSSIBLE MONETARY DAMAGES, FOR COPYRIGHT INFRINGEMENT.

Apple, the Apple logo, iTunes, App Store, and other Apple trademarks, service marks, graphics, and logos used in connection with the App and Book Services are trademarks or registered trademarks of Apple Inc. in the U.S. and/or other countries. Other trademarks, service marks, graphics, and logos used in connection with the App and Book Services may be the trademarks of their respective owners. You are granted no right or license with respect to any of the aforesaid trademarks and any use of such trademarks.

TERMINATION

If you fail, or Apple suspects that you have failed, to comply with any of the provisions of this Agreement, Apple, at its sole discretion, without notice to you may: (i) terminate this Agreement and/or your Account, and you will remain liable for all amounts due under your Account up to and including the date of termination; and/or (ii) terminate the license to the software; and/or (iii) preclude access to the App and Book Services (or any part thereof).

Apple reserves the right to modify, suspend, or discontinue the App and Book Services (or any part or content thereof) at any time with or without notice to you, and Apple will not be liable to you or to any third party should it exercise such rights.

DISCLAIMER OF WARRANTIES; LIABILITY LIMITATIONS

APPLE DOES NOT GUARANTEE, REPRESENT, OR WARRANT THAT YOUR USE OF THE APP AND BOOK SERVICES WILL BE UNINTERRUPTED OR ERROR-FREE, AND YOU AGREE THAT FROM TIME TO TIME APPLE MAY REMOVE THE APP AND BOOK SERVICES FOR INDEFINITE PERIODS OF TIME, OR CANCEL THE APP AND BOOK SERVICES AT ANY TIME, WITHOUT NOTICE TO YOU.

YOU EXPRESSLY AGREE THAT YOUR USE OF, OR INABILITY TO USE, THE APP AND BOOK SERVICES IS AT YOUR SOLE RISK. THE APP AND BOOK SERVICES AND ALL PRODUCTS AND APP AND BOOK SERVICES DELIVERED TO YOU THROUGH THE APP AND BOOK SERVICES ARE (EXCEPT AS EXPRESSLY STATED BY APPLE) PROVIDED "AS IS" AND "AS AVAILABLE" FOR YOUR USE, WITHOUT WARRANTIES OF ANY KIND, EITHER EXPRESS OR IMPLIED, INCLUDING ALL IMPLIED WARRANTIES OF MERCHANTABILITY, FITNESS FOR A PARTICULAR PURPOSE, TITLE, AND NONINFRINGEMENT. BECAUSE SOME JURISDICTIONS DO NOT ALLOW THE EXCLUSION OF IMPLIED WARRANTIES, THE ABOVE EXCLUSION OF IMPLIED WARRANTIES MAY NOT APPLY TO YOU.

IN NO CASE SHALL APPLE, ITS DIRECTORS, OFFICERS, EMPLOYEES, AFFILIATES, AGENTS, CONTRACTORS, PRINCIPALS, OR LICENSORS BE LIABLE FOR ANY DIRECT, INDIRECT, INCIDENTAL, PUNITIVE, SPECIAL, OR CONSEQUENTIAL DAMAGES ARISING FROM YOUR USE OF ANY OF THE APP AND BOOK SERVICES OR FOR ANY OTHER CLAIM RELATED IN ANY WAY TO YOUR USE OF THE APP AND BOOK SERVICES, INCLUDING, BUT NOT LIMITED TO, ANY ERRORS OR OMISSIONS IN ANY CONTENT, OR ANY LOSS OR DAMAGE OF ANY KIND INCURRED AS A RESULT OF THE USE OF ANY CONTENT (OR PRODUCT) POSTED, TRANSMITTED, OR OTHERWISE MADE AVAILABLE VIA THE APP AND BOOK SERVICES, EVEN IF ADVISED OF THEIR POSSIBILITY. BECAUSE SOME STATES OR JURISDICTIONS DO NOT ALLOW THE EXCLUSION OR THE LIMITATION OF LIABILITY FOR CONSEQUENTIAL OR INCIDENTAL DAMAGES, IN SUCH STATES OR JURISDICTIONS, APPLE'S LIABILITY SHALL BE LIMITED TO THE EXTENT PERMITTED BY LAW.

APPLE SHALL USE REASONABLE EFFORTS TO PROTECT INFORMATION SUBMITTED BY YOU IN CONNECTION WITH THE APP AND BOOK SERVICES, BUT YOU AGREE THAT YOUR SUBMISSION OF SUCH INFORMATION IS AT YOUR SOLE RISK, AND APPLE HEREBY DISCLAIMS ANY AND ALL LIABILITY TO YOU FOR ANY LOSS OR LIABILITY RELATING TO SUCH INFORMATION IN ANY WAY.

APPLE DOES NOT REPRESENT OR GUARANTEE THAT THE APP AND BOOK SERVICES WILL BE FREE FROM LOSS, CORRUPTION, ATTACK, VIRUSES, INTERFERENCE, HACKING, OR OTHER SECURITY INTRUSION, AND APPLE DISCLAIMS ANY LIABILITY RELATING THERETO. YOU SHALL BE RESPONSIBLE FOR BACKING UP YOUR OWN SYSTEM, INCLUDING ANY APP AND BOOK PRODUCTS PURCHASED FROM THE APP AND BOOK SERVICES.

WAIVER AND INDEMNITY

BY USING THE APP AND BOOK SERVICES, YOU AGREE, TO THE EXTENT PERMITTED BY LAW, TO INDEMNIFY AND HOLD APPLE, ITS DIRECTORS, OFFICERS, EMPLOYEES, AFFILIATES, AGENTS, CONTRACTORS, PRINCIPALS, AND LICENSORS HARMLESS WITH RESPECT TO ANY CLAIMS ARISING OUT OF YOUR BREACH OF THIS AGREEMENT, YOUR USE OF THE APP AND BOOK SERVICES, OR ANY ACTION TAKEN BY APPLE AS PART OF ITS INVESTIGATION OF A SUSPECTED VIOLATION OF THIS AGREEMENT OR AS A RESULT OF ITS FINDING OR DECISION THAT A VIOLATION OF THIS AGREEMENT HAS OCCURRED. THIS MEANS THAT YOU CANNOT SUE OR RECOVER ANY DAMAGES FROM APPLE, ITS DIRECTORS, OFFICERS, EMPLOYEES, AFFILIATES, AGENTS, CONTRACTORS, PRINCIPALS, AND LICENSORS AS A RESULT OF ITS DECISION TO REMOVE OR REFUSE TO PROCESS ANY INFORMATION OR CONTENT, TO WARN YOU, TO SUSPEND OR TERMINATE YOUR ACCESS TO THE APP AND BOOK SERVICES, OR TO TAKE ANY OTHER ACTION DURING THE INVESTIGATION OF A SUSPECTED VIOLATION OR AS A RESULT OF APPLE'S CONCLUSION THAT A VIOLATION OF THIS AGREEMENT HAS OCCURRED. THIS WAIVER AND INDEMNITY PROVISION APPLIES TO ALL VIOLATIONS DESCRIBED IN OR CONTEMPLATED BY THIS AGREEMENT.

CHANGES

Apple reserves the right at any time to modify this Agreement and to impose new or additional terms or conditions on your use of the App and Book Services. Such modifications and additional terms and conditions will be effective immediately and incorporated into this Agreement. Your continued use of the App and Book Services will be deemed acceptance thereof.

MISCELLANEOUS

This Agreement constitutes the entire agreement between you and Apple and governs your use of the App and Book Services, superseding any prior agreements between you and Apple. You also may be subject to additional terms and conditions that may apply when you use affiliate services, certain App and Book Products, third-party content, or third-party software. If any part of this Agreement is held invalid or unenforceable, that portion shall be construed in a manner consistent with applicable law to reflect, as nearly as possible, the original intentions of the parties, and the remaining portions shall remain in full force and effect. Apple's failure to enforce any right or provisions in this Agreement will not constitute a waiver of such or any other provision. Apple will not be responsible for failures to fulfill any obligations due to causes beyond its control.

The App and Book Services are operated by Apple from its offices in the United States. You agree to comply with all local, state, federal, and national laws, statutes, ordinances, and regulations that apply to your use of the App and Book Services. All transactions on the App and Book Services are governed by California law, without giving effect to its conflict of law provisions. Your use of the App and Book Services may also be subject to other laws. You expressly agree that exclusive jurisdiction for any claim or dispute with Apple or relating in any way to your use of the App And Book Services resides in the courts in the State of California. Risk of loss and title for all electronically delivered transactions pass to the purchaser in California upon electronic transmission to the recipient. No Apple employee or agent has the authority to vary this Agreement.

Apple may notify you with respect to the App and Book Services by sending an email message to your Account email address or a letter via postal mail to your Account mailing address, or by posting on the App and Book Services. Notices shall become effective immediately.

Apple reserves the right to take steps Apple believes are reasonably necessary or appropriate to enforce and/or verify compliance with any part of this Agreement. You agree that Apple has the right, without liability to you, to disclose any Registration Data and/or Account information to law enforcement authorities, government officials, and/or a third party, as Apple believes is reasonably necessary or appropriate to enforce and/or verify compliance with any part of this Agreement (including but not limited to Apple's right to cooperate with any legal process relating to your use of the App and Book Services and/or App and Book Products, and/or a third-party claim that your use of the App and Book Services and/or App and Book Products is unlawful and/or infringes such third party's rights).

STATUTORY EXCEPTIONS FOR PUBLIC INSTITUTIONS

If you are a qualified public educational or government institution and any part of this Agreement, such as, by way of example, all or part of the indemnification section, is invalid or unenforceable against you because of applicable state or federal law, then that portion shall be deemed invalid or unenforceable, as the case may be, and instead construed in a manner most consistent with applicable governing law. If California law is precluded, this Agreement shall be construed under the laws of the state in which your public educational or government institution is located.

ADDITIONAL MAC APP STORE AND APP STORE TERMS AND CONDITIONS

LICENSE OF MAC APP STORE AND APP STORE PRODUCTS

The software products made available through the Mac App Store and App Store (collectively, the “App Store Products”) are licensed, not sold, to you. There are two (2) categories of App Store Products, as follows: (i) those App Store Products that have been developed, and are licensed to you, by Apple (“Apple Products”); and (ii) those App Store Products that have been developed, and are licensed to you, by a third-party developer (

“Third-Party Products”). The category of a particular App Store Product (Apple Product or Third-Party Product) is identified on the Mac App Store application or App Store application.

Your license to each App Store Product is subject to the Licensed Application End User License Agreement set forth below, and you agree that such terms will apply unless the App Store Product is covered by a valid end user license agreement entered into between you and the licensor of that App Store Product (the “Application Provider”), in which case the Application Provider’s end user license agreement will apply to that App Store Product. The Application Provider reserves all rights in and to the App Store Product not expressly granted to you.

You acknowledge that the license to each Apple Product that you obtain through the App Store Services, as defined below, is a binding agreement between you and Apple. You acknowledge that: you are acquiring the license to each Third-Party Product from the Application Provider; Apple is acting as agent for the Application Provider in providing each such Third-Party Product to you; and Apple is not a party to the license between you and the Application Provider with respect to that Third-Party Product. The Application Provider of each Third-Party Product is solely responsible for that Third-Party Product, the content therein, any warranties to the extent that such warranties have not been disclaimed, and any claims that you or any other party may have relating to that Third-Party Product.

You acknowledge and agree that Apple and its subsidiaries are third-party beneficiaries of the Licensed Application End User License Agreement or the Application Provider’s end user license agreement, as the case may be, for each Third-Party Product. You also agree that, upon your acceptance of the terms and conditions of the license to any such Third-Party Product, Apple will have the right (and will be deemed to have accepted the right) to enforce such license against you as a third-party beneficiary thereof.

IN-APP PURCHASES

Certain App Store Products may include functionality that enables you to receive additional services, or licenses to additional functionality or content for use within the App Store Product ("In App Purchases"). In App Purchases that are consumed during the use of the App Store Product (for example, virtual ammunition) cannot be transferred among devices; can be downloaded only once; and after being downloaded, cannot be replaced. Once a consumable In App Purchase is acquired and received by you, Apple shall be without liability to you in the event of any loss, destruction, or damage. All In App Purchases are deemed App Store Products, and In App Purchases received within Third-Party Products are deemed Third-Party Products, and treated as such, for purposes of these terms and conditions.

You must authenticate to acquire In-App Purchases separately from any authentication to obtain App Store Products by entering your password when prompted, but once you have authenticated to obtain an In-App Purchase, you will be able to acquire additional In-App Purchases for fifteen minutes without re-entering your password. You can turn off the ability to acquire In-App Purchases on your iOS Device by following the steps outlined at http://support.apple.com/kb/HT4213.

IN-APP SUBSCRIPTIONS

Certain App Store Products may include functionality that enables you to acquire content on a subscription basis (“In App Subscriptions”). Paid In App Subscriptions are non-refundable. In App Subscriptions will automatically renew for the applicable time period you have selected, and, where applicable, your Account will be charged no more than 24-hours prior to the expiration of the current In App Subscription. You may cancel automatic renewal of paid In App Subscriptions by selecting Manage App Subscriptions in your Account and selecting the subscription you want to modify. The auto-renew feature of the subscription will be turned off if

the Application Provider increases the price of the subscription. You may cancel free In App Subscriptions by deleting the App Store Product from your device. Certain paid In App Subscriptions may offer a free trial period prior to charging your Account. If you decide you do not want to purchase the In App Subscription, turn off auto-renewal in your Account settings during the free trial period. Certain In App Subscriptions may be designated as “Newsstand” products, in which case they will appear only within the Newsstand application on your device after download. You should also review additional information about the paid In App Subscription offer at the point of sale within the App Store Product. We may ask for your permission to provide the name, email address and zip code listed in your Account to the Application Provider so that the Application Provider can send you marketing messages about its own products in accordance with its publicly posted privacy policy. Once the Application Provider has this information, it will be treated in accordance with the Application Provider’s privacy policy. We encourage you to learn about the privacy practices of the Application Provider before agreeing to give it your personal information. For more information, please review the Application Provider’s privacy policy or contact the Application Provider directly.

GENIUS FOR APPS

When you opt in to the Genius for Apps feature, Apple will, from time to time, automatically collect information related to certain of your App Store Products, such as your time spent with each App Store Product and the number of times each App Store Product is launched. This information will be stored anonymously and will not be associated with your name or Account. When you use the Genius for Apps feature, Apple will use this information, as well as other information, such as your App Store Product download history, to give personalized recommendations to you.

Apple may use this information and combine it with aggregated information from other users who opt in to this feature, your iTunes Store purchase history data, your App Store download data, aggregated App Store Product download data from other users, and other information like customer ratings of App Store Products, to:

• Provide you with recommendations regarding App Store Products, media, and other products and services that you may wish to purchase, download, or use.

• Provide recommendations to other users.

At all times your information will be treated in accordance with Apple’s Privacy Policy.

Once you opt in to the Genius for Apps feature on a Genius-capable system, you will be able to create Genius recommendations on that system.

If you prefer that we do not collect and use information from your device or system in this manner, you should not enable the Genius feature. You can opt out at any time by turning off the Genius feature from the App Store Account page on your device and/or system. If you have elected to share Genius for Apps information from multiple devices and/or systems, you need to turn off the Genius feature from each device and system to opt out.

MAC APP STORE PRODUCT USAGE RULES

Except as otherwise set forth herein,

(i) If you are an individual acting in your personal capacity, you may download and use an application from the Mac App Store (“Mac App Store Product”) for personal, non-commercial use on any Apple-branded products running Mac OS X (“Mac Computer”) that you own or control.

(ii) If you are a commercial enterprise or educational institution, you may download a Mac App Store Product for use by either (a) a single individual on each of the Mac Computer(s) used by that individual that you own or control or (b) multiple individuals on a single shared Mac Computer that you own or control. For example, a single employee may use a Mac App Store Product on both the employee’s desktop Mac Computer and laptop Mac Computer, or multiple students may serially use a Mac App Store Product on a single Mac Computer located at a resource center or library. For the sake of clarity, each Mac Computer used serially by multiple users requires a separate license.

(iii) Use may require sign-in with the Apple ID used to download the Mac App Store Product from the Mac App Store. Mac App Store Products can be updated through the Mac App Store only.

APP STORE PRODUCT USAGE RULES

(i) If you are an individual acting in your personal capacity, you may download and sync an App Store Product for personal, noncommercial use on any iOS Device you own or control.

(ii) If you are a commercial enterprise or educational institution, you may download and sync an App Store Product for use by either (a) a single individual on one or more iOS Devices used by that individual that you own or control or (b) multiple individuals, on a single shared iOS Device you own or control. For example, a single employee may use an App Store Product on both the employee's iPhone and iPad, or multiple students may serially use an App Store Product on a single iPad located at a resource center or library. For the sake of clarity, each iOS Device used serially by multiple users requires a separate license.

(iii) You shall be able to store App Store Products from up to five different Accounts at a time on a compatible iOS Device.

(iv) You shall be able to manually sync App Store Products from at least one iTunes-authorized device to iOS Devices that have manual sync mode, provided that the App Store Product is associated with an Account on the primary iTunes-authorized device, where the primary iTunes-authorized device is the one that was first synced with the iOS Device or the one that you subsequently designate as primary using the iTunes application.

IMPORTANT SAFETY INFORMATION

To avoid muscle, joint, or eye strain during video game play, you should always take frequent breaks from playing, and take a longer rest if you experience any soreness, fatigue, or discomfort. A very small percentage of people may experience seizures or blackouts when exposed to flashing lights or patterns, including while playing video games or watching videos. Symptoms may include dizziness, nausea, involuntary movements, loss of awareness, altered vision, tingling, numbness, or other discomforts. Consult a doctor before playing video games if you have ever suffered these or similar symptoms, and stop playing immediately and see a doctor if they occur during game play. Parents should monitor their children’s video game play for signs of symptoms.

IPOD GAMES

Updates to your existing compatible iPod device’s firmware may render your purchased version of an iPod Game incompatible. Games purchased from the iTunes Store may not be compatible with future generations of the iPod.

MAC APP STORE AND APP STORE PRODUCT MAINTENANCE AND SUPPORT

Apple will be responsible for providing any maintenance and support services with respect to the Apple Products only, as specified in the Licensed Application End User License Agreement or the separate end user license agreement, as the case may be, or as required under applicable law. The Application Provider of any Third-Party Product will be solely responsible for providing maintenance and support services with respect to that Product, as specified in the Licensed Application End User License Agreement or the Application Provider end user license agreement, as the case may be, or as required under applicable law.

LICENSED APPLICATION END USER LICENSE AGREEMENT

The Mac App Store Products and App Store Products (collectively, "App Store Product(s)") made available through the Mac App Store Service and App Store Service (collectively, "App Store Service(s)") are licensed, not sold, to you. Your license to each App Store Product that you obtain through the App Store Services is subject to your prior acceptance of this Licensed Application End User License Agreement ("Standard EULA"), and you agree that the terms of this Standard EULA will apply to each App Store Product that you license through the App Store Service, unless that App Store Product is covered by a valid end user license agreement between you and the Application Provider of that App Store Product, in which case the terms of that separate end user license agreement will govern. Your license to any Apple Product under this Standard EULA or separate end user license agreement is granted by Apple, and your license to any Third-Party Product under this Standard EULA or separate end user license agreement is granted by the Application Provider of that Third-Party Product. Any App Store Product that is subject to the license granted under this Standard EULA is referred to herein as the "Licensed Application". The Application Provider or Apple as applicable ("Licensor") reserves all rights in and to the Licensed Application not expressly granted to you under this Standard EULA.

a. Scope of License: This license granted to you for the Licensed Application by Licensor is limited to a nontransferable license to use the Licensed Application on any Apple-branded products running iOS (including but not limited to iPad, iPhone, and iPod touch) ("iOS Devices") or Mac OS X ("Mac Computers"), as applicable (collectively, "Apple Device(s)") that you own or control and as permitted by the usage rules set forth in the Mac App Store, App Store and iBookstore Terms and Conditions (the "Usage Rules"). This license does not allow you to use the Licensed Application on any Apple Device that you do not own or control, and except as provided in the Usage Rules, you may not distribute or make the Licensed Application available over a network where it could be used by multiple devices at the same time. You may not rent, lease, lend, sell, transfer redistribute, or sublicense the Licensed Application and, if you sell your Mac Computer or iOS Device to a third party, you must remove the Licensed Application from the Mac Computer or iOS Device before doing so. You may not copy (except as expressly permitted by this license and the Usage Rules), decompile, reverse-engineer, disassemble, attempt to derive the source code of, modify, or create derivative works of the Licensed Application, any updates, or any part thereof (except as and only to the extent that any foregoing restriction is prohibited by applicable law or to the extent as may be permitted by the licensing terms governing use of any open-sourced components included with the Licensed Application). Any attempt to do so is a violation of the rights of the Licensor and its licensors. If you breach this restriction, you may be subject to prosecution and damages.

The terms of this license will govern any upgrades provided by Licensor that replace and/or supplement the original Licensed Application, unless such upgrade is accompanied by a separate license in which case the terms of that license will govern.

b. Consent to Use of Data: You agree that Licensor may collect and use technical data and related information—including but not limited to technical information about your device, system and application software, and peripherals—that is gathered periodically to facilitate the provision of software updates, product support, and other services to you (if any) related to the Licensed Application. Licensor may use this

information, as long as it is in a form that does not personally identify you, to improve its products or to provide services or technologies to you.

c. Termination. The license is effective until terminated by you or Licensor. Your rights under this license will terminate automatically without notice from the Licensor if you fail to comply with any term(s) of this license. Upon termination of the license, you shall cease all use of the Licensed Application and destroy all copies, full or partial, of the Licensed Application.

d. External Services; Third-Party Materials. The Licensed Application may enable access to Licensor’s and/or third-party services and websites (collectively and individually, "External Services"). Use of the External Services requires Internet access and use of certain External Services requires you to accept additional terms. By using this software in connection with an iTunes Store account, you agree to the latest iTunes Store Terms and Conditions and Usage Rules, which you may access and review at http://www.apple.com/legal/internet-services/itunes/ww/.

You understand that by using any of the External Services, you may encounter content that may be deemed offensive, indecent, or objectionable, which content may or may not be identified as having explicit language, and that the results of any search or entering of a particular URL may automatically and unintentionally generate links or references to objectionable material. Nevertheless, you agree to use the External Services at your sole risk and that neither the Licensor nor its agents shall have any liability to you for content that may be found to be offensive, indecent, or objectionable.

Certain External Services may display, include or make available content, data, information, applications or materials from third parties (“Third Party Materials”) or provide links to certain third party web sites. By using the External Services, you acknowledge and agree that neither the Licensor nor its agents is responsible for examining or evaluating the content, accuracy, completeness, timeliness, validity, copyright compliance, legality, decency, quality or any other aspect of such Third Party Materials or web sites. Neither the Licensor nor its agents warrant or endorse and does not assume and will not have any liability or responsibility to you or any other person for any third-party services, Third Party Materials or web sites, or for any other materials, products, or services of third parties. Third Party Materials and links to other web sites are provided solely as a convenience to you.

Financial information displayed by any External Services is for general informational purposes only and should not be relied upon as investment advice. Before executing any securities transaction based upon information obtained through the External Services, you should consult with a financial or securities professional who is legally qualified to give financial or securities advice in your country or region. Location data provided by any External Services is for basic navigational purposes only and is not intended to be relied upon in situations where precise location information is needed or where erroneous, inaccurate, time-delayed or incomplete location data may lead to death, personal injury, property or environmental damage. Neither the Licensor, nor its agents, nor any of its content providers guarantees the availability, accuracy, completeness, reliability, or timeliness of stock information, location data or any other data displayed by any External Services.

You agree that the External Services contain proprietary content, information and material that is owned by Licensor and/or its agents or licensors, and is protected by applicable intellectual property and other laws, including but not limited to copyright, and that you will not use such proprietary content, information or materials in any way whatsoever except for permitted use of the External Services or in any manner that is inconsistent with the terms of this Standard EULA or that infringes any intellectual property rights of a third party or Apple. No portion of the External Services may be reproduced in any form or by any means. You agree not to modify, rent, lease, loan, sell, distribute, or create derivative works based on the External Services, in any manner, and you shall not exploit the External Services in any unauthorized way whatsoever, including but not

limited to, using the External Services to transmit any computer viruses, worms, trojan horses or other malware, or by trespass or burdening network capacity. You further agree not to use the External Services in any manner to harass, abuse, stalk, threaten, defame or otherwise infringe or violate the rights of any other party, and that neither Licensor nor its agents is in any way responsible for any such use by you, nor for any harassing, threatening, defamatory, offensive, infringing or illegal messages or transmissions that you may receive as a result of using any of the External Services.

In addition, External Services and Third Party Materials that may be accessed from, displayed on or linked to from the Apple Devices are not available in all languages or in all countries or regions. Licensor makes no representation that such External Services and Materials are appropriate or available for use in any particular location. To the extent you choose to use or access such External Services and Materials, you do so at your own initiative and are responsible for compliance with any applicable laws, including but not limited to applicable local laws. Licensor reserves the right to change, suspend, remove, or disable access to any External Services at any time without notice. In no event will Licensor be liable for the removal of or disabling of access to any such External Services. Licensor may also impose limits on the use of or access to certain External Services, in any case and without notice or liability.

e. NO WARRANTY: YOU EXPRESSLY ACKNOWLEDGE AND AGREE THAT USE OF THE LICENSED APPLICATION IS AT YOUR SOLE RISK AND THAT THE ENTIRE RISK AS TO SATISFACTORY QUALITY, PERFORMANCE, ACCURACY, AND EFFORT IS WITH YOU. TO THE MAXIMUM EXTENT PERMITTED BY APPLICABLE LAW, THE LICENSED APPLICATION AND ANY SERVICES PERFORMED OR PROVIDED BY THE LICENSED APPLICATION ARE PROVIDED "AS IS" AND “AS AVAILABLE”, WITH ALL FAULTS AND WITHOUT WARRANTY OF ANY KIND, AND LICENSOR HEREBY DISCLAIMS ALL WARRANTIES AND CONDITIONS WITH RESPECT TO THE LICENSED APPLICATION AND ANY SERVICES, EITHER EXPRESS, IMPLIED, OR STATUTORY, INCLUDING, BUT NOT LIMITED TO, THE IMPLIED WARRANTIES AND/OR CONDITIONS OF MERCHANTABILITY, OF SATISFACTORY QUALITY, OF FITNESS FOR A PARTICULAR PURPOSE, OF ACCURACY, OF QUIET ENJOYMENT, AND OF NONINFRINGEMENT OF THIRD-PARTY RIGHTS. LICENSOR DOES NOT WARRANT AGAINST INTERFERENCE WITH YOUR ENJOYMENT OF THE LICENSED APPLICATION, THAT THE FUNCTIONS CONTAINED IN OR SERVICES PERFORMED OR PROVIDED BY THE LICENSED APPLICATION WILL MEET YOUR REQUIREMENTS, THAT THE OPERATION OF THE LICENSED APPLICATION OR SERVICES WILL BE UNINTERRUPTED OR ERROR-FREE, OR THAT DEFECTS IN THE LICENSED APPLICATION OR SERVICES WILL BE CORRECTED. NO ORAL OR WRITTEN INFORMATION OR ADVICE GIVEN BY LICENSOR OR ITS AUTHORIZED REPRESENTATIVE SHALL CREATE A WARRANTY. SHOULD THE LICENSED APPLICATION OR SERVICES PROVE DEFECTIVE, YOU ASSUME THE ENTIRE COST OF ALL NECESSARY SERVICING, REPAIR, OR CORRECTION. SOME JURISDICTIONS DO NOT ALLOW THE EXCLUSION OF IMPLIED WARRANTIES OR LIMITATIONS ON APPLICABLE STATUTORY RIGHTS OF A CONSUMER, SO THE ABOVE EXCLUSION AND LIMITATIONS MAY NOT APPLY TO YOU.

f. Limitation of Liability. TO THE EXTENT NOT PROHIBITED BY LAW, IN NO EVENT SHALL LICENSOR BE LIABLE FOR PERSONAL INJURY OR ANY INCIDENTAL, SPECIAL, INDIRECT, OR CONSEQUENTIAL DAMAGES WHATSOEVER, INCLUDING, WITHOUT LIMITATION, DAMAGES FOR LOSS OF PROFITS, LOSS OF DATA, BUSINESS INTERRUPTION, OR ANY OTHER COMMERCIAL DAMAGES OR LOSSES, ARISING OUT OF OR RELATED TO YOUR USE OR INABILITY TO USE THE LICENSED APPLICATION, HOWEVER CAUSED, REGARDLESS OF THE THEORY OF LIABILITY (CONTRACT, TORT, OR OTHERWISE) AND EVEN IF LICENSOR HAS BEEN ADVISED OF THE POSSIBILITY OF SUCH DAMAGES. SOME JURISDICTIONS DO NOT ALLOW THE LIMITATION OF LIABILITY FOR PERSONAL INJURY, OR OF INCIDENTAL OR CONSEQUENTIAL

DAMAGES, SO THIS LIMITATION MAY NOT APPLY TO YOU. In no event shall Licensor’s total liability to you for all damages (other than as may be required by applicable law in cases involving personal injury) exceed the amount of fifty dollars ($50.00). The foregoing limitations will apply even if the above stated remedy fails of its essential purpose.

g. You may not use or otherwise export or re-export the Licensed Application except as authorized by United States law and the laws of the jurisdiction in which the Licensed Application was obtained. In particular, but without limitation, the Licensed Application may not be exported or re-exported (a) into any U.S.-embargoed countries or (b) to anyone on the U.S. Treasury Department's Specially Designated Nationals List or the U.S. Department of Commerce Denied Persons List or Entity List. By using the Licensed Application, you represent and warrant that you are not located in any such country or on any such list. You also agree that you will not use these products for any purposes prohibited by United States law, including, without limitation, the development, design, manufacture, or production of nuclear, missile, or chemical or biological weapons.

h. The Licensed Application and related documentation are "Commercial Items", as that term is defined at 48 C.F.R. §2.101, consisting of "Commercial Computer Software" and "Commercial Computer Software Documentation", as such terms are used in 48 C.F.R. §12.212 or 48 C.F.R. §227.7202, as applicable. Consistent with 48 C.F.R. §12.212 or 48 C.F.R. §227.7202-1 through 227.7202-4, as applicable, the Commercial Computer Software and Commercial Computer Software Documentation are being licensed to U.S. Government end users (a) only as Commercial Items and (b) with only those rights as are granted to all other end users pursuant to the terms and conditions herein. Unpublished-rights reserved under the copyright laws of the United States.

i. The laws of the State of California, excluding its conflicts of law rules, govern this license and your use of the Licensed Application. Your use of the Licensed Application may also be subject to other local, state, national, or international laws.

ADDITIONAL IBOOKSTORE TERMS AND CONDITIONS

PURCHASE OF IBOOKSTORE PRODUCTS

You acknowledge that you are purchasing the content made available through the iBookstore Service (the “iBookstore Products”) from the third-party provider of that iBookstore Product (the “Publisher”); Apple is acting as agent for the Publisher in providing each such iBookstore Product to you; Apple is not a party to the transaction between you and the Publisher with respect to that iBookstore Product; and the Publisher of each iBookstore Product reserves the right to enforce the terms of use relating to that iBookstore Product. The Publisher of each iBookstore Product is solely responsible for that iBookstore Product, the content therein, any warranties to the extent that such warranties have not been disclaimed, and any claims that you or any other party may have relating to that iBookstore Product or your use of that iBookstore Product.

IBOOKSTORE PRODUCT USAGE RULES

(i) You shall be authorized to use the iBookstore Products only for personal, noncommercial use.

(ii) You shall be able to store iBookstore Products from up to five different Accounts at a time on certain iOS-based devices, such as an iPad, iPod touch, or iPhone.

(iii) You shall be able to store iBookstore Products on five iTunes-authorized devices at any time.

(iv) The delivery of iBookstore Products does not transfer to you any promotional use rights in the iBookstore Products or any rights to burn the iBookstore Products to disc.

(v) You shall be able to manually sync iBookstore Products from at least one iTunes-authorized device to devices that have manual sync mode, provided that the iBookstore Product is associated with an Account on the primary iTunes-authorized device, where the primary iTunes-authorized device is the one that was first synced with the device or the one that you subsequently designate as primary using iTunes.

Last Updated: December 3, 2012

Appendix 19: Sample eCardio Report

eCardio

Cardiac Report
Day 8 - Stable

#3 Received 05/31/2013 @ 12:22 PM (CT)

eCardio Diagnostics, LLC
888.500.3522
www.ecardio.com

Patient: AliveCor ScriptsTest01

Patient ID	Gender	Date of Birth	Phone	Monitor
01122400226	Male	01/01/1970 (43 years)	(281)760-0500	AliveCor Heart Monitor

Physician	Practice
LARRY DOCTOR-	TOLLWAY CARDIOLOGY

Diagnosis (Indication for Monitoring)

Enrollment Info

05/24/2013 - 02/16/2016

Day 8 of 999

Baseline Reference

Recorded: 05/24/2013 01:11 PM (CT) Baseline

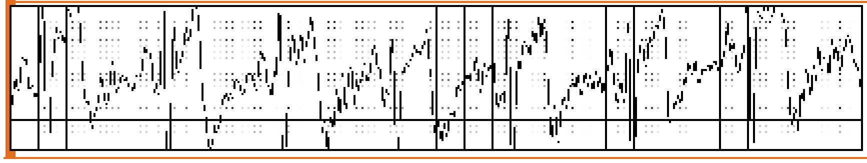

Symptom: TEST
Activity: TEST
Trans: 05/24/2013 01:11 PM
Type: Auto-Detected
HR: 60.0-60.0 BPM

Current Event

Stable

Measurements:

Rate (bpm)	69.5
PR (s)	0.15
QRS (s)	0.07
QT (s)	0.36
QTc (s)	0.39

Measurements:

Rate (bpm)	69.5
PR (s)	0.15
QRS (s)	0.06
QT (s)	0.36
QTc (s)	0.39

Current Transmission #3

Received 05/31/2013 @ 12:22 PM (CT)

Patient Symptom: Auto Detect **Patient Activity:** Wireless Event **Location:** Wireless Event

Report Analysis: Sinus Rhythm w/ Artifact

Comments: Tech:Esther Rivera, CCT

Interpreting Physician: ______________________

I have personally reviewed and interpreted this report.

Report copyright 2004 - 2013 eCardio Diagnostics, LLC. Copying permitted by the intended recipient. Any other copying and adapting prohibited under penalty of federal and international law. Page 1 of 2

Patient: **AliveCor ScriptsTest01** **Stable** Physician: LARRY DOCTOR-WOODLANDS

D.O.B.: 01/01/1970 Age: 43 Gender: Male Dx/Sx:

Full Disclosure

Recorded: 5/31/2013 @ 12:21 PM (CT) 25 mm/sec, 8 mm/mV Continues->

Recorded: 5/31/2013 @ 12:21 PM (CT) 25 mm/sec, 8 mm/mV Continues->

Recorded: 5/31/2013 @ 12:22 PM (CT) 25 mm/sec, 8 mm/mV Continues->

Recorded: 5/31/2013 @ 12:22 PM (CT) 25 mm/sec, 8 mm/mV Continues->

Recorded: 5/31/2013 @ 12:22 PM (CT) 25 mm/sec, 8 mm/mV End

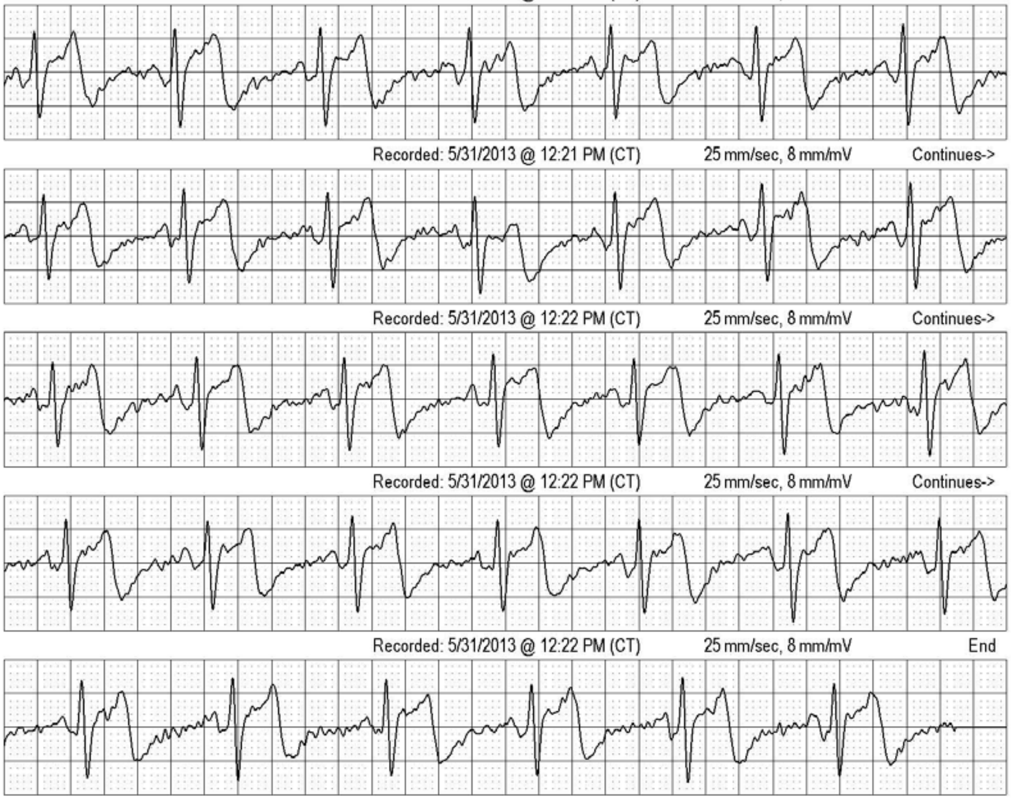

Report copyright 2004 - 2013 eCardio Diagnostics, LLC. Copying permitted by the intended recipient. Any other copying and adapting prohibited under penalty of federal and international law.

Appendix 20: E-mail to Scripps Employees

Dear Colleague,

The Scripps Translational Science (STSI) Institute in collaboration with Scripps Wellness has launched the **"Wired for Health"** study to evaluate whether patient use of iPhone 4s together with mobile health devices can reduce health care costs.

STSI is recruiting 200 Scripps employees and family members with one or more of the following conditions: diabetes, high blood pressure and heart arrhythmias.

The people enrolled in the study will be those who have seen their doctor often or have been to the hospital over the past year. The potential study participants will be identified by HealthComp, the administrator for Scripps Health employee medical claims. Their information will be kept strictly confidential.

Half of the participants will make up the control group, while the other half will receive a wireless device to monitor their condition together with an iPhone 4. Both the control and monitoring group participants will be enrolled in HealthComp's Disease Management program, which includes one-on-one contact with a Health Comp nurse and condition-specific health education.

The purpose of the study is to see whether patients who actively track their health conditions through mobile devices linked with a web-based health portal will have more success managing their health conditions and, as a result, spend fewer health care dollars.

If you or a family member (covered under your Scripps insurance plan) have been diagnosed with one or more of the following: **diabetes, high blood pressure or cardiac arrhythmia**, you may be eligible to participate in the "Wired for Health" study. To learn more, please contact Health Comp at 1-800-442-7247 ext 2544 or email wellness@healthcomp.com.

For more information go to: http://www.scripps.org/news_items/4557-scripps-launches-study-to-assess-role-of-mobile-health-devices-in-lowering-health-costs

Sincerely,

Vic Buzachero
Corporate Senior Vice President
Human Resources, Innovation and Performance Management

Appendix 21

SCRIPPS WIRED FOR HEALTH STUDY – 6 MONTH SURVEY

III. Health History

1 How is your health? (pull down: very good, good, average, poor, very poor)

2 In the past 6 months, how many times per week do you/did you use tobacco (if you are a current or previous user of tobacco)?

3 In the past 6 months, how many alcoholic drinks per week do you/did you have (if you are a current or previous drinker)?

4 List any and all additional health conditions that you have been told by a doctor or other health professional that you currently have.

5 Please list all of the medications for which you currently have a prescription from a doctor or other health professional.

6 Have you experienced any changes in your health during the past 6 months?

II. Health Literacy (single item)

Over the past 6 months, how often do you need to have someone help you when you read instructions, pamphlets, or other written material from your doctor or pharmacy? (pull down: Never, Rarely, Sometimes, Often, and Always)

III. Perceived Health Services Utilization (PERC)

1 Please answer the following:

a. In the past 6 months, how many times did you visit a physician? Do **not** include visits while in the hospital or to a hospital emergency room. Fill in with “0” or another number. ____times

b. In the past 6 months, how many times did you go to a **hospital** emergency room? Fill in with “0” or another number. ____times

c. How many different **times** did you stay in a hospital **overnight** or longer in the past 6 months? Fill in with “0” or another number. ____times

d. How many total **nights** did you spend in the hospital in the past 6 months? Fill in with “0” or another number. ____nights

IV. Health Locus of Control (MHL Form C)

Each item below is a belief statement about your medical condition with which you may agree or disagree. Beside each statement is a scale which ranges from strongly disagree (1) to strongly agree (6). For each item we would like you to circle the number that represents the extent to which you agree or disagree with that statement. The more you agree with a statement, the higher will be the number you circle. The more you disagree with a statement, the lower will be the number you circle. Please make sure that you answer **EVERY ITEM** and that you circle **ONLY ONE** number per item. This is a measure of your personal beliefs; obviously, there are no right or wrong answers.

		**Strongly Disagree**	**Moderately Disagree**	**Slightly Disagree**	**Slightly Agree**	**Moderately Agree**	**Strongly Agree**
1	If my condition worsens, it is my own behavior which determines how soon I will feel better again.	1	2	3	4	5	6
2	As to my condition, what will be will be.	1	2	3	4	5	6
3	If I see my doctor regularly, I am less likely to have problems with my condition.	1	2	3	4	5	6
4	Most things that affect my condition happen to me by chance.	1	2	3	4	5	6
5	Whenever my condition worsens, I should consult a medically trained professional.	1	2	3	4	5	6
6	I am directly responsible for my condition getting better or worse.	1	2	3	4	5	6
7	Other people play a big role in whether my condition improves, stays the same, or gets worse.	1	2	3	4	5	6
8	Whatever goes wrong with my condition is my own fault.	1	2	3	4	5	6
9	Luck plays a big part in determining how my condition improves.	1	2	3	4	5	6
10	In order for my condition to improve, it is up to other people to see that the right things happen.	1	2	3	4	5	6
11	Whatever improvement occurs with my condition is largely a matter of good fortune.	1	2	3	4	5	6
12	The main thing which affects my condition is what I myself do.	1	2	3	4	5	6
13	I deserve the credit when my condition improves and the blame when it gets worse.	1	2	3	4	5	6
14	Following doctor's orders to the letter is the best way to keep my condition from getting any worse.	1	2	3	4	5	6
15	If my condition worsens, it's a matter of fate.	1	2	3	4	5	6
16	If I am lucky, my condition will get better.	1	2	3	4	5	6

17	If my condition takes a turn for the worse, it is because I have not been taking proper care of myself.	1	2	3	4	5	6
18	The type of help I receive from other people determines how soon my condition improves.	1	2	3	4	5	6

V. Health Self-Efficacy (SE for Managing Chronic Disease 6-Item Scale)

We would like to know how confident you are in doing certain activities. For each of the following questions, please choose the number that corresponds to your confidence that you can do the tasks regularly at the present time.

1. How confident are you that you can keep the fatigue caused by your disease from interfering with the things you want to do?

   not at all | | | | | | | | | | totally
   confident 1 2 3 4 5 6 7 8 9 10 confident

2. How confident are you that you can keep the physical discomfort or pain of your disease from interfering with the things you want to do?

   not at all | | | | | | | | | | totally
   confident 1 2 3 4 5 6 7 8 9 10 confident

3. How confident are you that you can keep the emotional distress caused by your disease from interfering with the things you want to do?

   not at all | | | | | | | | | | totally
   confident 1 2 3 4 5 6 7 8 9 10 confident

4. How confident are you that you can keep any other symptoms or health problems you have from interfering with the things you want to do?

   not at all | | | | | | | | | | totally
   confident 1 2 3 4 5 6 7 8 9 10 confident

5. How confident are you that you can do the different tasks and activities needed to manage your health condition so as to reduce you need to see a doctor?

   not at all | | | | | | | | | | totally
   confident 1 2 3 4 5 6 7 8 9 10 confident

6. How confident are you that you can do things other than just taking medication to reduce how much you illness affects your everyday life?

   not at all | | | | | | | | | | totally
   confident 1 2 3 4 5 6 7 8 9 10 confident

VI. Patient Activation (Me and My Health)

INSTRUCTIONS: For each statement below please mark an X in the box to the right that best describes how much you disagree—agree with the statement as it applies to you personally.

There are no right or wrong answers. Your answers should be what is true to you and not just what you think the doctor wants you to say.

		Strongly Disagree	Disagree	Agree	Strongly Agree
1	When all is said and done, I am the person who is responsible for managing my health condition.				
2	Taking an active role in my own health care is the most important factor in determining my health and ability to function.				
3	I am confident that I can take actions that will help prevent or minimize some symptoms or problems associated with my health condition.				
4	I know what each of my prescribed medications do.				
5	I am confident that I can tell when I need to go get medical care and when I can handle a health problem myself.				
6	I am confident I can tell a doctor concerns I have even when he or she does not ask.				
7	I am confident that I can follow through on medical treatments I need to do at home.				
8	I understand the nature and causes of my health condition(s).				
9	I know the different medical treatment options available for my health condition.				
10	I have been able to maintain the lifestyle changes for my health condition that I have made.				
11	I know how to prevent further problems with my health condition.				
12	I am confident I can figure out solutions when new situations or problems arise with my health conditions.				
13	I am confident that I can maintain lifestyle changes, like diet and exercise, even during times of stress.				

VII. Health-Related Quality of Life (SF12)

For each of the following questions, please circle the number that best describes your answer.

1. In general, would you say your health is:
_____ Excellent (1)
_____ Very Good (2)
_____ Good (3)
_____ Fair (4)
_____ Poor (5)

The following two questions are about activities you might do during a typical day. Does YOUR HEALTH NOW LIMIT YOU in these activities? If so, how much?

2. MODERATE ACTIVITIES, such as moving a table, pushing a vacuum cleaner, bowling, or playing golf:
_____ Yes, Limited A Lot (1)
_____ Yes, Limited A Little (2)
_____ No, Not Limited At All (3)

3. Climbing SEVERAL flights of stairs:
_____ Yes, Limited A Lot (1)
_____ Yes, Limited A Little (2)
_____ No, Not Limited At All (3)

During the PAST 4 WEEKS have you had any of the following problems with your work or other regular activities AS A RESULT OF YOUR PHYSICAL HEALTH?

4. ACCOMPLISHED LESS than you would like:
_____ Yes (1)
_____ No (2)

5. Were limited in the KIND of work or other activities:
_____ Yes (1)
_____ No (2)

During the PAST 4 WEEKS, were you limited in the kind of work you do or other regular activities AS A RESULT OF ANY EMOTIONAL PROBLEMS (such as feeling depressed or anxious)?

6. ACCOMPLISHED LESS than you would like:
_____ Yes (1)
_____ No (2)

7. Didn't do work or other activities as CAREFULLY as usual:
_____ Yes (1)
_____ No (2)

8. During the PAST 4 WEEKS, how much did PAIN interfere with your normal work (including both work outside the home and housework)?
_____ Not At All (1)
_____ A Little Bit (2)
_____ Moderately (3)
_____ Quite A Bit (4)
_____ Extremely (5)

The next three questions are about how you feel and how things have been DURING THE PAST 4 WEEKS. For each question, please give the one answer that comes closest to the way you have been feeling. How much of the time during the PAST 4 WEEKS –

9. Have you felt calm and peaceful?
_____ All of the Time (1)
_____ Most of the Time (2)
_____ A Good Bit of the Time (3)
_____ Some of the Time (4)
_____ A Little of the Time (5)
_____ None of the Time (6)

10. Did you have a lot of energy?
_____ All of the Time (1)
_____ Most of the Time (2)
_____ A Good Bit of the Time (3)
_____ Some of the Time (4)
_____ A Little of the Time (5)
_____ None of the Time (6)

11. Have you felt downhearted and blue?
_____ All of the Time (1)
_____ Most of the Time (2)
_____ A Good Bit of the Time (3)
_____ Some of the Time (4)
_____ A Little of the Time (5)
_____ None of the Time (6)

12. During the PAST 4 WEEKS, how much of the time has your PHYSICAL HEALTH OR EMOTIONAL PROBLEMS interfered with your social activities (like visiting with friends, relatives, etc.)?
_____ All of the Time (1)
_____ Most of the Time (2)
_____ A Good Bit of the Time (3)
_____ Some of the Time (4)
_____ A Little of the Time (5)
_____ None of the Time (6)

VIII. Physician Communication

The purpose of this questionnaire is to obtain your views about your communications with your **physician (the one you see most often) about your medical problems over the past 6 months.** Please show how strongly you agree or disagree with these statements by **checking the box that best fits your views.**

		Strongly Disagree	Disagree	Slightly Disagree	Not Sure	Slightly Agree	Agree	Strongly Agree
**I DO A GOOD JOB OF:**								
1.	Explaining my medical problem.							
2.	Describing the symptoms of my medical problem(s).							
3.	Answering the doctor's questions thoroughly.							
4.	Answering the doctor's questions honestly.							
5.	Letting the doctor know when I didn't understand something.							
6.	Getting answers to my questions.							
7.	Getting all the information I need.							
**MY PHYSICIAN EXPLAINS THE FOLLOWING TO MY SATISFACTION:**								
1.	Explaining what my medical problems are.							
2.	Explaining the possible causes of my medical problems.							
3.	Explaining what I may be able to do to get better.							
4.	Reviewing or repeating important information.							
5.	Making sure I understand his/her explanations/directions.							
6.	Using language I can understand.							
7.	Encouraging me to ask questions.							
**Please indicate how strongly you agree or disagree with these statements:**								
	Overall, I am satisfied with my physician's communication regarding my medical problems.							

IX. Medication Adherence (Morisky 8-Item)

Question		Yes	No
1	Do you sometimes forget to take your medicine?		
2	People sometimes miss taking their medicines for reasons other than forgetting. Thinking over the past 2 weeks, were there any days when you did not take your medicine?		
3	Have you ever cut back or stopped taking your medicine without telling your doctor because you felt worse when you took it?		
4	When you travel or leave home, do you sometimes forget to bring along your medicine?		
5	Did you take all your medicines yesterday?		
6	When you feel like your symptoms are under control, do you sometimes stop taking your medicine?		
7	Taking medicine every day is a real inconvenience for some people. Do you ever feel hassled about sticking to your treatment plan?		

8. How often do you have difficulty remembering to take all your medicine?

__ A. Never/rarely

__ B. Once in a while

__ C. Sometimes

__ D. Usually

__ E. All the time

X. Exercise (GLTEQ)

1. During a typical 7-Day period (a week), how many times on the average do you do the following kinds of exercise for more than 15 minutes during your free time (write on each line the appropriate number).

	Times Per Week
a) STRENUOUS EXERCISE<br>(HEART BEATS RAPIDLY)<br>(e.g., running, jogging, hockey, football, soccer, squash, basketball, cross country skiing, judo, roller skating, vigorous swimming, vigorous long distance bicycling)	
b) MODERATE EXERCISE<br>(NOT EXHAUSTING)<br>(e.g., fast walking, baseball, tennis, easy bicycling, volleyball, badminton, easy swimming, alpine skiing, popular and folk dancing)	
c) MILD EXERCISE<br>(MINIMAL EFFORT)<br>(e.g., yoga, archery, fishing from river bank, bowling, horseshoes, golf, snow-mobiling, easy walking)	

2. During a typical 7-Day period (a week), in your leisure time, how often do you engage in any regular activity long enough to work up a sweat (heart beats rapidly)?

OFTEN	SOMETIMES	NEVER/RARELY
_____	_____	_____

XI. Use of Innovations

1. Please answer the following questions regarding your thoughts, views, and feelings about **new medical tests, treatments, and devices.**

		strongly disagree	disagree	neutral	agree	strongly agree
1.	If I heard of a new medical test, treatment, or device I would try to find out more about it					
2.	Among my friends, I am usually one of the first to find out about and/or utilize a new medical test, treatment, or device					
3.	In general, I am hesitant to undergo or utilize a new medical test, treatment, or device					

2. Please answer the following questions regarding your thoughts, views, and feelings about **the use of medical devices that can be used at home** to monitor various aspects of your health, including blood pressure, blood sugar, and heart rhythms.

		strongly disagree	disagree	neutral	agree	strongly agree
1.	The use of at-home medical devices for health monitoring is consistent with my approach to my health					
2.	The use of at-home medical devices for health monitoring fits well with my values and goals					
3.	The use of at-home medical devices for health monitoring is hard to understand					
4.	The use of at-home medical devices for health monitoring has many different parts					
5.	The use of at-home medical devices for health monitoring can help me stay healthier compared to other strategies, like seeing my doctor more often					

3. Please answer the following:

To me, new digital technologies like the Smart Phone are:

b. important	___:___:___:___:___:___:___	unimportant
c. boring	___:___:___:___:___:___:___	interesting
d. relevant	___:___:___:___:___:___:___	irrelevant
e. exciting	___:___:___:___:___:___:___	unexciting
f. means nothing	___:___:___:___:___:___:___	means a lot to me
g. appealing	___:___:___:___:___:___:___	unappealing
h. fascinating	___:___:___:___:___:___:___	mundane
i. worthless	___:___:___:___:___:___:___	valuable
j. involving	___:___:___:___:___:___:___	uninvolving
k. not needed	___:___:___:___:___:___:___	needed

XIII. Device Usability

Please answer the following in regards to your monitoring device or devices:

System Usability Scale (SUS)	Strongly disagree				Strongly agree
1. I think that I would like to use this system frequently	1	2	3	4	5
2. I found the system unnecessarily complex	1	2	3	4	5
3. I thought the system was easy to use	1	2	3	4	5
4. I think that I would need the support of a technical person to be able to use this system	1	2	3	4	5
5. I found the various functions in this system were well integrated	1	2	3	4	5
6. I thought there was too much inconsistency in this system	1	2	3	4	5
7. I would imagine that most people would learn to use this system very quickly	1	2	3	4	5
8. I found the system very cumbersome to use	1	2	3	4	5
9. I felt very confident using the system	1	2	3	4	5
10. I needed to learn a lot of things before I could get going with this system	1	2	3	4	5

XIV. Technology Usability and Satisfaction **(MILES study survey)**

These questions ask about your experience with the device or devices you received for the study.

1<br>Very Satisfied	2<br>Satisfied	3<br>Dissatisfied	4<br>Very Dissatisfied

___ **1.** Overall, how satisfied are you with your device(s)?

___ **2.** Overall, how easy/difficult was it to use your device(s)?

___ **3.** Overall, my device(s) interrupted my daily activities.

___ **4.** Overall, using my device(s) was an enjoyable experience.

___ **5.** Overall, using my device(s) required too much of my time.

___ **6.** Overall, my device(s) was distracting

___ **7.** Overall, my device(s) distracted me from my work.

___ **8.** Overall, my device(s) distracted me from my household chores.

___ **9.** Overall, my device(s) was uncomfortable to wear/carry around.

10. What do you like the most about your device(s)?

11. What do you like the least about device(s)??

12. I would be willing to participate in a study testing my device(s) again?

***XV. Satisfaction with Remote Monitoring Devices* (MILES study survey)**

We are interested in your experience with the Wired for Health devices. For the device(s) that you were given, please rate how much you agree or disagree with the following statements. 1 =strongly agree, to 6 = strongly disagree.

1	2	3	4	5	6
**Stongly Agree**	**Agree**	**Slightly Agree**	**Slightly Disagree**	**Disagree**	**Strongly Disagree**

___ **1.** I have found the remote monitoring device(s) easy to use.
___ **2.** The remote monitoring device(s) has been right on target for me.
___ **3.** The contacts I have had with the remote monitoring device(s) were too many.
___ **4.** I could set a personal goal for improving my health on my own than with the remote monitoring device(s).
___ **5.** The length of time needed to use the remote monitoring device(s) has been about right.
___ **6.** I have had a hard time remembering to use the remote monitoring device(s).
___ **7.** The remote monitoring device(s) has given me confidence that I could become more active in managing my condition.
___ **8.** The remote monitoring device(s) feedback has helped me track my health.
___ **9.** The remote monitoring device(s) has helped me track my exercise level.
___ **10.** The remote monitoring device(s) feedback has helped me track my eating.
___ **11.** There has been too little training on how to use the remote monitoring device(s).
___ **12.** The remote monitoring device(s) has motivated me to be active in monitoring my health.
___ **13.** The remote monitoring device(s) has motivated me to increase my exercise level.
___ **14.** The remote monitoring device(s) has motivated me to eat healthier foods.
___ **15.** The remote monitoring device(s) has helped me deal with problems I have had in achieving my health goals.
___ **16.** The remote monitoring device(s) has helped me remember to monitor my condition regularly.
___ **17.** I have found interactions with the remote monitoring device(s) to be boring
___ **18.** The remote monitoring device(s) has helped me understand the benefits of being active in managing my health.
___ **19.** The remote monitoring device(s) feedback I have received have made little difference in how much I have monitored my condition.

Appendix 21

Member Update

Scripps Medical Plans

Receive up to 32 Wellness Points for participating in the Wired for Health Study

Dear Member,

Thank you for your continued participation in the Disease Management program. We are pleased to be a part of your health care team.

We are sending you this letter to let you know about the new Scripps **Wired for Health Study** and to see if you might have interest in participating. This unique study is focusing on individuals who have diabetes, high blood pressure or a heart arrhythmia. It will test whether individuals who use remote wireless monitoring devices are better able to control their condition and decrease the cost of their health care over a six month period.

Study participants will be randomly assigned to either the control or monitoring group. Both groups will meet with a study staff member, complete health questionnaires and provide information about their current health conditions. The monitoring group will be provided with up to three devices. These devices include a blood pressure monitor, blood sugar monitor and/or heart monitor based on their current health conditions. All three devices will connect to an iPhone that will also be provided to the monitoring group for the duration of the study.

The **Scripps Wired for Health Study** is voluntary and will not cost you anything to participate. Please consider participating in this great cause!

We thank you for your time and wish you continued good health.

Sincerely,

Wellness Management
Phone: (800) 442-7247
Email: Wellness@HealthComp.com

HealthComp
Third Party Administrators

For more information or signing up:

Call a Health Comp Nurse at 1-800-442-7247 ext. 2507
or
Email: wellness@healthcomp.com

If we don't hear from you, a Nurse will call you in 2-4 weeks to see if you are interested or have any questions

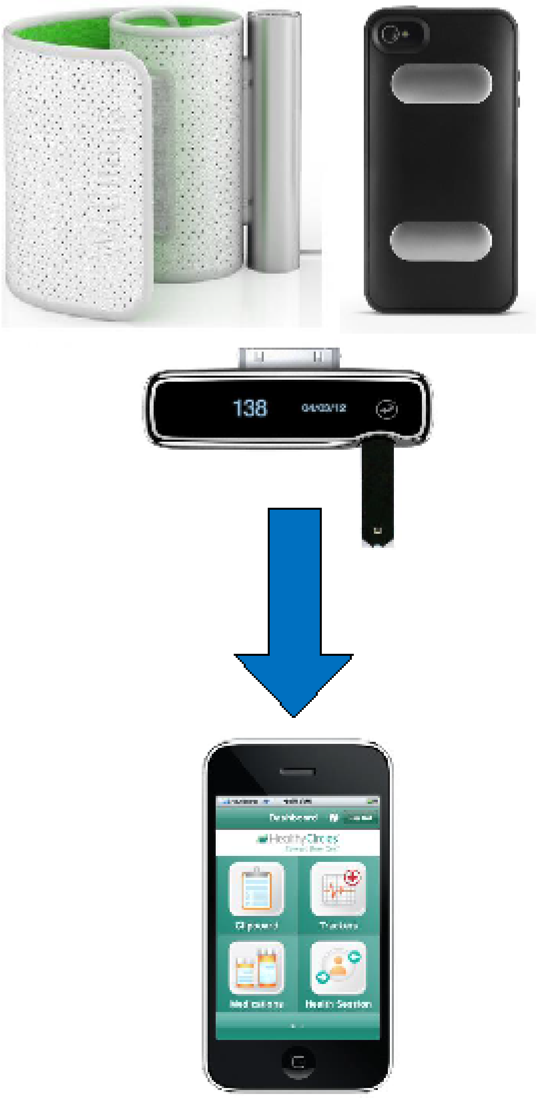

Scripps Translational Science Institute

Appendix 22

Dear Wired for Health Participant,

Thank you for your continued participation in the study. As mentioned at your enrollment visit, your participation in the Wired for Health Study provides an opportunity for you to earn wellness credits. The Scripps wellness program allows participants to complete activities in order to earn credit toward a reduction in health insurance costs. This year the number of credits required for the health insurance incentive has increased to 100. Therefore we are increasing the number of points that will be awarded for participation in the Wired for Health Study. There is a specific structural breakdown of how wellness points must be earned in order to qualify for this incentive. The reason for the specific structure of the program is to promote variety in activities. Please be aware that there is an annual maximum number of credits that can be earned in any given category. In the Wired for Health Study, you were randomized to either the control group or the monitoring group. Both groups will earn 32 wellness points for participation. Points will only be awarded to those who complete the study.

These wellness points will be earned in the following categories:

- 10 credits for Health Coaching (there is a **10 credit maximum** per year)
- 4 credits for Completion of the Baseline Survey and Follow Up Survey (there is a **4 credit maximum** for survey completion each year)
- 18 credits for Personal Health Activities (there is a **36 credit maximum** per year)

Your wellness points for the study will be awarded at the end of the fiscal year – September 2014. Furthermore, the data generated by the wireless devices is not part of your medical record unless you share that information with your healthcare provider.

If you have any questions or concerns on how to achieve the required number of wellness points, the HR team can help answer any questions that may arise for you. Attached you will find a table that shows a breakdown of wellness points and the maximum number for each category.

For general questions pertaining to the Scripps Wellness program contact:

Hamilton Mears
mears.hamilton@scrippshealth.org
(858) 309-2695

Andrea Krakower
krakower.andrea@scrippshealth.org
(858) 380-7445

Sincerely,

The Wired for Health Study Team

Appendix 23

You're Invited!

Wired for Health Study

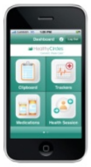

Details Inside

Scripps Translational Science Institute

You're Invited!

Wired for Health Study

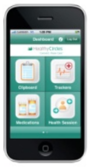

Details Inside

Scripps Translational Science Institute

You're Invited!

Wired for Health Study

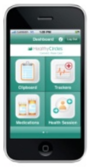

Details Inside

Scripps Translational Science Institute

Appendix 24

Dear Study Participant,

You have reached the end of the monitoring portion of the Wired for Health Study. Thank you very much for your participation. This study will help us answer some important questions about the management of chronic diseases and how wireless medical devices might help in that management. To close out your involvement in this study we need you to complete the end of study survey and attend a final study visit, which will be similar to the enrollment visit 6 months ago.

After you complete the survey, a study coordinator will call you to schedule your end of study visit.

Again, thank you for your participation in this important study.

Regards,

The Wired for Health Study Team

Appendix 25

HealthComp
Third Party Administrators
Wellness Management

April 28, 2014

Dear Member,

Please find enclosed two (2) copies of the Wired for Health Consent form. As you read it, you will find three (3) places for your signature. Sign in all three places, and return one copy in the self-addressed stamped envelope, and retain the other copy for your records. After we receive the consent, a link will be sent to your email address to complete the online survey. This survey must be done before a study coordinator will contact you for your appointment.

You may also fax the consent to us at (559) 243 7012. Please use a cover sheet and address it to "WFH" attention Catherine.

Thank you again for your participation in the Wired for Health Study.

Sincerely,

Catherine Rael | HealthComp
Utilization Management
P.O. Box 45018
Fresno, CA 93718-5018
Tel: (800) 442 7247 ext. 2544
Fax: (559)243 7012
