# Supplementary material for: A prospective randomized trial examining health care utilization in individuals using multiple smartphone-enabled biosensors
Source: PeerJ. 2016 Jan 14;4:e1554. doi: 10.7717/peerj.1554 (PMC4715435; doi:10.7717/peerj.1554)

**SUPPLEMENTAL METHODS**

***Inclusion Criteria***

1. Scripps Health insured employee or adult family member covered by Scripps Health plan
2. Ability to attend two visits (one at the beginning of the study and one at the end) at a Scripps facility
3. Computer and internet access, as well as ability to use e-mail and text messaging
4. Can grant permission for study staff to access medical records
5. Participating in Health Comp Disease Management program or willingness to join
6. English speaking
7. Within the past 12 months, a history of billing insurance for diagnostic codes consistent with diabetes, hypertension, and/or cardiac arrhythmia
8. 18 years and older
9. Willingness to use wireless devices, study iPhone, and learn to use the Healthy Circles platform.

***Exclusion Criteria***

1. Related to or household sharing with another study participant (if there are two or more people in one household that are eligible, the member that has the highest utilization will be invited to join first)
2. Change in living and/or employment situation that dictates the participant will no longer be covered by Scripps Health plan
3. Major surgery or extended trips in the next 6 months that may interfere with consistent use of monitoring device
4. Implanted ICD, pacemaker or implanted loop recorder or expected to undergo insertion during study
5. Arm circumference larger than that accommodated by Withings cuff
6. Unwilling or unable to grant informed consent
7. Pregnancy

**Figure S1.** Example display of Healthy Circles website showing blood pressure over time.

Source: Screenshot from http://connect.healthcomp.com.


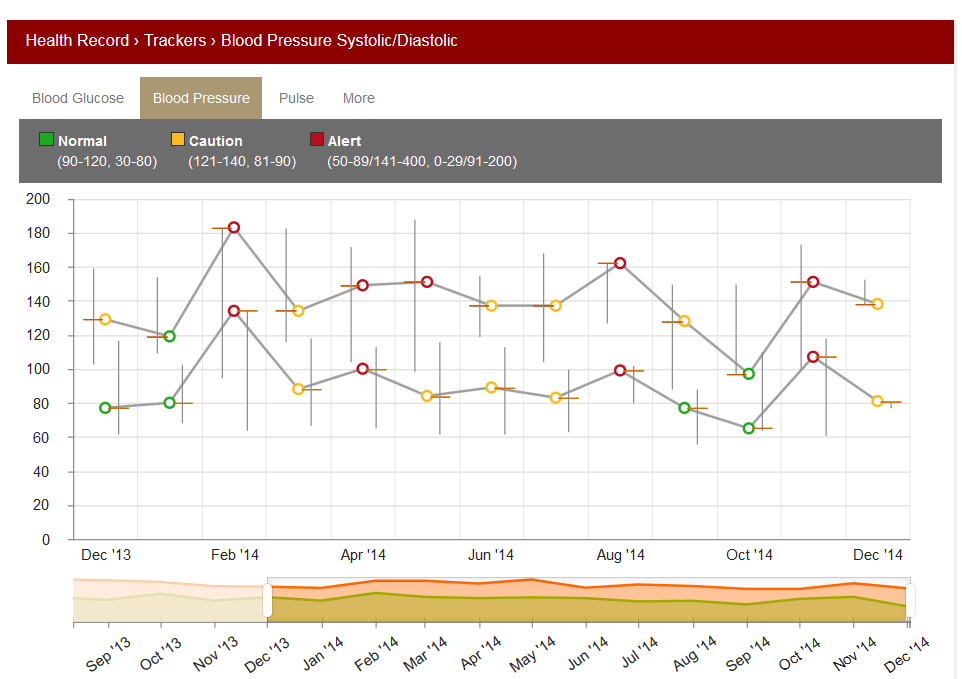


**Figure S2.** Example display of evaluation returned to user after submitting ECG to assess arrhythmia. Source: Screenshot from AliveCor PDF report.


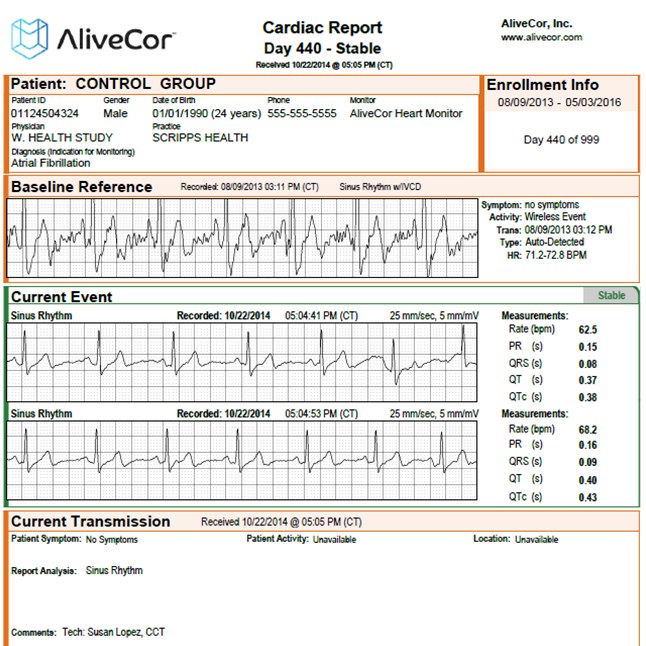


**Figure S3.** Example displays of Healthy Circles iPhone application. Source: Screenshots from iPhone application.


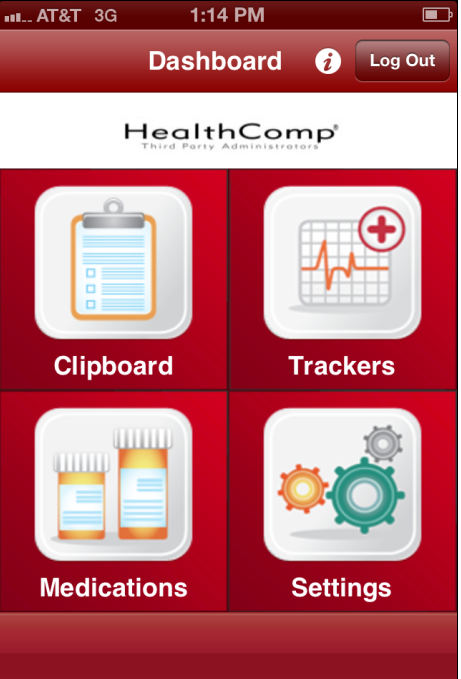

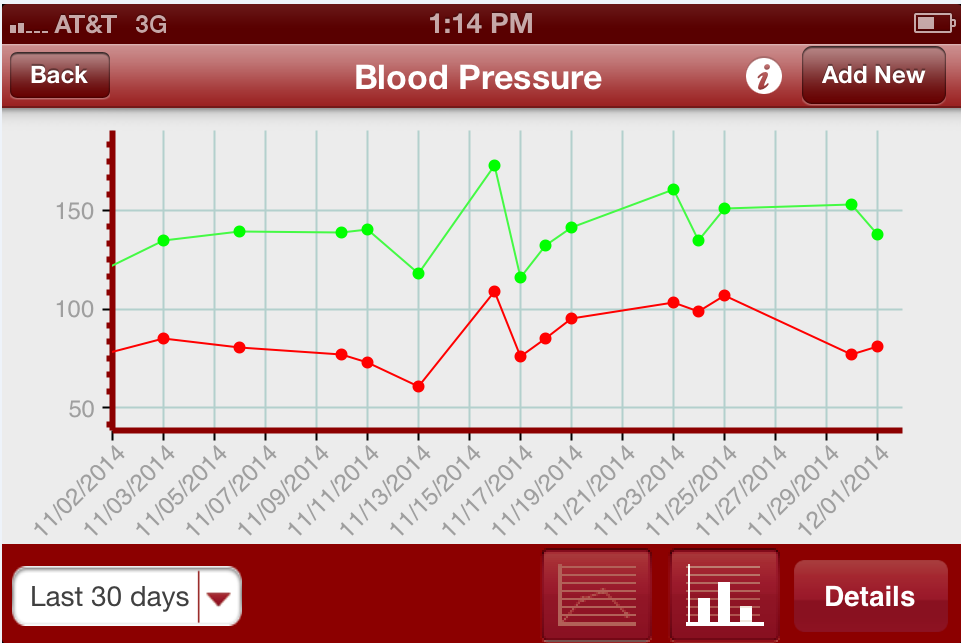


**Table S4.** Requested device usage frequency in the monitoring group.

| **Condition** | **Compliance** | **Poor Compliance** | **Patient Initiated Monitoring** |
| --- | --- | --- | --- |
| **Hypertension** | 2 x per day, 3 days per week, 1^st^ one in a.m. | < 3 x per week for 2 consecutive weeks | Symptoms including but not limited to visual disturbance, “bounding” pulse, chest discomfort, nausea |
| **NIDDM** | 1 x per day (pre-meal), 3 x per week | < 3 x per week in 1 week | Symptoms including but not limited to fatigue, visual changes, pre-syncopal symptoms, dyspnea, nausea, vomiting |
| **IDDM** | 4 x per day (pre-meal, with 4^th^ reading at bedtime, 2 hours after last meal/dinner), every day | < 4 x per day for 3 days in 1 week | Symptoms including but not limited to fatigue, visual disturbance, pre-syncopal symptoms, dyspnea, nausea, vomiting |
| **Arrhythmia** | if symptomatic | < 1 reading in 2 weeks will trigger message to confirm lack of symptoms, for 30 seconds | Symptoms including but not limited to chest discomfort, palpitations, rapid heart rate, feeling of “skipped beats,” dyspnea, nausea, pre-syncopal symptoms |

**Table S5.** Health insurance claims during a period of 6 months prior to study enrollment (baseline claims) between control and monitoring groups for all claims (dollars), office visits, emergency room visits, and inpatient stays. Mean (median); p-values uncorrected for multiple testing.

|  | Monitoring | Control | p-value |
| --- | --- | --- | --- |
| All Participants (N) | 75 | 85 | 0.47 |
| Claims ($) | 6,936 (1061) | 3,604 (784) | 0.23 |
| Office Visits | 4.2 (3) | 3.6 (2) | 0.36 |
| Emergency Room Visits | 0.03 (0) | 0.16 (0) | 0.06 |
| Inpatient Stays | 0.79 (0) | 0.21 (0) | 0.20 |
| Hypertension (N) | 67 | 71 | 0.29 |
| Claims ($) | 1,598 (93) | 458 (93) | 0.31 |
| Office Visits | 0.88 (1) | 0.65 (0) | 0.20 |
| Emergency Room Visits | 0 | 0 | NA |
| Inpatient Stays | 0.27 (0) | 0.07 (0) | 0.25 |
| NIDDM (N) | 10 | 17 | 0.26 |
| Claims ($) | 1,540 (330) | 198 (209) | 0.03 |
| Office Visits | 2.6 (2) | 0.9 (1) | 0.004 |
| Emergency Room Visits | 0 | 0 | NA |
| Inpatient Stays | 0 | 0 | NA |
| IDDM (N) | 10 | 10 | 0.76 |
| Claims ($) | 4,785 (317) | 1,067 (398) | 0.82 |
| Office Visits | 2.3 (2) | 1 (1) | 0.09 |
| Emergency Room Visits | 0 | 0 | NA |
| Inpatient Stays | 0.4 (0) | 0.1 (0) | 1.00 |
| Arrhythmia (N) | 10 | 19 | 0.14 |
| Claims ($) | 133 (26) | 4,464 (45) | 0.41 |
| Office Visits | 0.8 (0) | 1.1 (0) | 0.85 |
| Emergency Room Visits | 0 | 0.21 (0) | 0.32 |
| Inpatient Stays | 0 | 0.26 (0) | 0.21 |
| Comorbid (N) | 21 | 30 | 0.41 |
| Claims ($) | 6,127 (1,263) | 3,940 (895) | 0.87 |
| Office Visits | 5.1 (3) | 3.3 (3) | 0.28 |
| Emergency Room Visits | 0.05 (0) | 0.07 (0) | 0.80 |
| Inpatient Stays | 0.90 (0) | 0.3 (0) | 0.71 |

**Table S6.** Health insurance claims during a period of 6 months in study enrollment (enrollment claims) between control and monitoring groups for all claims (dollars), office visits, emergency room visits, and inpatient stays. Mean (median); p-values uncorrected for multiple testing. Includes all study participants who did not withdraw from study (i.e. completed or did not complete end-of-study assessment).

|  | Monitoring | Control | p-value |
| --- | --- | --- | --- |
| All Participants (N) | 69 | 82 | 0.33 |
| Claims ($) | 5,817 (845) | 5,347 (698) | 0.89 |
| Office Visits | 4.2 (3) | 3.7 (2) | 0.39 |
| Emergency Room Visits | 0.06 (0) | 0.04 (0) | 0.67 |
| Inpatient Stays | 0.36 (0) | 0.21 (0) | 0.51 |
| Hypertension (N) | 63 | 68 | 0.13 |
| Claims ($) | 557 (97) | 2,518 (79) | 0.38 |
| Office Visits | 1.3 (1) | 0.9 (1) | 0.10 |
| Emergency Room Visits | 0.03 (0) | 0.04 (0) | 0.81 |
| Inpatient Stays | 0 | 0.01 (0) | 0.34 |
| NIDDM (N) | 7 | 16 | 0.12 |
| Claims ($) | 350 (320) | 9,161 (158) | 0.13 |
| Office Visits | 1.7 (2) | 1.2 (1) | 0.23 |
| Emergency Room Visits | 0 | 0 | NA |
| Inpatient Stays | 0 | 0.06 (0) | 0.57 |
| IDDM (N) | 10 | 8 | 0.45 |
| Claims ($) | 628 (476) | 402 (293) | 0.10 |
| Office Visits | 2.3 (2) | 1.6 (1) | 0.33 |
| Emergency Room Visits | 0.1 (0) | 0 | 0.31 |
| Inpatient Stays | 0 | 0 | NA |
| Arrhythmia (N) | 9 | 18 | 0.20 |
| Claims ($) | 144 (89) | 6,036 (0) | 0.80 |
| Office Visits | 0.7 (0) | 0.8 (0) | 0.81 |
| Emergency Room Visits | 0.11 (0) | 0 | 0.18 |
| Inpatient Stays | 0 | 0.33 (0) | 0.53 |
| Comorbid (N) | 18 | 28 | 0.28 |
| Claims ($) | 6,930 (1,381) | 4,182 (932) | 0.96 |
| Office Visits | 4.9 (3) | 3.4 (3) | 0.21 |
| Emergency Room Visits | 0.05 (0) | 0.07 (0) | 0.85 |
| Inpatient Stays | 1.06 (0) | 0.32 (0) | 0.79 |

**Figure S7.** Study enrollment flowchart.

Total Insured 2012

N = 28,425

Chronic Disease Claim (Sampling Frame)

N = 3,998

At Least One Claim

N = 21,691

Declined Via Phone/Email

N = 1084

Never Reached

N = 2,637

Reconsider After Consent

N = 20

Ineligible

N = 10

Lost to Follow-up

N = 44

Reconsider Before Consent

N = 39

Agreed Via Phone/Email

N = 277

Cancelled Enrollment Visit

N = 4

Enrolled

N = 160

Control

N = 85

Monitoring

N = 75

Follow-up Complete

N = 65

Follow-up Complete

N = 65

**Figure S8.** Cumulative number of sessions to online disease management program at http://connect.healthcomp.com. The x-axis is time by date, and the y-axis is the cumulative number of sessions. Source: Authors’ analysis of Google Analytics website statistics.


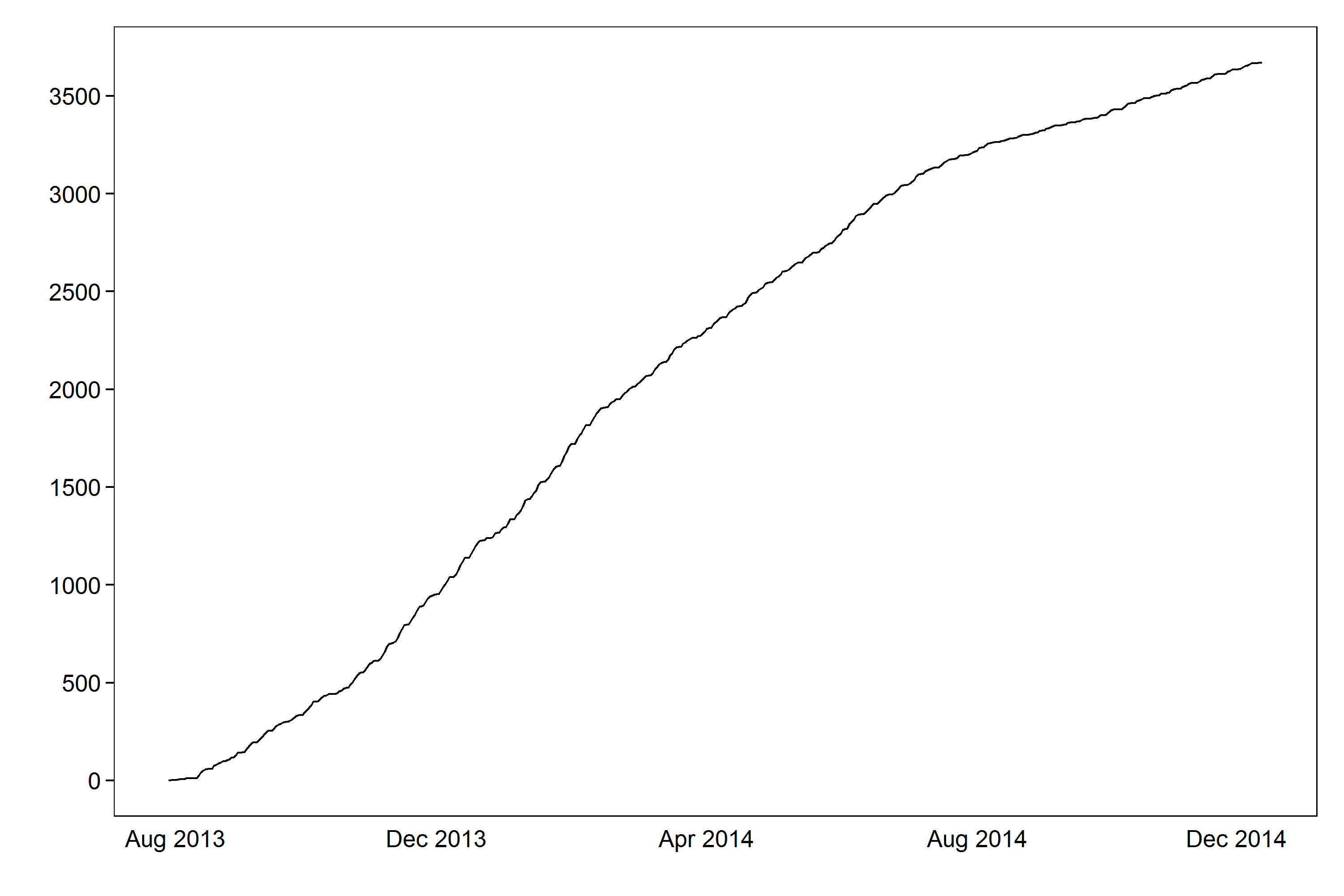


**Figure S9.** Cumulative density of device uses: Withings (red), IBGStar (blue), AliveCor (green). The x-axis is number of device uses by person, and the y-axis is the cumulative frequency. Source: Authors’ analysis of device usage.


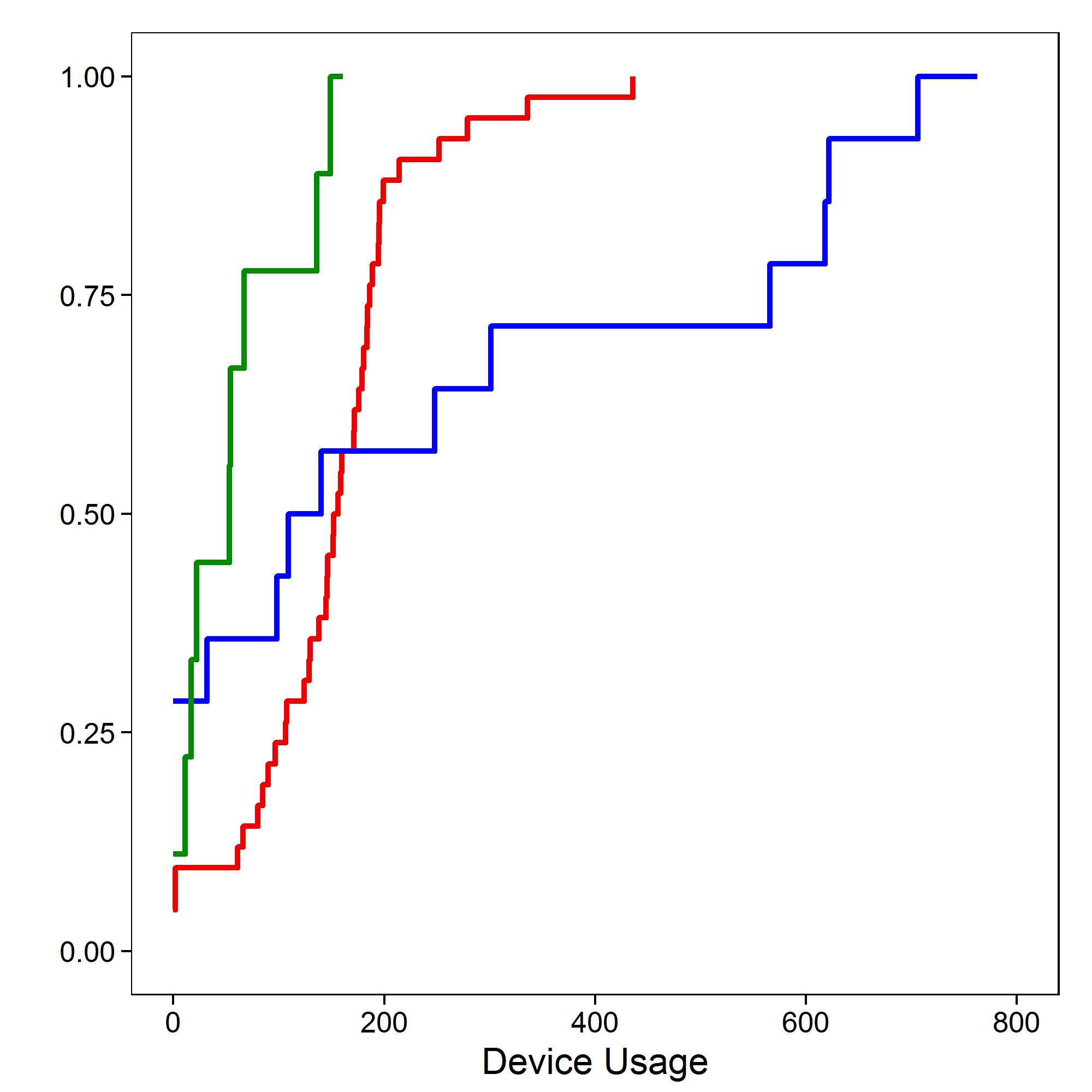

Supplement: Supplemental Information 3 [file peerj-04-1554-s003.docx]
